# Therapeutic inhibition of TRF1 impairs the growth of *p53*-deficient *K-Ras*^*G12V*^*-*induced lung cancer by induction of telomeric DNA damage

**DOI:** 10.15252/emmm.201404497

**Published:** 2015-05-13

**Authors:** María García-Beccaria, Paula Martínez, Marinela Méndez-Pertuz, Sonia Martínez, Carmen Blanco-Aparicio, Marta Cañamero, Francisca Mulero, Chiara Ambrogio, Juana M Flores, Diego Megias, Mariano Barbacid, Joaquín Pastor, Maria A Blasco

**Affiliations:** 1 Telomeres and Telomerase Group, Molecular Oncology Program, Spanish National Cancer Research Centre (CNIO)Madrid, Spain; 2 Experimental Therapeutics Program, Spanish National Cancer Research Centre (CNIO)Madrid, Spain; 3 Histopathology Unit, Biotechnology Program, Spanish National Cancer Research Centre (CNIO)Madrid, Spain; 4 Molecular Imaging Unit, Biotechnology Program, Spanish National Cancer Research Centre (CNIO)Madrid, Spain; 5 Experimental Oncology, Molecular Oncology Program, Spanish National Cancer Research Centre (CNIO)Madrid, Spain; 6 Animal Surgery and Medicine Department, Faculty of Veterinary Science, Complutense University of MadridMadrid, Spain; 7 Microscopy Unit, Biotechnology Program, Spanish National Cancer Research Centre (CNIO)Madrid, Spain

**Keywords:** cancer, drug development, shelterin, telomeres, TRF1

## Abstract

Telomeres are considered anti-cancer targets, as telomere maintenance above a minimum length is necessary for cancer growth. Telomerase abrogation in cancer-prone mouse models, however, only decreased tumor growth after several mouse generations when telomeres reach a critically short length, and this effect was lost upon *p53* mutation. Here, we address whether induction of telomere uncapping by inhibition of the TRF1 shelterin protein can effectively block cancer growth independently of telomere length. We show that genetic *Trf1* ablation impairs the growth of p53-null *K-Ras*^*G12V*^-induced lung carcinomas and increases mouse survival independently of telomere length. This is accompanied by induction of telomeric DNA damage, apoptosis, decreased proliferation, and G2 arrest. Long-term whole-body *Trf1* deletion in adult mice did not impact on mouse survival and viability, although some mice showed a moderately decreased cellularity in bone marrow and blood. Importantly, inhibition of TRF1 binding to telomeres by small molecules blocks the growth of already established lung carcinomas without affecting mouse survival or tissue function. Thus, induction of acute telomere uncapping emerges as a potential new therapeutic target for lung cancer.

## Introduction

Lung cancer is the leading cause of cancer-related deaths worldwide (Siegel *et al*, 2012). Adenocarcinoma, a subtype of non-small-cell lung cancer (NSCLC), is the single most common form of lung cancer, comprising 29% of cases (Herbst *et al*, 2008). The overall 5-year survival rate is of only 15%, due to the long-term ineffectiveness of current therapies and late stage at time of diagnosis (Siegel *et al*, 2012).

Activating mutations in the *K-Ras* proto-oncogene are found in 30% of human NSCLC (Rodenhuis *et al*, 1988). Mutations in the *p53* tumor suppressor gene are also common in NSCLC, affecting 50% of the cases (Chiba *et al*, 1990). Several lung cancer mouse models have been generated that recapitulate human NSCLC by using *K-Ras*-mutated alleles (Johnson *et al*, 2001; Guerra *et al*, 2003). In particular, the *lox-stop-lox-K-Ras*^*G12V*^ knock-in mouse model, in which endogenous expression of the *K-Ras*^*G12V*^ oncogene is induced upon Cre recombinase expression, has allowed the study of early stages of lung tumorigenesis (Guerra *et al*, 2003). Combination of *K-Ras*^*G12D*^ expression with p53 deficiency recapitulates late-stage lung cancers, including occurrence of invasion, stromal desmoplasia, and metastasis (Jackson *et al*, 2005). The *lox-stop-lox-K-Ras*^*G12V*^ mouse model has been instrumental to test novel therapeutic strategies against lung cancer, such as c-Raf, Cdk4, EGF receptor, and Notch (Puyol *et al*, 2010; Blasco *et al*, 2011; Maraver *et al*, 2012). However, to date all therapeutic targets tested have failed to impair the growth of *K-Ras*^*G12D*^-induced lung tumors in the context of p53 deficiency (Navas *et al*, 2012).

Telomeres are specialized heterochromatin structures at the ends of chromosomes composed of tandem TTA (Perera *et al*, 2008) GGG repeats bound by a protein complex, known as shelterin, that protects chromosome ends from degradation and DNA repair activities (Blasco, 2007; Palm & de Lange, 2008). The shelterin complex is composed of six core proteins, including the telomeric repeat binding factor 1 or TRF1 (de Lange, 2005).

During each cell division cycle, telomeres shorten owing to the incomplete replication of chromosome ends by conventional DNA polymerases, the so-called end-replication problem (Watson, 1972; Olovnikov, 1973). Telomerase can elongate telomeres *de novo*, thus compensating for telomere shortening in those cells where it is expressed, such as pluripotent and adult stem cells, as well as the majority of late-stage human cancers (Shay & Bacchetti, 1997; Deng & Chang, 2007; Shay & Wright, 2010). Telomerase is composed of a catalytic subunit or *TERT* and of an RNA component or *Terc* which is used as template for the *de novo* synthesis of telomeric repeats (Greider & Blackburn, 1985).

Telomeres are usually shorter in tumor cells compared to the healthy surrounding tissue (de Lange *et al*, 1990; Shay & Wright, 2010). To maintain a minimum functional telomere length, 80–90% of human tumors reactivate telomerase (Kim *et al*, 1994; Shay & Bacchetti, 1997; Joseph *et al*, 2010), and the remaining activate an alternative mechanism to maintain telomeres, known as ALT, based on recombination between telomeric sequences (Bryan *et al*, 1997). Supporting a role of telomerase deregulation in human cancer, single nucleotide polymorphisms in the locus of human *hTERT* are associated with various malignancies, including glioma, lung cancer, urinary bladder cancer, melanoma, and breast cancer, among others (McKay *et al*, 2008; Wang *et al*, 2008; Rafnar *et al*, 2009; Shete *et al*, 2009; Petersen *et al*, 2010; Melin *et al*, 2012; Mocellin *et al*, 2012; Bojesen *et al*, 2013; Garcia-Closas *et al*, 2013; Horn *et al*, 2013; Huang *et al*, 2013). These findings lead to the development of telomerase-based therapeutic strategies for cancer treatment, some of which are currently tested in clinical trials (Buseman *et al*, 2012).

Telomerase abrogation in the context of mouse models, however, has only shown anti-tumorigenic activity after several mouse generations of telomerase-deficient *Terc*^−/−^ mice, when telomeres reach a critically short length, and this anti-tumorigenic effect is abrogated in the absence of p53 (Chin *et al*, 1999; Greenberg *et al*, 1999). Similarly, in the context of the *K-Ras*^*G12V*^ lung carcinogenesis mouse model, telomerase deficiency decreased tumor growth only after five mouse generations, and this effect was lost upon p53 abrogation (Perera *et al*, 2008).

To circumvent these potential shortcomings of telomerase inhibition, here we set out to address the therapeutic effect of acute telomere uncapping owing to *Trf1* abrogation (Martinez *et al*, 2009) in the *K-Ras*^*G12V*^ lung cancer model (Guerra *et al*, 2003). TRF1 is an essential component of shelterin and, in addition, it is enriched in adult stem cells and pluripotent stem cells, suggesting that its inhibition may also target cancer stem cells (Boue *et al*, 2010; Schneider *et al*, 2013). In this regard, we have previously shown that TRF1 deletion in stratified epithelia could promote cancer development when in a p53-deficient background (Martinez *et al*, 2009). However, in contrast to POT1, another shelterin component, TRF1, has not been found mutated in human cancer (Ramsay *et al*, 2013; Robles-Espinoza *et al*, 2014; Shi *et al*, 2014; Bainbridge *et al*, 2015). Indeed, TRF1 has been reported to be overexpressed in several tumor types (Matsutani *et al*, 2001; Ohyashiki *et al*, 2001; Fujimoto *et al*, 2003; Oh *et al*, 2005; Bellon *et al*, 2006). Thus, here we set out to address whether *Trf1* deletion in the context of oncogenic *K-Ras*-induced lung cancer mouse model would act as a tumor suppressor or as an oncogene.

We found that *Trf1* genetic ablation effectively reduces the size and malignancy of p53-null *K-Ras*^*G12V*^ lung carcinomas and increases mouse survival. This tumor-suppressive effect of *Trf1* deficiency occurs already at the first mouse generation and is independent of telomere length. Furthermore, long-term conditional whole-body *Trf1* deletion in adult mice does not affect mouse viability and survival. Moreover, we show that chemical inhibition of TRF1 can be achieved *in vivo* by using small molecules, which effectively impair the growth of already established lung adenocarcinomas without affecting mouse and tissue viability. Thus, acute telomere uncapping owing to TRF1 inhibition represents a novel potent therapeutic strategy for *K-Ras*-induced lung cancer.

## Results

### *Trf1* deficiency impairs immortalization of MEFs expressing the *K-Ras*^*G12V*^ oncogene even in a p53-deficient background

**Figure 1:**
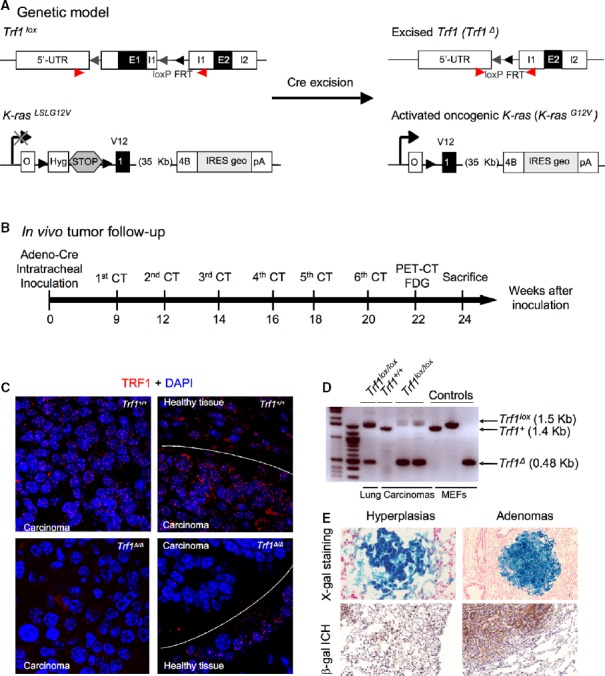
Efficient oncogenic *K-Ras*^*G12V*^ expression and *Trf1* depletion in lung lesions

To assess the effect of *Trf1* abrogation in the context of lung cancer induced by expression of the *K-Ras*^*G12V*^ oncogene, we crossed *K-Ras*^+*/LSLG12Vgeo*^ mice (designated from now on as *K-Ras*^+*/G12V*^) (Guerra *et al*, 2003) to a strain carrying a floxable allele of *Trf1* (*Trf1*^*lox/lox*^) either wild-type or deficient for *p53* (*p53*^−/−^) (Martinez *et al*, 2009) (Fig.1A). First, we isolated primary (passage 2) mouse embryonic fibroblasts (MEFs) and transduced them with a retrovirus encoding the Cre recombinase (pBabe-Cre). This allowed the expression of the resident K-Ras^G12V^ oncoprotein simultaneously with the deletion of exon 1 of the *Trf1*^*lox*^ allele (Fig1A). A *p53*-null background was used to allow for the growth of *Trf1*-deleted cells, which otherwise show severe proliferative defects (Supplementary Fig S1A) (Martinez *et al*, 2009; Thanasoula *et al*, 2010). While *Trf1*^+*/*+^ *K-Ras*^+*/G12V*^ *p53*^−/−^ MEFs showed a 16-fold increase in cell number at day 7 post-plating, *Trf1*^Δ/Δ^ *K-Ras*^+*/G12V*^ *p53*^−/−^ MEFs only increased their population by fourfold in the same time (Supplementary Fig S1A), indicating a severe growth impairment of *Trf1*-deficient *K-Ras*^G12V^-expressing cells compared to the *Trf1*^+*/*+^ *K-Ras*^+*/G12V*^ *p53*^−/−^ controls.

Genetic model. *Trf1*^*lox*^ and *K-Ras*^*LSLGV*^^*12*^ alleles are depicted before and after Cre-mediated excision.

*In vivo* imaging schedule. Eight- to ten-week-old mice were intratracheally infected with adeno-Cre, mice were analyzed every 2 weeks by computerized tomography (CT), and 22 weeks post-infection, a positron emission tomography (PET) was performed. Mice were sacrificed 24 weeks post-infection for further histological analysis.

TRF1 immunofluorescence of the lungs. Notice the absence and presence of TRF1 signal in the carcinomas and surrounding healthy tissue of *Trf1*^Δ/Δ^ mice, respectively.

Analysis of *Trf1* excision by PCR. Notice the completed excision in carcinomas of *Trf1*^*lox/lox*^ lungs.

Detection of β-galactosidase activity in the lungs as a surrogate marker of oncogenic *K-Ras*^*G12V*^ expression.

To address how *Trf1* ablation impairs the growth of *K-Ras*^*G12V*^-expressing MEFs, we first analyzed cellular senescence by the β-galactosidase senescence-associated activity. *Trf1*^Δ/Δ^ *K-Ras*^+*/G12V*^ *p53*^+*/*+^ MEFs presented 1.9-fold higher percentage of senescent cells 7 days post-plating compared to *Trf1*^+*/*+^ *K-Ras*^+*/G12V*^ *p53*^+*/*+^ MEFs (Supplementary Fig S1B) (Martinez *et al*, 2009). Of note, p53 deficiency did not abolish *Trf1* deficiency-mediated senescence. Indeed, *Trf1*^Δ/Δ^ *K-Ras*^+*/G12V*^ *p53*^−/−^ MEFs showed a 21-fold higher percentage of senescent cells compared to *Trf1*^+*/*+^ *K-Ras*^+*/G12V*^ *p53*^−/−^ MEFs 7 days post-plating (Supplementary Fig S1B), most likely reflecting an additive effect of *K-Ras* oncogene-induced senescence and *Trf1* deficiency-induced senescence (Serrano *et al*, 1997; Martinez *et al*, 2009). No significant differences in apoptosis were detected between genotypes by caspase-3 activation (Supplementary Fig S1C). Thus, *Trf1* abrogation in MEFs expressing mutant *K-Ras* leads to higher numbers of senescent cells even in the absence of p53.

Next, we addressed the effect of *Trf1* abrogation in immortalization of MEFs. To this end, we performed a colony formation assay, which reflects on the clonogenic capacity of individual cells. p53-proficient MEFs did not form any colonies in agreement with the fact that *p53* wild-type MEFs do not spontaneously immortalize (Supplementary Fig S1D and E) (Harvey & Levine, 1991; Parrinello *et al*, 2003). In contrast, p53-deficient MEFs were able to form immortalized colonies, although *Trf1*^Δ/Δ^ *K-Ras*^+*/G12V*^ *p53*^−/−^ MEFs formed fewer and smaller colonies than *Trf1*^+*/*+^ *K-Ras*^+*/G12V*^ *p53*^−/−^ MEFs. In summary, *Trf1* deficiency limits both proliferation and cellular immortalization of *K-Ras*-expressing cells *in vitro* even in the absence of p53.

### *Trf1* deficiency impairs *K-Ras*^*G12V*^-mediated lung cancer and increases mouse survival even in the absence of p53

We next set out to determine the impact of *Trf1* deficiency *in vivo* in the *K-Ras*-induced lung carcinogenesis model. To this end, 8-week-old *Trf1*^*lox/lox*^ *K-Ras*^+*/G12V*^ *p53*^+*/*+^ and *Trf1*^*lox/lox*^ *K-Ras*^+*/G12V*^ *p53*^−/−^ mice, as well as their respective *Trf1* wild-type controls, were infected by intratracheal instillation with replication-defective adenoviruses encoding the Cre recombinase (adeno-Cre) (Materials and Methods). This strategy allowed the expression of the resident K-Ras^G12V^ oncoprotein simultaneously with the ablation of the *Trf1*^*lox*^ allele in the infected lung cells (Fig1A). Nine weeks after viral inoculation, tumor growth was measured by using computed tomography (CT) every second week until 24^th^ week post-infection when the experiment was concluded. At 22 weeks post-infection, positron emission tomography (PET) was performed to monitor tumor malignancy (Fig1B). At 24^th^ week post-infection, the animals were sacrificed to carry a full histopathological analysis of the lungs, and to confirm *K-Ras*^*G12V*^ expression and *Trf1* deletion in the lesions (Fig1B). *Trf1* deletion was monitored in all tumors by TRF1 immunofluorescence and by PCR (Fig1C and D). *K-Ras*^*G12V*^ expression in tumors was confirmed by detecting the expression of its beta-galactosidase (β-geo) reporter (Fig1E).

**Figure 2:**
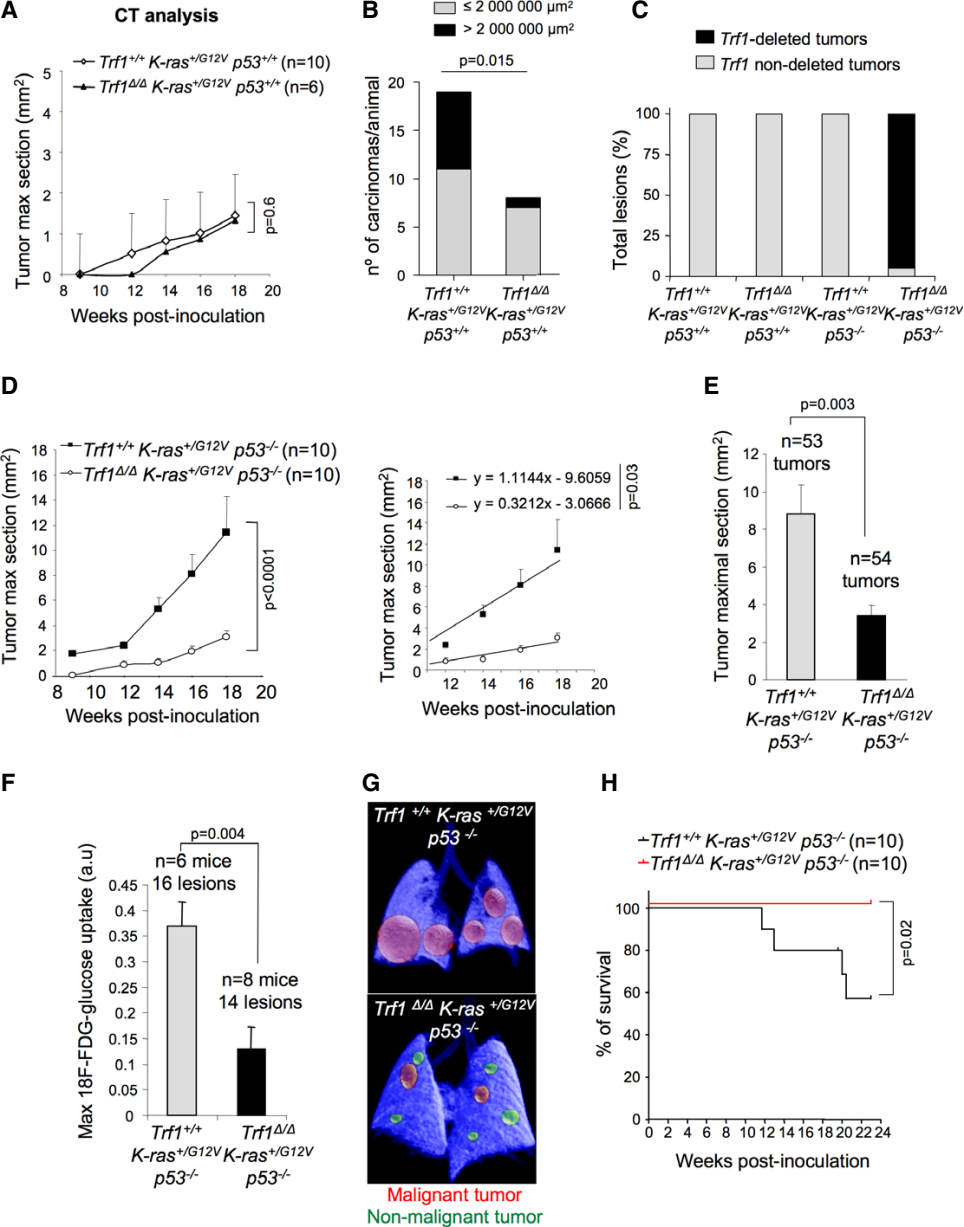
*Trf1* deficiency impairs *K-Ras*-mediated lung cancer development

*In vivo* tumor follow-up by CT scan showed that in a p53-proficient background, *Trf1*-deleted mice showed a delayed onset of the first CT scan-detectable lesions from 9 weeks in *Trf1* wild-type lungs to 12 weeks in the *Trf1*-deleted ones (Fig2A). However, after this initial lag, both genotypes showed a similar growth of the tumor lesions by CT scan (Fig2A). *Post-mortem* lung analysis revealed that the number of tumors per mouse was higher in *Trf1* wild-type than in *Trf1*^*lox/lox*^ mice although tumors were histologically identical (Fig2B). Importantly, immunofluorescence analysis of *Trf1* expression showed that all tumors in *Trf1*-deleted lungs were escapers and had normal *Trf1* expression (Fig2C). Thus, *Trf1* is essential for *K-Ras*^*G12V*^-induced lung tumor development in a p53-proficient background as no tumors lacking *Trf1* expression were found (Fig2C).

Tumor growth curve of *Trf1*^+/+^ *K-Ras*^+*/G12V*^ *p53*^+*/*+^ and *Trf1*^Δ/Δ^ *K-Ras*^+*/G12V*^ *p53*^+*/*+^ measured by computed tomography (CT).

Quantification of the number and size of *Trf1*^+*/*+^ *K-Ras*^+*/G12V*^ *p53*^+*/*+^ and *Trf1*^Δ/Δ^ *K-Ras*^+*/G12V*^ *p53*^+*/*+^ carcinomas at death point.

Percentage of tumors that have deleted *Trf1* quantified by TRF1 immunofluorescence after mice had been sacrificed. Post-mortem analysis of *Trf1* deletion in each tumor revealed that none of the *Trf1*^*lox/lox*^ *K-Ras*^+*/G12V*^ *p53*^+*/*+^ ones had excised *Trf1*.

Tumor growth curve and tumor growth slope of *Trf1*^+*/*+^ *K-Ras*^+*/G12V*^ *p53*^−/−^ and *Trf1*^Δ/Δ^ *K-Ras*^+*/G12V*^ *p53*^−/−^ measured by CT.

Tumor maximal section of *Trf1*^+*/*+^ *K-Ras*^+*/G12V*^ *p53*^−/−^ and *Trf1*^Δ/Δ^ *K-Ras*^+*/G12V*^ *p53*^−/−^ lungs measured by histological analysis before death point by CT.

Maximum 18F-FDG-glucose uptake by *Trf1*^+*/*+^ *K-Ras*^+*/G12V*^ *p53*^−/−^ and *Trf1*^Δ/Δ^ *K-Ras*^+*/G12V*^ *p53*^−/−^ tumors 22 weeks after infection by positron emission tomography (PET).

Representative PET-CT image of *Trf1*^+*/*+^ *K-Ras*^+*/G12V*^ *p53*^−/−^ and *Trf1*^Δ/Δ^ *K-Ras*^+*/G12V*^ *p53*^−/−^ lungs.

Survival curve of *Trf1*^+*/*+^ *K-Ras*^+*/G12V*^ *p53*^−/−^ and *Trf1*^Δ/Δ^ *K-Ras*^+*/G12V*^ *p53*^−/−^ mice. Data information: Error bars represent standard error. *t*-test, chi-squared (B) test, or log-rank (Mantel–Cox; H) test was used to assess statistical significance. The number of mice and the number of tumors are indicated in each case.

Next, we studied the impact of *Trf1* deletion in *K-Ras*^*G12V*^-induced lung tumors in a p53-deficient background, a situation that resembles many human lung tumors. CT analysis revealed that *Trf1*^Δ/Δ^ *K-Ras*^+*/G12V*^ *p53*^−/−^ tumors grew markedly slower and reached a smaller size at their end point than *Trf1*^+*/*+^ *K-Ras*^+*/G12V*^ *p53*^−/−^ tumors (Fig2D and E). PET analysis at 22 weeks post-infection revealed that *Trf1*^Δ/Δ^ *K-Ras*^+*/G12V*^ *p53*^−/−^ tumors showed less metabolic activity than *Trf1*^+*/*+^ *K-Ras*^+*/G12V*^ *p53*^−/−^ tumors indicating a lower grade of malignancy (Fig2F and G). Notably, in agreement with the lower malignancy, *Trf1*^*lox/lox*^ *K-Ras*^+*/G12V*^ *p53*^−/−^ mouse survival was significantly higher than that of *Trf1*^+*/*+^ *K-Ras*^+*/G12V*^ *p53*^−/−^ mice (Fig2H). Of note, in this setting only 5% of the *Trf1*^*lox/lox*^ *p53*^−/−^ tumors were found to be escapers (Fig2C).

By blind histopathological analysis of the lungs, we confirmed that *Trf1*^Δ/Δ^ *K-Ras*^+*/G12V*^ *p53*^−/−^ lungs developed less carcinomas than *Trf1*^+*/*+^ *K-Ras*^+*/G12V*^ *p53*^−/−^ lungs (Supplementary Fig S2A–C). In addition, the malignant lesions in *Trf1*^Δ/Δ^ *K-Ras*^+*/G12V*^ *p53*^−/−^ lungs were smaller compared to *Trf1*^+*/*+^ *K-Ras*^+*/G12V*^ *p53*^−/−^ lungs (Supplementary Fig. S2A–C). In summary, *Trf1* deletion effectively impairs progression to full-blown carcinomas even in the absence of p53.

### *Trf1* abrogation induces telomeric DNA damage and apoptosis in p53-deficient lung carcinomas

**Figure 3:**
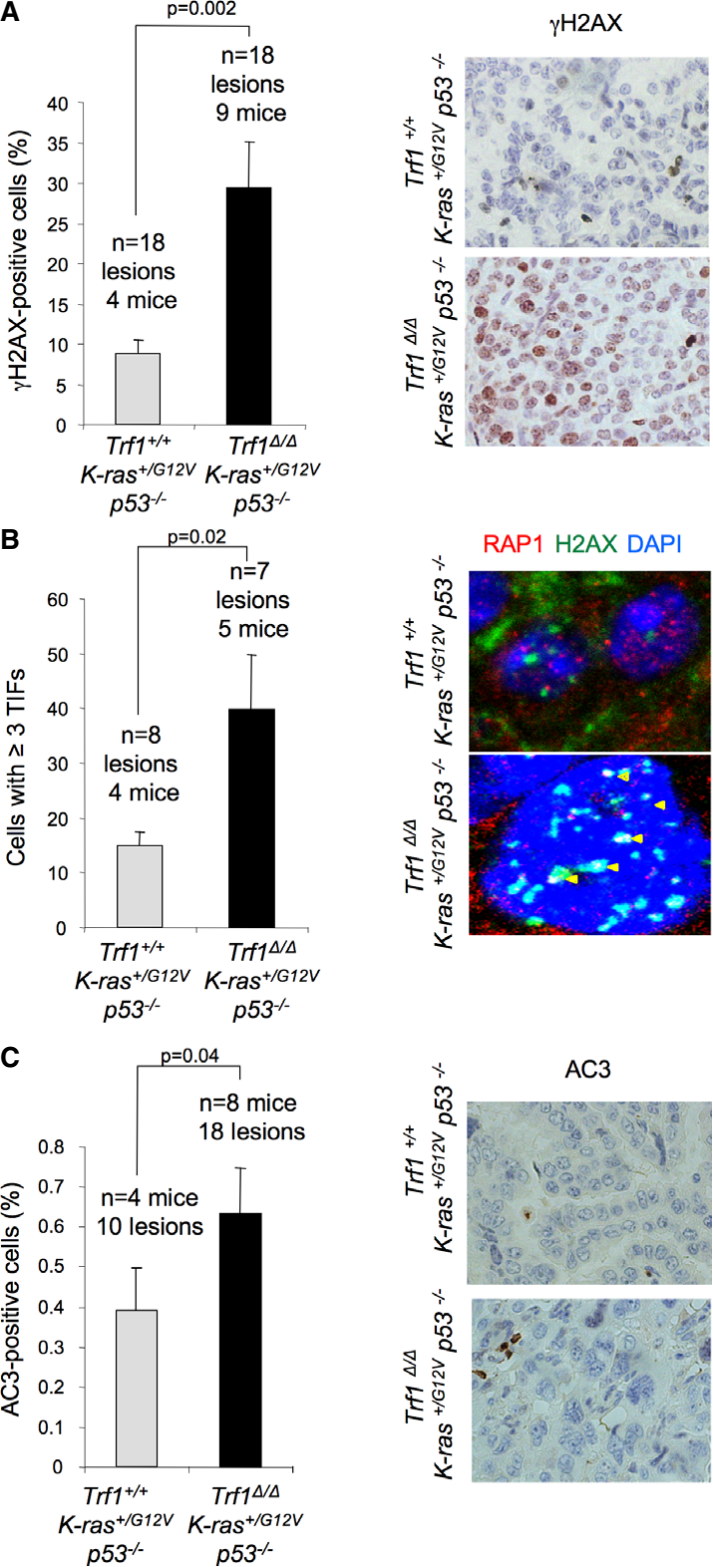
*Trf1*-deficient carcinomas present high amount of telomeric DNA damage and apoptosis

Previous reports have shown that abrogation of *Trf1* in different cell types causes a persistent DNA damage response at chromosome ends, which leads to decreased cell viability (Martinez *et al*, 2009; Sfeir *et al*, 2009; Beier *et al*, 2012; Schneider *et al*, 2013). To address whether the decreased growth and lower malignancy of *Trf1-*deficient lung tumors were associated with increased DNA damage in the lesions, we quantified γH2AX DNA damage foci and their colocalization to telomeres (the so-called telomere-induced foci or TIFs) in lung carcinoma sections. The percentage of γH2AX-positive cells was significantly higher in *Trf1*^Δ/Δ^ *K-Ras*^+*/G12V*^ *p53*^−/−^ carcinomas compared to *Trf1*^+*/*+^ *K-Ras*^+*/G12V*^ *p53*^−/−^ carcinomas (Fig3A). Furthermore, co-immunofluorescence staining of γH2AX foci with RAP1, another shelterin component localized at telomeres, showed increased number of DNA damage foci at telomeres in *Trf1*^Δ/Δ^ *K-Ras*^+*/G12V*^ *p53*^−/−^ than in *Trf1*^+*/*+^ *K-Ras*^+*/G12V*^ *p53*^−/−^ carcinomas (Fig3B).

Percentage of cells showing γH2AX foci in carcinomas of the indicated genotypes (left panel). Representative images of γH2AX immunohistochemistry (right panel).

Percentage of cells showing 3 or more γH2AX and RAP1 colocalizing foci (TIFs) (left panel). Representative images of γH2AX and RAP1 double immunofluorescence (right panel). Yellow arrowheads: colocalization of γH2AX and RAP1.

Percentage of active caspase-3 (AC3)-positive cells (left panel). Representative images of AC3 immunohistochemistry (right panel). Data information: Error bars represent standard error. The number of mice and carcinomas analyzed per genotype is indicated. *t*-test was used to assess statistical significance.

To address the cellular effects of increased telomere damage in lung carcinomas, we first determined the percentage of apoptotic cells in lung carcinoma lesions. The percentage of carcinoma cells that were positive for the apoptotic marker active caspase-3 (AC3) was higher in *Trf1*^Δ/Δ^ *K-Ras*^+*/G12V*^ *p53*^−/−^ carcinomas compared to *Trf1*^+*/*+^ *K-Ras*^+*/G12V*^ *p53*^−/−^ carcinomas (Fig3C). Thus, *Trf1* deletion in lung carcinomas leads to increased telomeric damage and subsequent induction of apoptosis.

### *Trf1* deficiency leads lower proliferation and increased G2 arrest and mitotic defects in p53-deficient lung carcinomas

**Figure 4:**
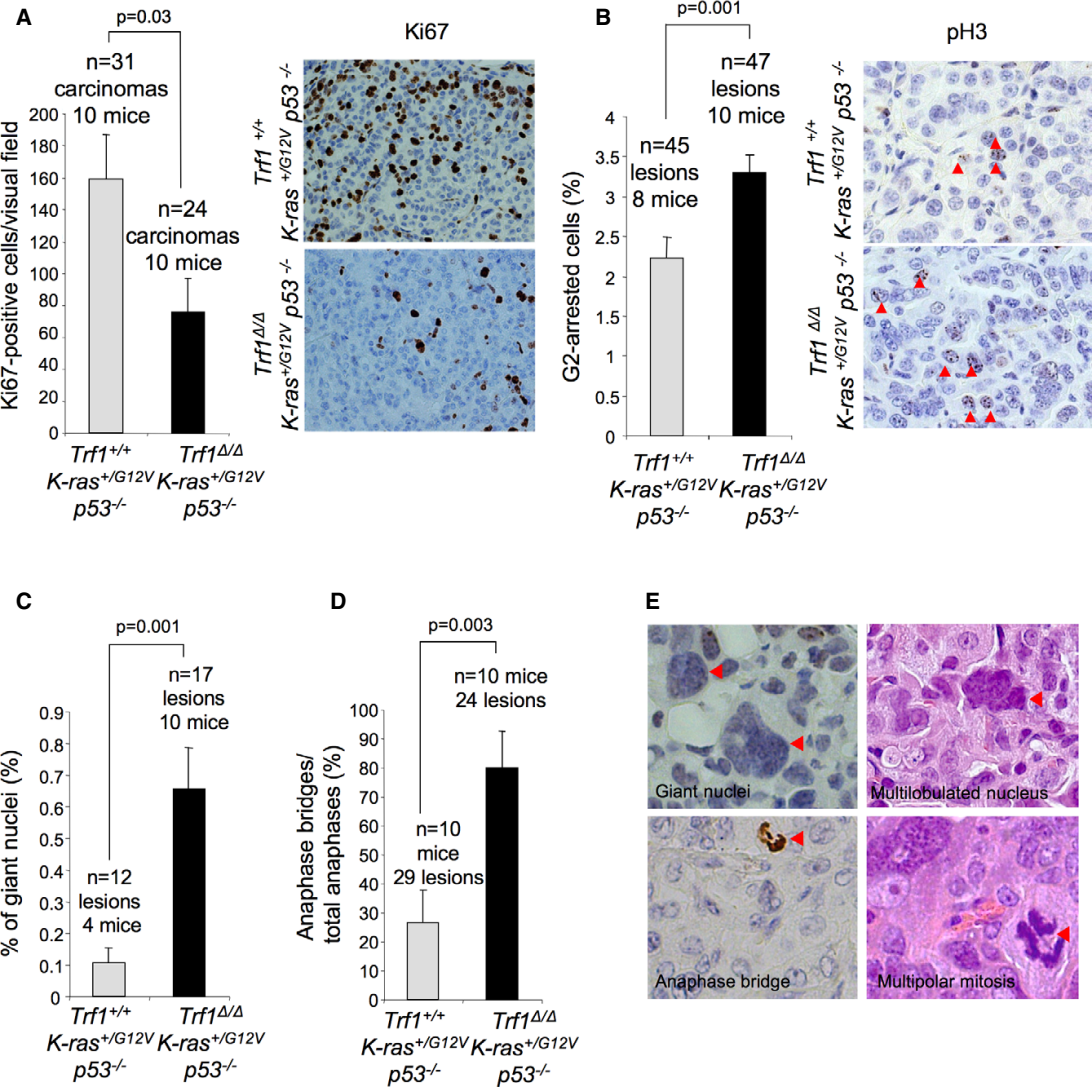
*Trf1* deficiency leads to G2 arrest and mitotic defects

To determine whether *Trf1* deletion in the context of *K-Ras*^*G12V*^-induced lung cancer also leads to proliferation defects, we performed Ki67 immunohistochemistry directly on lung carcinoma sections. We observed a lower proliferation index (Ki67-positive cells) in *Trf1*^Δ/Δ^ *K-Ras*^+*/G12V*^ *p53*^−/−^ carcinomas compared to *Trf1*^+*/*+^ *K-Ras*^+*/G12V*^ *p53*^−/−^ carcinomas (Fig4A). To determine the cell cycle phase where *Trf1*-deleted cells showed defects, we analyzed the staining pattern of phospho-histone H3 (Ser10). A pH3 pan-nuclear staining is a distinctive feature of mitotic cells, whereas pH3 foci are characteristic of G2 cells (Hendzel *et al*, 1991). We found that the percentage of cells positive for G2-distinctive pH3 foci pattern was significantly increased in *Trf1*^Δ/Δ^ *K-Ras*^+*/G12V*^ *p53*^−/−^ carcinoma lesions compared to *Trf1*^+*/*+^ *K-Ras*^+*/G12V*^ *p53*^−/−^ lesions (Fig4B), suggestive of increased G2 arrest.

Percentage of Ki67-positive cells in the carcinomas of the indicated genotypes (left panel). Representative images of Ki67 immunohistochemistry (right panel).

Percentage of pH3-positive cells in the carcinomas of the indicated genotypes (left panel). Representative images of pH3 immunohistochemistry (right panel). Red arrowheads: pH3-positive cells.

Percentage of giant nuclei in the carcinomas of the indicated genotype.

Percentage of anaphase bridges out of total anaphases in the carcinomas of the indicated genotypes.

Representative images of giant nuclei, multilobulated nuclei, anaphase bridges, and multipolar mitoses. Red arrowheads indicate the corresponding mitotic aberrations indicated in the image. Data information: Error bars represent standard error. The number of mice and carcinomas analyzed per genotype is indicated. *t*-test was used to assess statistical significance.

Persistent telomere damage can result in bypass of mitosis leading to endoreduplication and tetraploidy (Davoli *et al*, 2010). In line with this, *Trf1*^Δ/Δ^ *K-Ras*^+*/G12V*^ *p53*^−/−^ carcinomas presented an increased number of giant nuclei indicative of endoreduplication, as well as increased anaphase bridges, compared to *Trf1*^+*/*+^ *K-Ras*^+*/G12V*^ *p53*^−/−^ carcinomas (Fig4C–E). Similarly, *Trf1*^Δ/Δ^ *K-Ras*^+*/G12V*^ *p53*^−/−^ carcinomas presented cells showing bizarre multilobulated nuclei and multipolar mitosis, which were not present in *Trf1*^+*/*+^ *K-Ras*^+*/G12V*^ *p53*^−/−^ carcinomas (Fig4E). This type of aberrant nuclei have been previously related to mitotic catastrophes (Vakifahmetoglu *et al*, 2008). These observations suggest that lung carcinoma cells require TRF1 for proper completion of mitosis and that *Trf1* deletion leads to severe mitotic defects.

### *Trf1* downregulation in mouse cell lines derived from *K-Ras*^*G12V*^ p53-deficient lung carcinomas as well as in human lung cancer cell lines impairs cancer growth and metastasis in xenograft models

**Figure 5:**
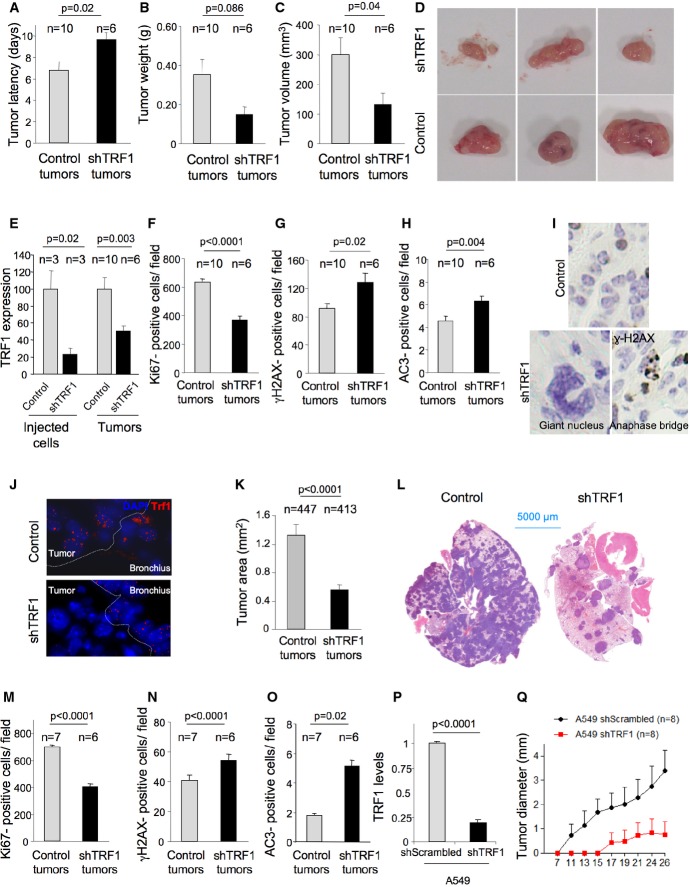
*Trf1* downregulation in *K-Ras*^*G12V*^-transformed lung cells leads to a decreased tumor growth and decreased metastatic potential in allograft and xenograft models

To validate these results using an independent strategy to inhibit TRF1, as well as to assess the effect of TRF1 abrogation in already established *K-Ras*-induced lung tumors, we downregulated *Trf1* expression by using shRNA technology in three *K-Ras*^Δ*/G12Vgeo*^ *p53*^−/−^ mouse cancer cell lines derived from three independent mouse lung carcinoma lesions and assessed the effects on tumor growth using two independent allograft experiments. First, to address the effect of TRF1 inhibition in tumor growth *in vivo*, we subcutaneously injected 50,000 lung carcinoma cells into immunodeficient mice and followed tumor onset and growth. *Trf1* downregulation resulted in a marked delay in tumor onset as well as in a significantly decreased tumor growth (Fig5A–D). Three weeks after injection, mice were sacrificed and the tumors were histologically analyzed. We confirmed that *Trf1* downregulation was maintained during *in vivo* tumor development (Fig5E). *Trf1*-downregulated tumors showed decreased proliferation and increased DNA damage as well as increased apoptosis compared to controls (Fig5F–H). Again, *Trf1*-downregulated tumors presented a high proportion of aberrant nuclei and anaphase bridges (Fig5I). Next, to study the effect of TRF1 inhibition in the metastatic potential of established lung cancer cells induced by *K-Ras* expression, we intravenously injected 150,000 *K-Ras*^Δ*/LG12Vgeo*^ *p53*^−/−^ lung cells into immunodeficient mice. Tail vein injection of tumor cells results in lung metastasis (Elkin & Vlodavsky, 2001). Three weeks after injection, the mice were sacrificed and lungs were subjected to full histopathology analysis. Again, we confirmed that *Trf1* downregulation was maintained in the generated lung metastasis (Fig5J). Importantly, *Trf1* downregulation resulted in smaller lung metastasis (Fig5K and I), coincidental with increased DNA damage, decreased proliferation, and increased apoptosis compared to the controls (Fig5M–O). These results indicate that even a partial decrease in TRF1 levels of approximately 50% in tumors very significantly impairs lung tumor growth and lung metastasis, arguing that putative small molecule inhibitors of TRF1 could be effective.

A–C The latency (A), volume (B), and weight (C) of subcutaneous tumors generated by control and *Trf1*-downregulated *K-Ras*^*G12V*^-transformed lung cells in athymic mice.

D Representative images of the subcutaneous tumors.

E *Trf1* expression levels measured by qPCR in the injected cell line and in the generated subcutaneous tumors.

F–H Number of Ki67-positive (F), number of γH2AX-positive (G), and number of active caspase-3-positive (H) cells per field in the subcutaneous tumors.

I Representative images of aberrant giant nuclei and anaphase bridges in the *Trf1*-downregulated subcutaneous tumors compared to the normal nuclei of control tumors.

J TRF1 immunofluorescence shows the downregulation of *Trf1* in lung tumors of the mice intravenously injected with control and *Trf1*-downregulated cells.

K Tumor area measured in the lungs of the mice intravenously injected with control and *Trf1*-downregulated cells.

L Representative images of the lungs colonized by control and *Trf1*-downregulated cells, respectively.

M–O Number of Ki67-positive (M), number of γH2AX-positive (N), and number of active caspase-3-positive (O) cells per field in the lung tumors.

P Trf1 expression levels measured by qPCR in the A549 cell line infected either with sh-scrambled or sh-Trf1.

Q Growth of A549-derived tumors. Data information: Error bars represent standard error. The number of mice and tumors analyzed per condition is indicated. *t*-test was used to assess statistical significance.

Next, we tested whether TRF1 depletion had similar effects on human lung cancer cell lines. To this end, we downregulated *Trf1* levels by using shRNAs in a K-Ras-mutated human lung carcinoma cell line, the A549 (ATCC n°; CCL-185), a human lung cancer cell line harboring wild-type p53 (Fig5P). We then injected subcutaneously in immunodeficient mice 150,000 cells either infected with sh-scrambled or *Trf1*-shRNA and followed tumor development. TRF1 downregulation resulted in a markedly delayed tumor onset and growth. Indeed, control cells started to develop tumors 11 days after injection, while the latency of TRF1-depleted cells was of 17 days. Moreover, after 24 days of follow-up, only two out of eight injections with TRF1-downregulated cells were able to generate tumors whose growth were significantly impaired as compared to control cells (Fig5Q and Supplementary Fig S3). Thus, TRF1 downregulation blocks the growth of cell lines derived from already formed lung mouse tumors and has proven efficacy in one human cancer cell line.

### *Trf1* deficiency impairs lung carcinomas independently of telomere length

To demonstrate that the increased apoptosis and proliferation defects observed in *Trf1*-deleted lung carcinomas were not due to shorter telomeres compared to the TRF1-proficient tumors, we determined telomere length by telomere quantitative FISH directly on lung carcinoma sections. Indeed, telomeres were longer in the *Trf1*^Δ/Δ^ *K-Ras*^+*/G12V*^ *p53*^−/−^ carcinomas compared to *Trf1*^+*/*+^ *K-Ras*^+*/G12V*^ *p53*^−/−^ carcinomas (Supplementary Fig S4A–C). As telomere length reflects the proliferative history of a given tissue, the observation that *Trf1*^+*/*+^ *K-Ras*^+*/G12V*^ *p53*^−/−^ carcinomas present shorter telomeres than TRF1-deficient ones is likely to reflect the lower proliferation rate of TRF1-deficient tumors.

### Whole-body *Trf1* depletion allows normal mouse survival and normal tissue function

**Figure 6:**
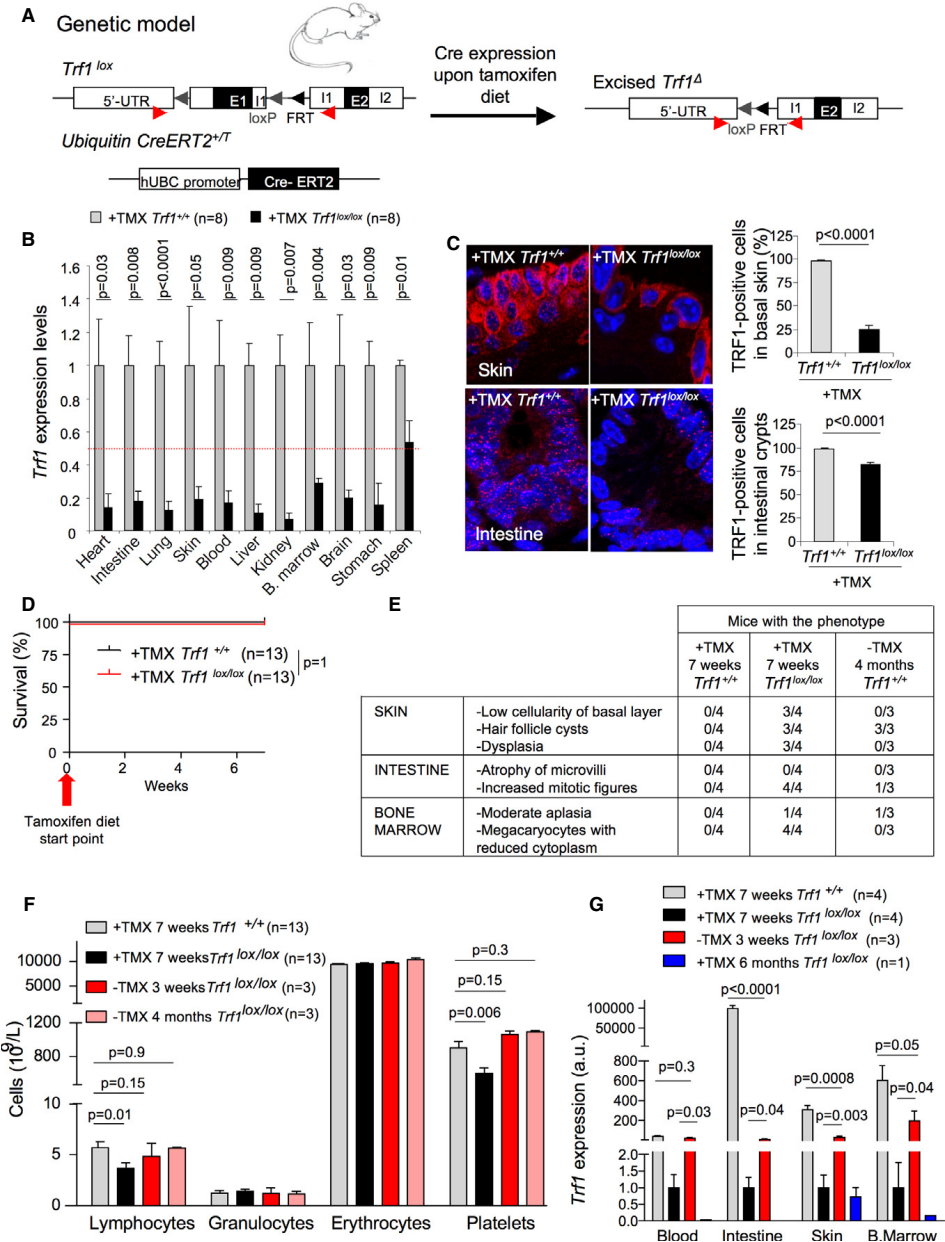
Systemic *Trf1* depletion in healthy tissues does not compromise tissue function nor organism viability

A prerequisite that must be fulfilled by any potential anti-cancer target is that its systemic inhibition in healthy tissues does not compromise organism viability. It has been shown that *Trf1* deletion is deleterious at early points of development (Karlseder *et al*, 2003; Martinez *et al*, 2009). In addition, acute TRF1 removal from bone marrow in 8-week-old mice leads to bone marrow failure (Beier *et al*, 2012). To validate TRF1 as a therapeutic drug target in lung cancer treatment, we set out to analyze the effects of whole-body TRF1 depletion in the context of adult mice and its impact on long-term mouse viability. To this end, we first generated a new mouse model, *Trf1*^*lox/lox*^ *hUBC-CreERT2* mice, in which the expression of CreERT2 is transcriptionally controlled by the ubiquitously and constitutively regulated ubiquitin promoter (Ruzankina *et al*, 2007; Martinez *et al*, 2009) (Fig6A). Then, twelve-week-old *Trf1*^+*/*+^ *hUBC-CreERT2* and *Trf1*^*lox/lox*^ *hUBC-CreERT2* mice were subjected to a tamoxifen-containing diet for 7 weeks in order to induce *Trf1* deletion. After this period of time, a total of eight mice of each genotype were sacrificed to analyze the extent of *Trf1* deletion in a panel of different tissues as well as to perform full histopathological analysis. qPCR analysis showed that *Trf1* had been successfully deleted from heart, intestine, lung, skin, blood, liver, kidney, bone marrow, brain, and stomach (Fig6B). TRF1 immunofluorescence in skin and small intestine sections confirmed partial depletion of TRF1 protein in these tissues (Fig6C). Despite successful TRF1 depletion after 7 weeks in a tamoxifen-containing diet, neither *Trf1*^+*/*+^ nor *Trf1*^Δ/Δ^ showed signs of viability loss or decreased survival (Fig6D). Histopathological analysis of the tissues revealed that the *Trf1*^Δ/Δ^ *hUBC-CreERT2* mice showed alterations in rapidly proliferating tissues consistent with the stem cell compartment being affected (Fig6E). TRF1-depleted basal skin, intestinal crypts, and bone marrow progenitors presented anisocytosis (Supplementary Figs S5 and S6). *Trf1*-deficient basal skin displayed areas with low cellularity and follicular cysts (Fig6E and Supplementary Fig S5A). *Trf1*^Δ/Δ^ intestinal crypts showed an increased number of mitosis, but the microvilli length was normal (Fig6E and Supplementary Fig S5B). *Trf1*^Δ/Δ^ bone marrow presented megakaryocytes with reduced cytoplasm (Fig6E and Supplementary Fig S6A). Blood counts showed a small decrease in the number of platelets and lymphocytes (Fig6F). Of note, only one *Trf1*^Δ/Δ^ *hUBC-CreERT2* mouse showed a moderate bone marrow aplasia (Fig6E and Supplementary Fig S6A). Of note, 6 months on tamoxifen diet did not further decrease *Trf1* expression as compared to the levels observed after 7 weeks and did not aggravate neither pathologies nor mouse viability (Fig6G and data not shown). Importantly, tamoxifen retrieval from the diet resulted in a rescue of pathologies as well as in a recovery of *Trf1* expression levels (Fig6E–G). Indeed, after 3 weeks, placing the mice on standard diet Trf1 levels in blood, intestine, skin, and bone marrow increased 20-, 10-, 30- and 200-fold, respectively, compared to the levels observed in mice on tamoxifen diet. However, recovery of *Trf1* expression did not reach wild-type levels (Fig6G). After 3 weeks and 4 months on standard diet, platelet blood counts were also recovered to wild-type levels (Fig6F). Thus, a partial ubiquitous TRF1 downregulation, although resulting in decreased cellularity in some highly proliferative compartments, such as the bone marrow and the blood, is compatible with mouse viability.

*Trf1*^*lox/lox*^ *hUBC-CreERT2* genetic mouse model.

*Trf1* expression levels in the indicated tissues of wild-type and *Trf1*^*lox/lox*^ *hUBC-CreERT2* mice subjected to a tamoxifen-containing diet for 7 weeks.

Representative images of TRF1 immunofluorescence and quantification of the percentage of TRF1-positive cells in skin and intestine sections of wild-type and *Trf1*^*lox/lox*^ *hUBC-CreERT2* mice subjected to a tamoxifen-containing diet for 7 weeks.

Survival curve of wild-type and *Trf1*^*lox/lox*^ *hUBC-CreERT2* mice subjected to a tamoxifen-containing diet for 7 weeks.

Quantification of the histological alterations observed in tamoxifen-treated *Trf1*^*lox/lox*^ *hUBC-CreERT2* mice and 4 months after tamoxifen retrieval compared to their wild-type counterparts.

Quantification of blood cell populations in wild-type and *Trf1*^*lox/lox*^ *hUBC-CreERT2* mice subjected to a tamoxifen-containing diet for 7 weeks and after 3 weeks and 4 months of tamoxifen retrieval.

*Trf1* expression levels in blood, intestine, skin, and bone marrow of *Trf1*^*lox/lox*^ *hUBC-CreERT2* mice subjected to a tamoxifen-containing diet either for 7 weeks or for 6 months and after 3 weeks tamoxifen retrieval compared to wild-type mice. Data information: Error bars represent standard error. The number of mice analyzed per genotype is indicated in each case. *t*-test was used to assess statistical significance. TMX, tamoxifen.

### Identification of chemical compounds that disrupt TRF1 binding to telomeres

We next set out to identify chemicals that disrupt TRF1 binding to telomeres. To do so, we designed a cell-based system for high-throughput screening. Induced pluripotent stem (iPS) cells derived from eGFP-TRF1 knock-in (KI) mouse embryo fibroblasts (MEFs) were used (Schneider *et al*, 2013). The eGFP-TRF1 protein forms fluorescence foci localizing at the telomere that strongly decrease in intensity in control cells expressing a sh-Trf1 or in cells heterozygous for eGFP-TRF1 expression (*eGFP-Trf1*^+/KI^) showing that this cellular system is an excellent tool to track TRF1 and telomeres *in vivo* (Supplementary Fig S7A). Homozygous version (*eGFP-Trf1*^KI/KI^)—with strong foci intensity—, heterozygous version (*eGFP-Trf1*^+/KI^) and knockdown for TRF1 (*sh-Trf1*)—with low foci intensity—were used to validate this system (Supplementary Fig S7B). The *Z*'-factor coefficient, a statistical parameter that in addition to considering the window in the assay also considers the variance around both the high and low signals in the assay, is commonly used to assess the robustness of high-throughput screening (HTS) assays. The *Z*'-factor was calculated as follows: *Z*'-factor = 1 − 3 × (sp + sn)/¦mp − mn¦, where m: mean fluorescence intensity and s: standard deviation; n: negative control (*sh-Trf1* or *eGFP-Trf1*^+/KI^ heterozygous, minimum signal) and p: positive control (homozygous *eGFP-Trf1*^KI/KI^, maximum signal). A *Z*'-factor value of 0.75, when comparing homozygous (homo) vs. heterozygous (het) or 0.86 comparing homo vs. *sh-Trf1*, confirmed the feasibility of this screening system (Supplementary Fig 7C). We carried out a screening campaign using a small collection of 640 compounds selected as representative of the ETP-CNIO library (Materials and Methods). Screening was performed at 8 h and at 12.5 μM final concentration on *eGFP-Trf1*^KI/KI^ cells. Foci distribution of the homozygous *eGFP-Trf1*^KI/KI^ (control) or *sh-Trf1*, were taken as internal controls. Compounds decreasing percentage of high-intensity foci above 25% of the control were selected as “hit” candidates, for further validations. In addition, the number of nuclei was counted. Compounds significantly affecting cell viability were not considered as positive “hits”.

The screening retrieved a number of positive hits belonging to different chemical classes. The identified hits were newly resynthesized and tested confirming the observed activity. The search for analogues within the ETP-CNIO library and their screening identified additional active compounds. Among the different hits, two of them were selected for further biological investigation attending to their primary activity as “TRF1 inhibitors” and additionally to their potential to be used as tool compounds for *in vivo* validation experiments. The selected hits ETP-47228 and ETP-47037 are included as examples in CNIO international patent applications WO2010119264 and WO2011089400, respectively. The general structures of both compounds are depicted in Supplementary Fig S8A.

We next validated our hits by measuring eGFP-TRF1 fluorescence intensity in *e-GFP-Trf1*^KI/KI^ iPS cells after treatment with DMSO, ETP-47228, or ETP-47037 for 8 h at 10 μM. iPS cells transduced with a *sh-Trf1* were used as a positive control. Both ETP-47228 and ETP-47037 induced a decrease of 31.33 and 27.32%, respectively, of e-GFP-TRF1 levels, as compared to a 57% decrease observed in cells treated with *sh-Trf1* (Supplementary Fig S7D).

To address whether these compounds also decrease endogenous TRF1 levels, we treated wild-type iPS cells (*eGFP-Trf1*^KI/KI^) with 10 μM ETP-47228 for 8 h and quantified TRF1 levels by immunofluorescence. Cells treated with ETP-47228 contained 25% lower levels of TRF1 signal as compared to control cells treated with DMSO (Supplementary Fig S9A). Interestingly, neither the *Trf1* transcriptional levels nor the total TRF1 protein levels were affected by ETP-47228 or ETP-47037 (Supplementary Fig S9B). In analogy to *Trf1* genetic ablation, chemical inhibition of TRF1 foci intensity was accompanied by increased γH2AX DNA damage foci and increased telomeric DNA damage foci (TIFs), together with impairment of cellular proliferation with increasing ETP-47228 concentrations (Supplementary Fig S9C–E).

### Effective chemical Trf1 inhibition in lung adenocarcinoma-derived cells

**Figure 7:**
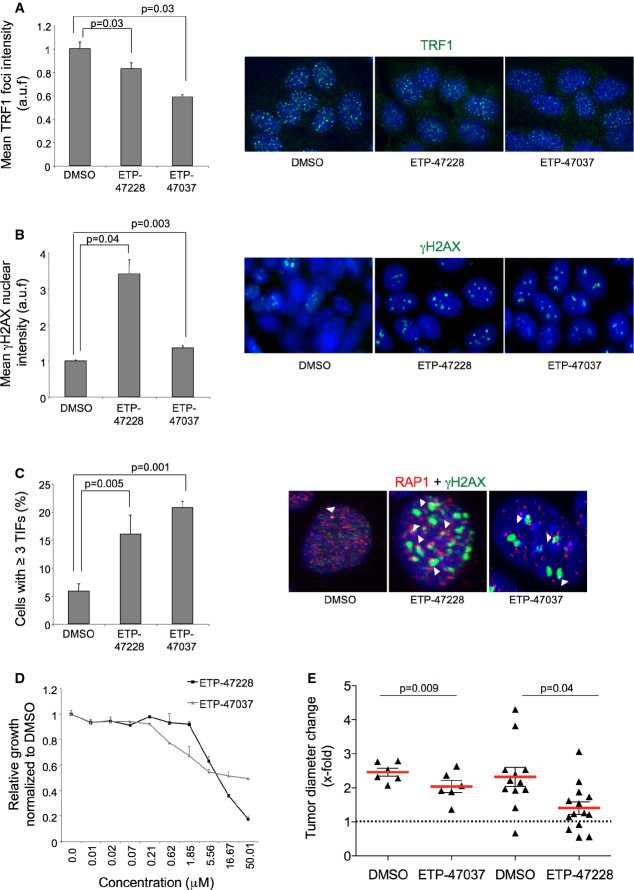
Efficient chemical inhibition of TRF1 telomere binding by compounds ETP-47228 and ETP-47037 in mouse lung adenocarcinoma-derived cells

Next, we treated lung mouse adenocarcinoma cell lines with 10 μM of both ETP-47228 and ETP-47037 for 24 and 48 h and quantified TRF1 levels compared to DMSO-treated cells. Again, treatment of lung cancer cells with both ETP-47228 and ETP-47037 resulted in decreased TRF1 foci levels, increased γH2AX foci, as well as induction of TIFs (Fig7A–C). Similarly, both compounds affected proliferation from concentrations of approximately 5 μM (Fig7D).

Quantification of TRF1 levels by immunofluorescence in lung tumor-derived cell line treated with DMSO, with 10 μM ETP-47228 (24 h), and with 10 μM ETP-47037 (48 h). Representative images are shown to the right.

Quantification of γH2AX levels by immunofluorescence in lung tumor-derived cell line treated with DMSO, with ETP-47228 (24 h), and with ETP-47037 (48 h). Representative images are shown to the right.

Quantification of telomere-induced foci (TIFs) by double immunofluorescence with anti-RAP1 and anti-γH2AX antibodies. Representative images are shown to the right. White arrowheads: colocalization of γH2AX and RAP1.

Effect of different ETP-47228 and ETP-47037 concentrations during 24 h on proliferation in lung tumor-derived cell line relative to the growth of DMSO-treated cells.

Tumor growth quantification in allograft model ETP-47037 or with ETP-47228. Data information: The data represent the mean values of two to three independent experiments (A–D). Error bars represent standard errors. *t*-test was used to assess statistical significance.

Next, we generated allograft mouse models with lung cancer cells pretreated with either DMSO, ETP-47228, or ETP-47037 and followed tumor onset and growth during 12 days. Treatment with both compounds significantly impaired initial tumor development in allograft models (Fig7E).

### *In vivo* treatment with ETP-47037 disrupts TRF1 and effectively impairs lung carcinoma progression

Next, we addressed whether TRF1 chemical inhibitors administered *in vivo* could inhibit the growth of already established *K-Ras*^*G12V*^ lung carcinomas lacking p53. To this end, we selected compound ETP-47037 owing to its *in vivo* pharmacokinetic properties. ETP-47037 pharmacokinetic properties were determined after IV and PO (per os) administration in BALB/c mice at doses of 3.0 and 9 mg/kg of body weight, respectively (Supplementary Fig S8). In particular, ETP-47037 is an orally bioavailable compound with an absorbed fraction *F* = 29.5%. The half-life after IV administration is 0.5 h, and the same parameter in the oral arm extends up to 5.2 h. ETP-47037 is highly stable *in vivo* with a total clearance of 0.65 l/h/kg, which means 12% of the hepatic blood flow for mice. The compound is distributed in tissues, as inferred by a volume of distribution of 0.6 l/kg similar to the total body water content (Supplementary Materials and Methods).

**Figure 8:**
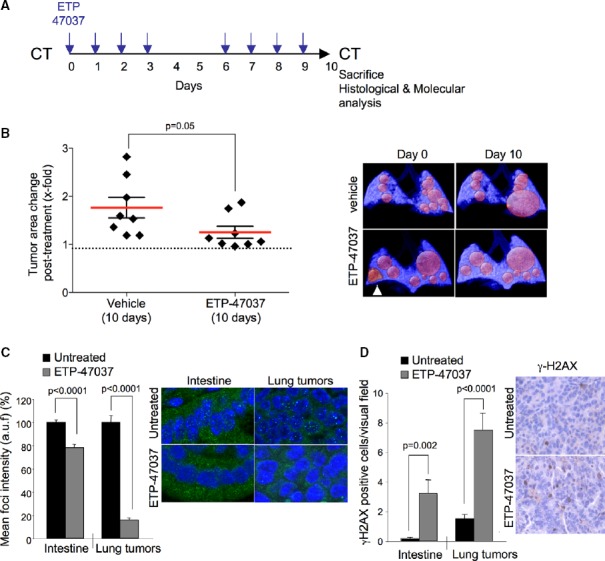
“*In vivo*” treatment with ETP-47037 compound blocks the progression of lung carcinoma

**Figure 9:**
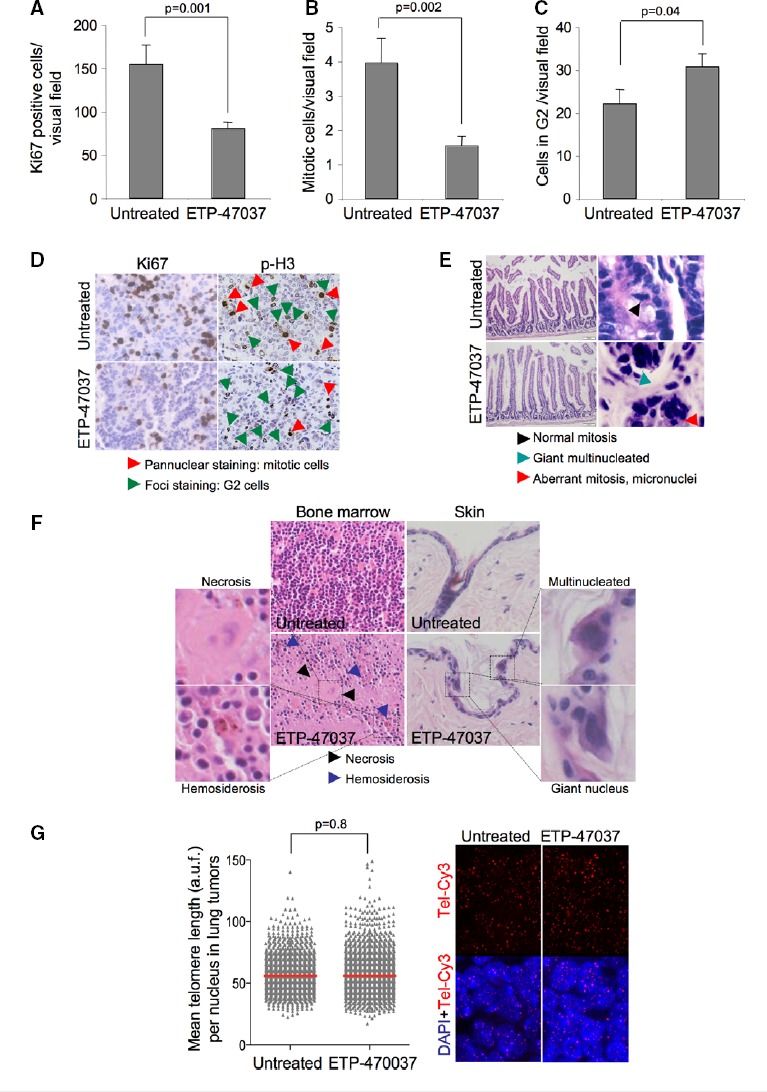
“*In vivo*” treatment with ETP-47037 compound does not compromise tissue viability

Mice with already developed p53-null lung carcinomas were treated by oral gavage during 10 days (8 days in total with a 2-day gap) with 75 mg/kg body weight of ETP-47037. Control mice were similarly treated with vehicle. Previously to the start and at the end of the treatment, mice were subjected to computerized tomography (CT) for quantification of tumor growth area. Strikingly, 10 days of treatment with this compound effectively impaired the progression of already formed lung carcinomas compared to the group treated with vehicle (Fig8A and B). Of note, one of the tumors detected before the treatment was located in a highly inflammatory region, and for this reason, we could not accurately determine its size before the treatment (white arrowhead in Fig8B). TRF1 foci levels were significantly decreased in tumor samples as well as in intestines of mice treated with ETP-47037 compared to the vehicle (Fig8C). Decrease in TRF1 foci signal was accompanied with a significant increase in γH2AX-positive cells in both intestines and lung tumors (Fig8D). In addition, ET-47037-treated tumors showed a significant decrease in proliferating and mitotic cells and increase in the number of G2-arrested cells (Fig9A–D). Importantly, during the treatment, the mice showed a normal viability and did not show any signs of sickness. Histopathological analysis of the intestine did not reveal any apparent lesion although we saw increased aberrant mitotic figures and nuclei characteristic of TRF1 inhibition (Fig9E). In bone marrow, moderate aplasia, necrotic cells, and hemosiderosis were observed. In the skin, multinucleated cells and giant nuclei were detected (Fig9F), a hallmark of TRF1 targeting. Of note, ETP-47037 did not induce changes in telomere length in treated tumors as compared to untreated ones (Fig9G). These findings indicate that TRF1 inhibition can be achieved *in vivo* using chemical compounds and that there is a therapeutic window for targeting TRF1 in cancer that merits further work.

Schematic representation of the ETP-47037 treatment protocol. Mice with already developed lung carcinomas were subjected to computerized tomography (CT) measurements before the start of the treatment. ETP-47037 was given at a dose of 75 mg/kg body weight by oral gavage 8 days out of the ten that the experiment lasted as indicated. At the end of the treatment, a CT was performed for quantification of tumor area and mice were sacrificed for further histological and molecular analysis. Control mice were similarly treated with vehicle.

Quantification of tumor growth relative to initial tumor size. Representative CT images are shown to the right. The white arrowhead indicates a tumor within a highly inflammatory region.

Quantification of TRF1 levels by immunofluorescence in intestine and lung tumors samples of mice treated with vehicle or with ETP-47037 for 10 days. Representative images are shown to the right (*n* = 4).

Number of cells showing γH2AX foci in intestine and lung tumors samples of mice treated with vehicle or with ETP-47037 for 10 days. Representative images are shown to the right (*n* = 4). Data information: The data represent the mean values obtained from three mice in each group. Error bars represent standard errors. *t*-test was used to assess statistical significance.

A–C Quantification of the number of (A) Ki67-, (B) pan-nuclear p-H3 pattern-, and (C) foci p-H3 pattern-positive cells in untreated and ETP-470037-treated lung carcinomas. The data represent the mean values obtained for three mice in each group. Error bars represent standard errors.

D Representative Ki67 and p-H3 images.

E Representative H&E images of intestine samples corresponding to untreated and ETP-47037-treated animals. High-magnification images are shown to the right indicating the presence of normal mitosis, giant multinucleated and aberrant mitotic figures.

F Representative H&E images of bone marrow and skin samples corresponding to untreated and ETP-47037-treated animals. High-magnification images are shown indicating the presence of necrosis, hemosiderosis, multinucleated cells, and giant nuclei. Bone marrow showed moderated aplasia.

G Telomere length in untreated and ETP-47037-treated lung tumor samples. Representative images are shown to the right. Data information: *t*-test was used to assess statistical significance.

## Discussion

Aberrant telomerase activation is a common feature of human cancers, where it allows the growth of malignant cells by ensuring maintenance of a minimal functional telomere length that permits cell division (Kim *et al*, 1994; Hahn *et al*, 1999; Gonzalez-Suarez *et al*, 2000). Indeed, mutations in the telomerase gene or its regulatory regions have been found associated with many different types of cancer (McKay *et al*, 2008; Wang *et al*, 2008; Rafnar *et al*, 2009; Shete *et al*, 2009; Petersen *et al*, 2010; Melin *et al*, 2012; Bojesen *et al*, 2013; Garcia-Closas *et al*, 2013; Horn *et al*, 2013; Huang *et al*, 2013). To date, targeting of telomeres in human cancer has been mainly via targeting telomerase activity, typically through direct small molecule inhibitors of the enzyme activity (Brennan *et al*, 2010; Joseph *et al*, 2010), or through immunotherapy-based approaches (Brunsvig *et al*, 2006; Suso *et al*, 2011). Telomeric repeats can also form DNA higher order structures known as G-quartets, and molecules that stabilize G-quartets have also been proposed to inhibit telomerase-mediated telomere elongation in cancer (Sun *et al*, 1997; Shin-ya *et al*, 2001; Huang *et al*, 2008). A predicted shortcoming of therapeutic strategies based on telomerase inhibition to treat cancer is that they will be effective only when telomeres shorten below a minimum length. Indeed, telomerase activity is dispensable for transformation of cells with long telomeres (Seger *et al*, 2002), and studies with telomerase inhibitors indicate that they are effective preferentially in cells with short telomeres (Hahn *et al*, 1999; Herbert *et al*, 1999; Wang *et al*, 2004; Brennan *et al*, 2010; Wu *et al*, 2012; reviewed in Buseman *et al*, 2012). In line with this, telomerase abrogation in the context of cancer-prone mouse models, including the *K-Ras*^+*/G12D*^ lung tumorigenesis mouse model, only showed anti-tumorigenic activity after several mouse generations in the absence of telomerase when telomeres reached a critically short length (Chin *et al*, 1999; Greenberg *et al*, 1999; Gonzalez-Suarez *et al*, 2000; Perera *et al*, 2008). Moreover, these anti-tumorigenic effects of short telomeres owing to telomerase deficiency are abrogated in the absence of p53 (Chin *et al*, 1999; Greenberg *et al*, 1999).

In contrast to telomerase inhibition, telomere uncapping has been shown to cause rapid induction of cell death and/or senescence in a manner that is independent of telomerase activity and telomere length (Karlseder *et al*, 1999; Smogorzewska & de Lange, 2002; Martinez *et al*, 2009). Owing to the fact that telomere uncapping can be achieved independently of telomere length, it emerges as a more universal way to rapidly impair the growth of dividing cells. Indeed, in our experimental system, *Trf1* abrogation results in a dramatic reduction in the number and the size of malignant lung carcinoma lesions, even in the absence of p53, already in the first mouse generation and in the absence of telomere shortening, indicating that *Trf1* deficiency severely impairs cancer progression in the context of oncogenic *K-Ras*. As a consequence of this, all the *Trf1*^Δ/Δ^ *K-Ras*^+*/G12V*^ *p53*^−/−^ mice survived until the end point of the experiment (24 weeks post-infection), while only 50% of the *Trf1*^+*/*+^ *K-Ras*^+*/G12V*^ *p53*^−/−^ mice survived the same period. These findings indicate that *Trf1* deficiency impairs the development of *K-Ras*-induced lung carcinomas. Of note, this represents the first time that effective impairment of *K-Ras*^+*/G12V*^ *p53*^−/−^ carcinomas is achieved, as genetic abrogation of other therapeutic pathways did not impair tumor growth in the absence of p53 (Navas *et al*, 2012). Furthermore, here we show that downregulation of *Trf1* can also block the growth and metastatic potential of both mouse and human lung cancer cell lines derived from already established *K-Ras*-induced lung carcinomas by using xenograft models.

We find that the mechanisms through which *Trf1* deletion impairs cancer progression are related to its previously described roles in telomere capping, telomere replication, and mitosis (Martinez *et al*, 2009; Sfeir *et al*, 2009). In this regard, we show that *Trf1* deficiency results in a high burden of telomeric DNA damage, genetic instability, proliferation defects, apoptosis, and mitotic catastrophe.

Importantly, we demonstrate here that a long-term systemic depletion of TRF1 in healthy adult tissues does not compromise organism viability, although we observed decreased cellularity in some highly proliferative compartments, such as the hematopoietic compartment and blood, which were recovered upon tamoxifen removal. Together, these findings suggest a therapeutic window for TRF1 inhibition in cancer.

Inspired by the above notion, we have identified compounds that disrupt TRF1 binding to telomeres illustrating the feasibility of chemically targeting shelterin proteins. Furthermore, we have shown that “*in vivo*” treatment of already established lung adenocarcinomas with one of the identified compounds, ETP-47037, results in decreased TRF1 signal *in vivo* and the impairment of tumor progression in the absence of decreased mouse viability.

In summary, the results described here are proof of concept that TRF1 abrogation is an effective therapeutic strategy to block the growth of aggressive lung carcinomas independently of telomere length and p53 status and that it is possible to achieve this by small molecules that are able to target TRF1 *in vivo*. Finally, as this strategy relies on a universal mechanism, namely induction of telomere uncapping, we speculate that it could be applied in many other cancer types.

## Materials and Methods

### Mice

*K-Ras*^+*/LSLG12Vgeo*^ (Guerra *et al*, 2003), and *Trf1*^*lox/lox*^ (Martinez *et al*, 2009), *p53*^−/−^ (Jackson Labs, http://jaxmice.jax.org/strain/002101.html) strains were crossed to obtain *K-Ras*^+*/LSLG12Vgeo*^ *Trf1*^*lox/lox*^ *p53*^−/−^ mice. To generate *Trf1*^*lox/lox*^ *hUBC-CreERT2* mice, we crossed our *Trf1*^*lox/lox*^ (Martinez *et al*, 2009) with a mouse strain that carries a ubiquitously expressed, tamoxifen-activated recombinase, *hUBC-CreERT2* mice (Ruzankina *et al*, 2007). *Trf1*^*lox/lox*^ *hUBC-CreERT2* mice were fed *ad libitum* for 7 weeks with tamoxifen-containing diet (Tekland CRD Tam^400^/CreER). For allograft experiments, 7-week-old athymic nude females were obtained from Harlan. All mice were maintained at the Spanish National Cancer Center under specific pathogen-free conditions in accordance with the recommendations of the Federation of European Laboratory Animal Science Associations (FELASA). All animal experiments were approved by the Ethical Committee and performed in accordance with the guidelines stated in the International Guiding Principles for Biomedical Research Involving Animals, developed by the Council for International Organizations of Medical Sciences (CIOMS).

### Adenovirus intratracheal infection

Eight- to ten-week-old mice were treated once with intratracheal adeno-Cre (Gene Vector Core, University of Iowa, 1 × 10^10^ pfu/ml) instillation with 1 × 10^8^ PFU/mouse of virus after anesthesia by intraperitoneal injection of ketamine–medetomidine (Domitor, 1 mg/ml; Orion Corporation). To wake up the mice after the instillation, they were injected with 0.05 mg of atipamezole (Antisedan, 5 mg/ml; Orion Corporation).

### *In vivo* imaging by computed tomography (CT) and positron emission tomography (PET)

Nine weeks after inoculation, an *in vivo* follow-up of tumor growth was achieved by six computed tomographies (CT) every 15 days. PET was performed 22^nd^ week post-inoculation, and the mice were sacrificed (24^th^ week post-inoculation). CT and PET analyses were performed as previously described (Ambrogio *et al*, 2014). For PET quantification, tumor regions of interest (ROIs) were selected in the PET-CT overlapped image. In these ROIs, the standardized ^18^FDG-glucose uptake value (SUV) was calculated using the following formula: SUV = tumor FDG concentration (MBq)/(injected dose/body weight).

### Telomere length analyses on tissue sections

Quantitative telomere fluorescence in situ hybridization (Q-FISH) directly on tumor sections was performed as previously described (Flores *et al*, 2008) and analyzed by Definiens software.

### Chemical library

The Experimental Therapeutics Program at CNIO, ETP-CNIO, owns a chemical library of about 50,000 single compounds built as a result of the consolidation of several sub-libraries selected attending to different criteria such as chemical diversity, kinase-targeted focus, potential to disrupt protein–protein interactions, and the presence of low molecular weight compounds to facilitate fragment-based drug discovery. The drug-likeness of the whole library was also ensured by the application of filters such as “rule of five” (Lipinski, 2004). The compounds were selected from commercial origin as well as from internally newly designed and synthesized chemical matter. Representative libraries of the whole 50K library with smaller sizes were defined after clustering, based on similarity analysis, and selection of representative compounds from each cluster. A 640-compound library, subject of the currently reported screening campaign, is the minimum size set of compounds representing the chemical ETP-CNIO collection.

### Screening for identification of TRF1 inhibitors

We tested the CNIO-640 library previously described. iPS cells expressing eGFP-TRF1 were seeded in 0.1% gelatin-pretreated cell-carrier black 384-well microplates (Perkin Elmer) at a density of 1.25 × 10^4^ cells per well 24 h before adding the compounds. Compounds were weighed out and diluted in dimethyl sulfoxide (DMSO) to a final concentration of 10 mM (mother plate). From here, an intermediate dilution plate was prepared. The appropriate volume (μl) of each compound solution was added automatically (Beckman FX 96 tip) from the intermediated plate to the media of plated cells to get a 12.5-μM final concentration for each compound assayed in duplicate. Cell viability was previously tested in a dose curve with increasing concentrations of DMSO. After 8-h incubation, cells were fixed in 4% paraformaldehyde in phosphate-buffered saline (PBS) for 15 min at room temperature and washed three times with PBS. Those compounds that killed cells at 12.5 μM at 8 h were not considered as positive hits.

For quantitative measurement of eGFP-TRF1 foci levels, pictures of fixed cells were automatically acquired from each well by the Opera High Content Screening (HCS) system (Perkin Elmer). Sixty images of random fields per well, with a 40× magnification lens, were taken under non-saturating conditions. At least 1 × 10^3^ cells were analyzed for each well. Briefly, images were segmented using the DAPI staining to generate masks matching cell nuclei from which eGFP-TRF1 foci were analyzed. SPSS software was used for statistical analysis as follows: Within each plate, the eGFP-TRF1 intensities of control *eGFP-Trf1*^KI/KI^ cells were distributed by quartiles (Q). First-quartile distribution (Q1) was taken as threshold to distinguish low- or high-intensity eGFP-TRF1 foci. Percentage of low vs. high GFP-TRF1 levels was normalized using the average of negative and positive controls as minimum and maximum reference levels. The number obtained was taken as relative TRF1 inhibition for each compound.

**The paper explained**

**Problem**

Unlimited cell division in cancer requires activation of mechanisms that ensure maintenance of telomere length. Targeting of telomeres in human cancer has been approached via targeting telomerase activity. A caveat of therapeutic strategies based on telomerase inhibition to treat cancer is that they will be effective only when telomeres shorten below a minimum length. We have addressed whether induction of telomere dysfunction independently of telomere length by targeting a shelterin component could be applied as a more universal way to rapidly impair the growth of dividing cells.

**Results**

We demonstrate that acute telomere uncapping owing to inhibition of the TRF1 shelterin component has therapeutic activity in blocking the growth of *p53*-deficient *K-Ras*-induced lung tumors by inducing DNA damage at telomeres. This anti-tumorigenic activity of TRF1 inhibition is independent of telomere length. In parallel, we show that whole-body partial TRF1 depletion, although resulting in moderate loss of cellularity in the bone marrow in few *Trf1*-deleted mice, did not impair organismal viability and survival. Importantly, we identify small molecules that disrupt TRF1 binding *in vivo*, and that effectively block the growth of already established *p53*-deficient *K-Ras*-induced lung carcinomas through induction of DNA damage and cell arrest, again in the absence of deleterious effects in mouse survival or viability.

**Impact**

This represents the first demonstration that targeting the TRF1 shelterin component may represent a novel therapeutic approach for cancer treatment.

### *In vivo* treatment with compound ETP-47037

*K-Ras*^+*/LSLG12Vgeo*^ *Trf1*^*lox/lox*^ *p53*^−/−^ tumors were induced by intratracheal adeno-Cre instillation as described above. Once the lung tumors developed, mice were daily dosed orally with 75 mg/kg of ETP-47037 formulated in 10% N-methyl-pyrrolidone and 90% polyethylene-glycol 300 for 10 days and 2 days of resting. The reduction in number and size of the tumors was analyzed by computed tomography (CT).

## Supporting Information

Supplementary Information

Review Process File
