# Supplementary material for: Therapeutic inhibition of TRF1 impairs the growth of p53-deficient K-RasG12V-induced lung cancer by induction of telomeric DNA damage
Source: EMBO Mol Med. 2015 May 13;7(7):930–49. doi: 10.15252/emmm.201404497 (PMC4520658; doi:10.15252/emmm.201404497)

Supplemental Information

Supplemental Materials and Methods

Cell culture assays

MEFs were isolated from E13.5 embryos and expanded according to standard protocols, in DMEM supplemented with 2% fetal calf serum. MEFs (1-3 × 10^6) were plated and infected 3 times with pBabeCre viral supernatant supplemented with 4 μg/mL polybrene. MEFs were then selected with 2 μg/mL puromycin for 3 days. For all the assays performed, they were kept on DMEM with puromycin supplemented with 2% fetal calf serum. *Trf1* excision was monitored by PCR as previously described (Martinez et al, 2009). For proliferation assays, 5 X 10^4 cells were plated on six-well plates, with duplicates. Their growth rate was determined using an automatic cell counter (Millipore Scepter Automatic Cell Counter), on days 2, 3, 4, 5 and 7 post-plating. For colony formation assays, 5000 cells were seeded on 10-cm plates, with duplicates. After 2 weeks, cells were fixed and stained with GIEMSA. Colonies were counted and measured. β-galactosidase senescence-associated activity on day 7 was detected using a commercial kit (Cell Signalling). For proliferation assay CellTiter 96®AQueousOne Solution Cell Proliferation Assay (G3582, Promega) was used according to the manufactures protocol

For allograft and xenograft experiments, *K-Ras*$^{\Delta/LG12Vgeo}$ *p53*$^{-/-}$ tumor-derived cell line (M. Barbacid's gift) and the A549 human carcinoma cell line (ATCC nº; CCL-185) were infected with a shRNA against *Trf1* (pLKO.1-puro-Trf1 shRNA, Sigma-Aldrich) as described above. Trf1 expression was quantified by qPCR using the following primers: TRF1 FB (5' TCT AAG GAT AGG CCA GAT GCC A 3') and TRF1 RB (5'CTG AAA TCT GAT GGA GCA CGT C 3'. Actin was used as a house keeping gene. We determined the relative expression of *Trf1* in each sample by calculating the 2*ΔCT* value. For each sample, 2*ΔCT* was normalized to the control 2*ΔCT* mean.

Western blotting

Western blot was performed on MEFs pellets from day 7 following standard procedures. The antibody used was raised against active Caspase 3 (Asp 175, Cell Signaling) and anti-Trf1 (ab10579, Abcam). For iPS cells, 20µg of nuclear extracts of untreated control (DMSO) or treated (ETP-47228 and ETP-47037) were resolved in 4–12% SDS/PAGE gels (NuPAGE Invitrogen) and transferred to nitrocellulose membranes. Blots were incubated with the primary antibodies anti-TRF1 (raised at the Monoclonal Antibodies Unit at CNIO, against full-length mouse TRF1 protein) or anti-SMC1 (Bethyl Laboratories #A300-055A) used as loading control.

Allograft and xenograft experiments

For allograft experiments 150,000 and 50,000 control and *Trf1*-dowregulated *K-Ras*$^{\Delta/LG12Vgeo}$ *p53*$^{-/-}$ tumor-derived cells were intravenously or subcutaneously injected in athymic mice, respectively. For xenograft experiments, 150 000 cells were injected subcutaneously. Three weeks after injection mice were sacrificed. Lungs were fixed and included in paraffin for tumoral area quantification. Subcutaneously grown tumors were measured, weighted and included in paraffin blocks. Tumor volume was determined by the following equation: $V = (4/3) * (a/2) * (b/2) * (c/2) * \Pi$, where *a*, *b* and *c* are tumor length, width and high, respectively. Allograft experiments were also carried out with 50,000 *Ras*$^{\Delta/LG12Vgeo}$ *p53*$^{-/-}$ tumor-derived cells treated with 10 µM of ETP-47037 and 10 µM of ETP-47228 during 48 h, and then subcutaneously injected in athymic mice.

Histopathology, Immunohistochemistry and immunofluorescence techniques

For quantification and classification of tumor lesions, 4/5 of lung lobes were fixed in 10% buffered formalin (Sigma) and embedded in paraffin. The remaining 1/5 of lung lobe was processed for whole-mount X-Gal staining to detect β-Geo, as a surrogate marker for *K-Ras*^{G12V} expression (Guerra et al, 2003). Stained tissues were

embedded in paraffin, serially sectioned, and tumors counted and analyzed by a pathologist. Following the classical histopathology criteria for the diagnosis of malignancy (local/vascular invasion and metastasis), the pathologist classified the tumors into adenomas (benign) and adenocarcinomas (malignant). Due to the high cellular atypia (anaplasia) observed in the *Trf1*$^{\Delta/\Delta}$ *K-Ras*$^{+/G12V}$ *p53*$^{-/-}$ tumors, pure lepidic growth lacking invasion, regardless of the cellular atypia was classified as benign. Three sections of each lung were digitally scanned (Mirax Panoramic Scanner, 3DHistech) for lesion size measure with Panoramic Viewer software.

Antibodies used for immunostaining included those raised against: phospho-histone H3 Ser 10 (06-570, Millipore), phospho-H2AX Ser 139 (05-636, Millipore), Ki67 (SP&, Master diagnostica), β-galactosidase (generated at CNIO Monoclonal Antibodies unit, kindly provided by G.Roncador) and active Caspase 3 (AF835, R&D Systems). For immunofluorescence, the antibodies used were anti-Rap1 (BL735, Bethyl), a rabbit polyclonal anti-TRF1 (homemade) and phospho-H2AX Ser 139 (05-636, Millipore). RAP1 & γH2AX double immunofluorescence and TRF1 inmunofluorescence images were obtained using a confocal ultraspectral microscope (Leica TCS-SP5). Analysis of fluorescence intensities were analyzed with Definiens software.

Pharmacokinetic study of ETP-47037

Female BABL/c mice, 10 weeks old were used (n=3 per time point) for the pharmacokinetic studies of ETP-47037. The compound was formulated in 10 %N-methyl-pyrrolidone and 90 % polyethylene-glycol 300 for oral administrations and in 10 %N-methyl-pyrrolidone, 50 % polyethyleneglycol 300 and 40 % glucose at 5 % in water for intravenous (i.v.) injections. Plasma samples were collected following a single i.v. or oral administration of 3 mg/kg at 0.08, 0.25, 0.5, 1, 4, 8 hours. The analysis of ETP-47037 from plasma was achieved by solid phase extraction followed by high performance liquid chromatography/ tandem mass spectrometry (Agilent 1100, Applied Biosystems API2000) analysis. The amount of inhibitor and the internal standard in

each mouse plasma sample were quantified based on calibration curves generated using standards of known concentrations of compound. This assay method was sufficiently accurate for the quantification of ETP-47037 with a limit of detection (LOD) and a low limit of quantification (LLOQ) of 5 ng/mL. Pharmacokinetic parameters were estimated using WinNonlin Version 5.2 software (Pharsight Corp., CA), by fitting both the experimental i.v and oral data to a bicompartimental model.

Supplemental References

Guerra C, Mijimolle N, Dhawahir A, Dubus P, Barradas M, Serrano M, Campuzano V, Barbacid M (2003) Tumor induction by an endogenous K-ras oncogene is highly dependent on cellular context. *Cancer Cell* 4(2): 111-120

Martinez P, Thanasoula M, Munoz P, Liao C, Tejera A, McNees C, Flores JM, Fernandez-Capetillo O, Tarsounas M, Blasco MA (2009) Increased telomere fragility and fusions resulting from TRF1 deficiency lead to degenerative pathologies and increased cancer in mice. *Genes Dev* 23(17): 2060-2075

Supplemental Figure Legends

Supplementary Figure 1. *Trf1* deficiency induces senescence and impairs cell proliferation and immortalization *in vitro.* **(A)** MEFs growth curve of the indicated genotype. **(B)** Percentage of senescent MEFs at day 7 post plating determined by β-galactosidase senescence-associated staining. **(C)** Caspase 3 activation quantified by western blot in MEFs of the indicated genotypes. Excised activated caspase 3 appears as a double band of 17 and 19 kDa. **(D)** Colony formation assay performed in MEFs of the indicated genotype. Number and size of the colonies are represented. **(E)** Representative image of the colony formation assay. n,number of independent MEFs used per genotype. Error bars represent standard error. T-test was used for statistical significance

Supplementary Figure 2. *Trf1* deficiency impairs *K-Ras*–mediated lung cancer progression.(A-B) Representative images of $Trf1^{+/+}$ $K\text{-}Ras^{+/G12}$ $p53^{-/-}$ and $Trf1^{\Delta/\Delta}$ $K\text{-}Ras^{+/G12}$ $p53^{-/-}$ lungs at death point. Arrow heads mark carcinomas **(D)** Quantification of the number and size of $Trf1^{+/+}$ $K\text{-}Ras^{+/G12}$ $p53^{-/-}$ and $Trf1^{\Delta/\Delta}$ $K\text{-}Ras^{+/G12}$ $p53^{-/-}$ carcinomas at death point. Chi-squared was used for statistical significance.

Supplementary figure 3. Tumor growth slope corresponding to Figure 5q.

Supplementary figure 4. *Trf1*-deficient carcinomas show a shorter proliferative history. **(A)** Telomere length in the healthy pulmonary tissue of the indicated genotypes quantified by Q-FISH analysis. The number of mice per genotype is indicated in each case. **(B)** Telomere length in the carcinomas of the indicated genotypes quantified by Q-FISH analysis. The number of mice and carcinomas analyzed per genotype is indicated in each case. **(C)** Percentage of decrease in telomere length from healthy tissue to carcinomas, based on the Q-FISH data. Error

bars represent standard error. The number of mice and carcinomas analyzed per genotype is indicated in each case. T-test was used for statistical significance.

Supplementary figure 5. *Trf1*-deficient skin and intestine show minor histological alterations and no change in microvilli length. (A) Representative images of tamoxifen treated wild type and *Trf1$^{lox/lox}$ hUBC-CreERT2* skin. A hair follicle cyst is indicated by an arrow. Anysocaryosis is indicated by yellow arrow heads. **(B)** Representative images of tamoxifen treated wild type and *Trf1$^{lox/lox}$ hUBC-CreERT2* intestine. Giant nuclei and mitotic figures are indicated by yellow and black arrow heads, respectively. Quantification of microvilli length in tamoxifen treated wild type and *Trf1$^{lox/lox}$ hUBC-CreERT2* intestine. Error bars represent standard error. The number of mice analyzed per genotype is indicated in each case. T-test was used for statistical analysis.

Supplementary figure 6. *Trf1*-deficient bone marrow histological analysis. (A) Representative images of tamoxifen treated wild type and *Trf1$^{lox/lox}$ hUBC-CreERT2* bone marrow. The Trf1-depleted bone marrow shows some areas of moderate aplasia and megakaryocytes with small cytoplasm (black arrow heads).

Supplementary Figure 7: (**A)** Cell based platform for identification of Trf1 inhibitors, High-Throughput-Screening (HTS). eGFP-Trf1ki/ki iPS cells and cells knock-down for Trf1(sh-Trf1) were taken as positive and negative controls, respectively. Representative images of eGFP-Trf1 foci (green) as detected by confocal microscopy using the OPERA system in eGFP-Trf1ki/ki iPS cells treated with positive or negative controls. Nuclei are stained with DAPI (blue). (**B)** For quantification, the eGFP intensities of control *eGFP-Trf1$^{ki/ki}$* cells were distributed by quartiles (Q). First quartile distribution (Q1) was taken as threshold to distinguish low or high intensity eGFP-Trf1 foci. Percentage of low vs. High foci intensity was calculated. Compounds significantly

decreasing percentage of high intensity foci were considered as positives hits and were taken for further validation. (**C)** The Z'-factor coefficient, a statistical parameter that in addition to consider the window in the assay also considers the variance around both the high and low signals in the assay , it is commonly used to assess the robustness of high throughput screening (HTS) assays. The z' factor was calculated as follow: Z'-factor=1-3X(sp+sn)/|mp-mn|,(1) m: mean fluorescence intensity and s: standard deviation; n:negative control (sh-Trf1 or eGFP-Trf1+/ki heterozygous, minimum signal) and p:positive (homozygous eGFP-Trf1, maximum signal). Z'-Factors of (Homo vs. Het) and (Homovs. sh-TRF1) iPS ≥ 0.5 confirmed feasibility of the screening. (**D)** Quantification of GFP-Trf1 levels in *eGFP-Trf1*$^{ki/ki}$ iPS cells treated with DMSO (positive control), harboring *sh-Trf1*, and treated with two positives hit compounds found in our screening, ETP-47228 and ETP-47037. The data represents mean values of three independents experiments. Representative images are shown to the right. Error bars represent standard errors. T-test was used for statistical significance.

Supplementary figure 8: (**A**) General structures of hits identified in the screening. (**B**) Pharmacokinetic profile of ETP-47037. ETP 47037 plasma concentration (Cp) evolution vs time after intravenous (IV) (3 mg/kg) and oral (PO) (9 mg/Kg) administration. (**C**) ETP 47037 plasma concentrations after IV and PO administration. (**D**) ETP 47037 pharmacokinetic parameters estimated by fitting the experimental data to a bicompartmental model using Winnonlin software for pharmacokinetic analysis. The parameters are: Bioavailability (F); Maximum plasma concentration (Cmax); time for maximum plasma concentration (Tmax); area under the curve (AUC); plasma half life of the product (t ½); plasma clearance (Cl); volume of distribution (Vd).

Supplementary Figure 9: (**A)** Quantification of Trf1 levels by immunofluorescence in iPS cells treated for 8h with DMSO or with 10μM ETP-47228. Representative images are shown to the right. (**B)** Quantification of *Trf1* transcriptional levels by Q-PCR and

Trf1 protein levels by Western Blot in iPS cells treated with either DMSO, with10μM ETP-47228 or with10μM ETP-47037. (**C**) Quantification of γH2AX levels by immunofluorescence in iPS cells treated for 8 h with DMSO or with ETP-47228. Representative images are shown to the right. (**D)** Quantification of telomere induced foci (TIFs) by double immunofluorescence with anti-RAP1 and anti-γH2AX antibodies. Representative images are shown to the right. White arrow heads indicate TIFs. (**E)** Effect on proliferation in iPS cells of different ETP-47228 concentrations during 24 h relative to the growth of DMSO-treated cells. The data represents the mean values for two independents experiments. Error bars represent standard errors. T-test was used for statistical significance.

Supplementary Figure 1

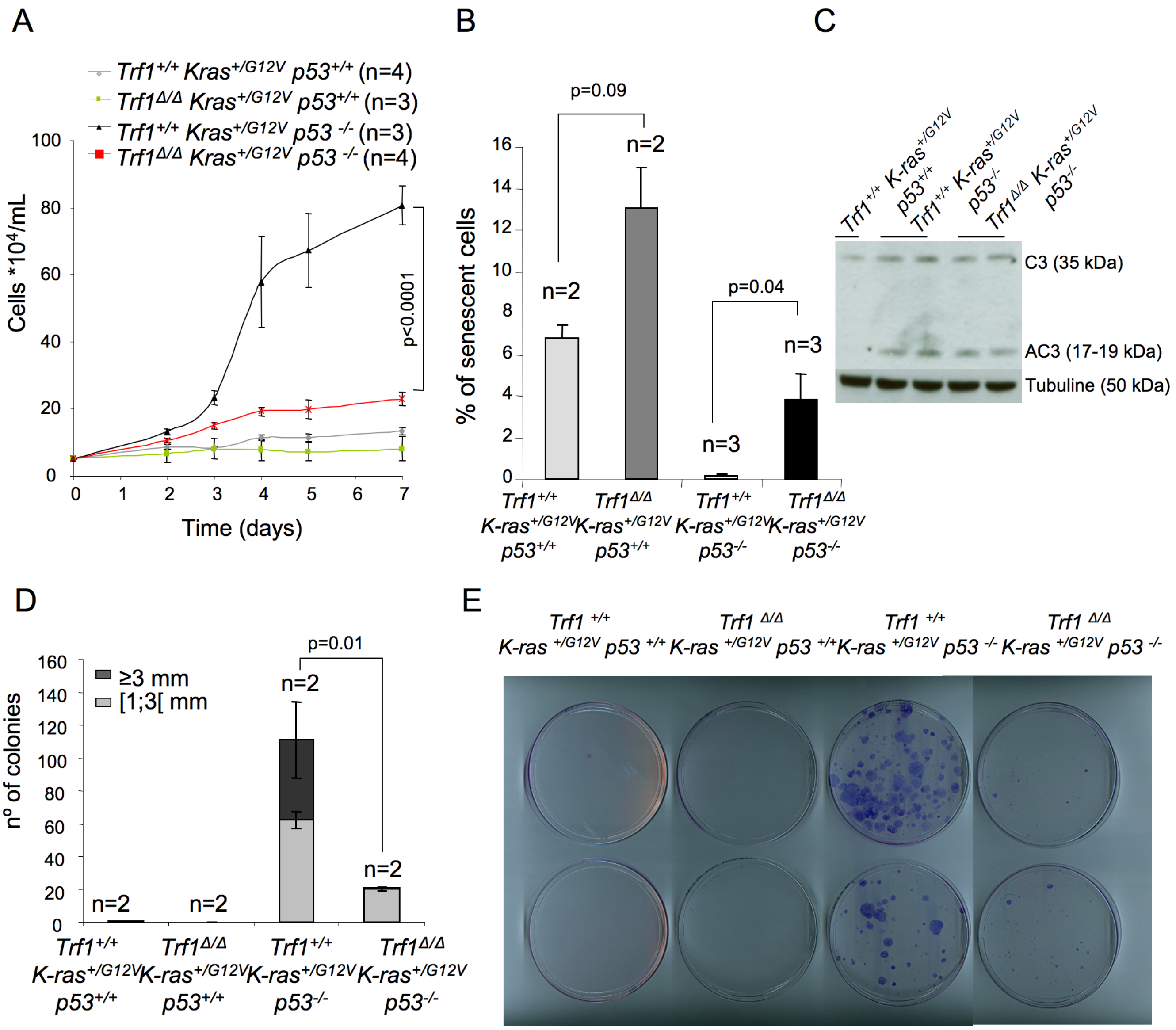

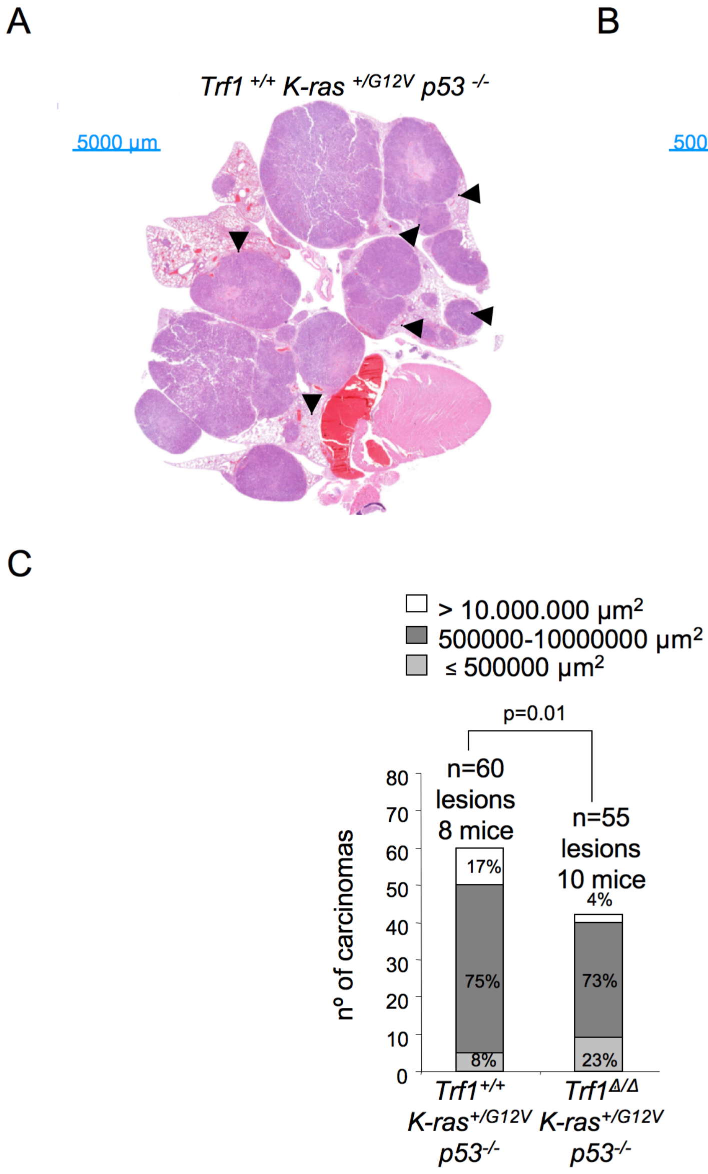
A
Trf1 +/+ K-ras +/G12V p53 -/-
5000 µm
B
Trf1 Δ/Δ K-ras +/G12V p53 -/-
5000 µm
C
> 10.000.000 µm2
500000-10000000 µm2
≤ 500000 µm2
p=0.01
n=60 lesions 8 mice
n=55 lesions 10 mice
17%
75%
8%
4%
73%
23%
nº of carcinomas
0
10
20
30
40
50
60
70
80
Trf1+/+ K-ras+/G12V p53-/-
Trf1Δ/Δ K-ras+/G12V p53-/-

Supplementary Figure 3

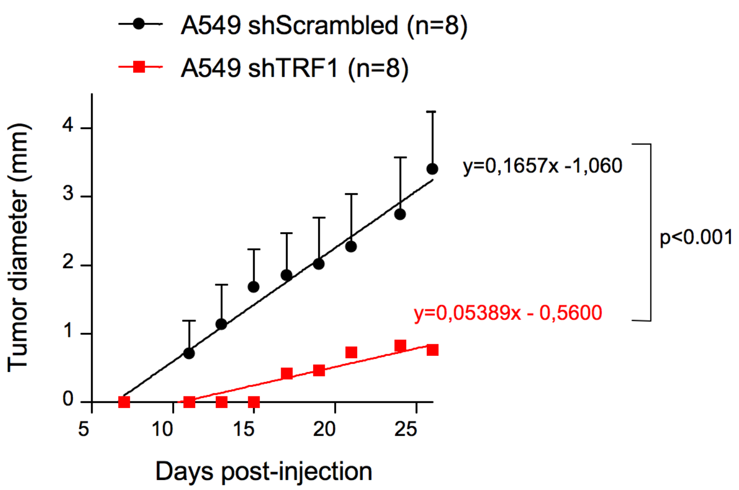

Supplementary Figure 4

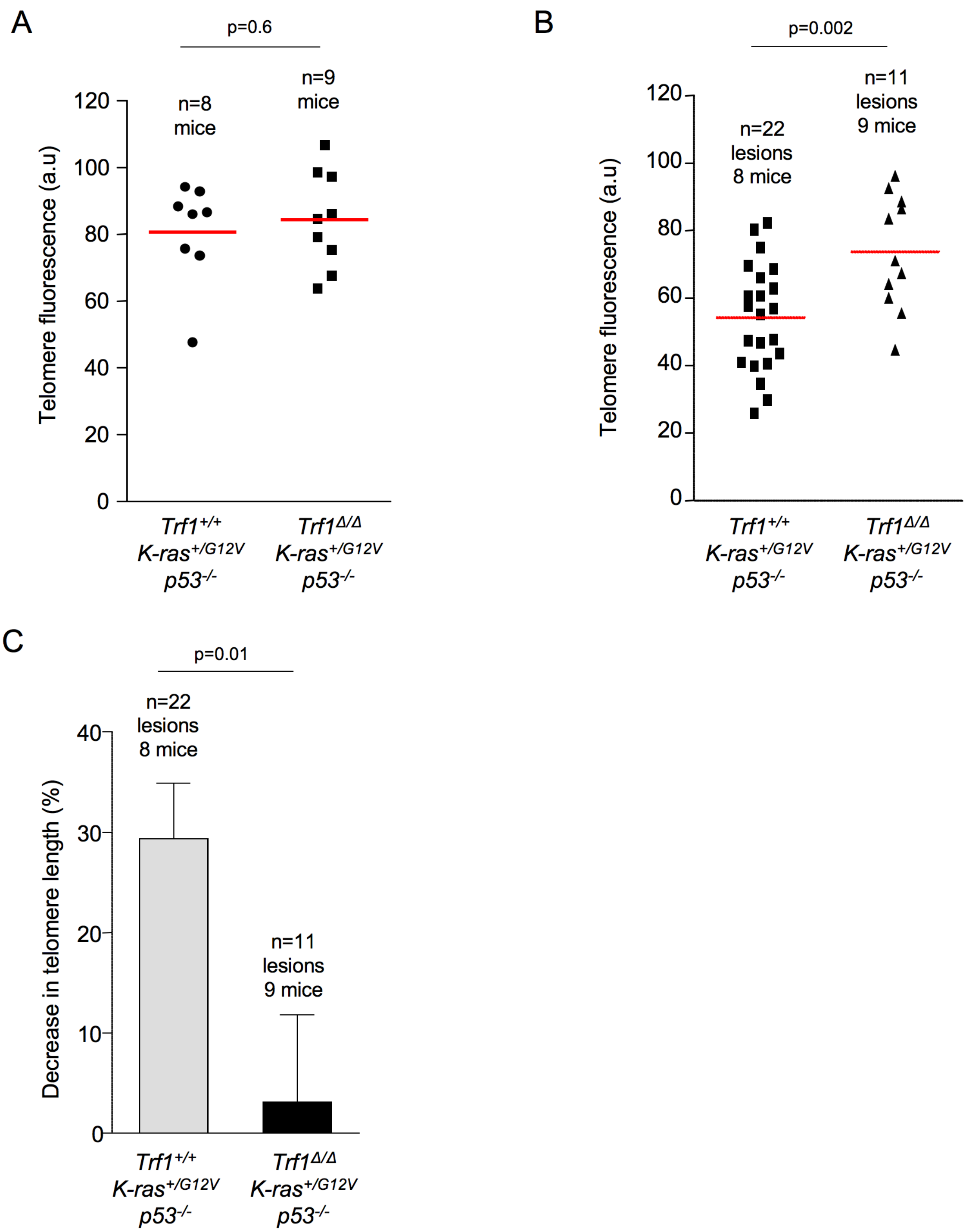

Supplementary Figure 5

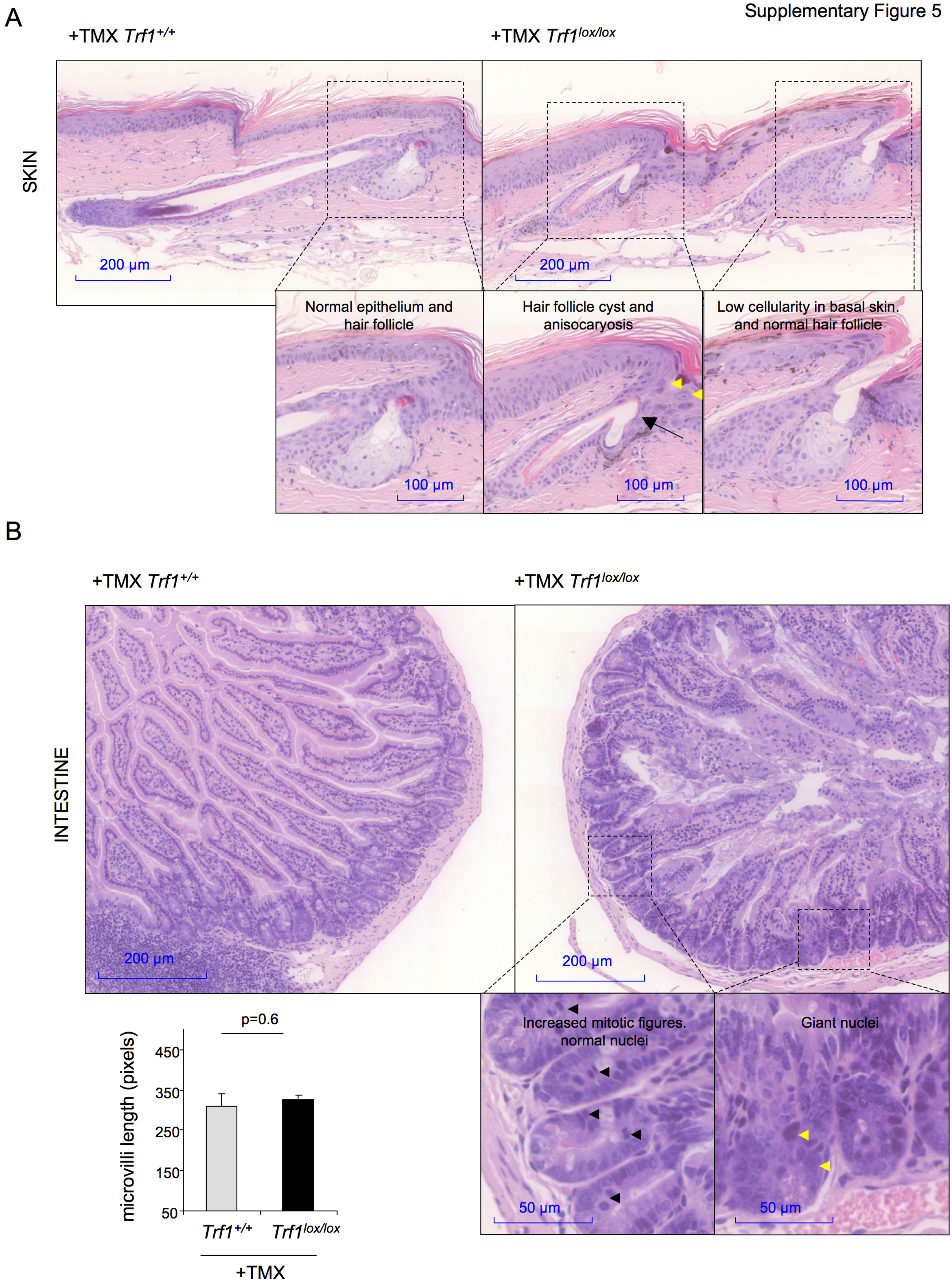

A

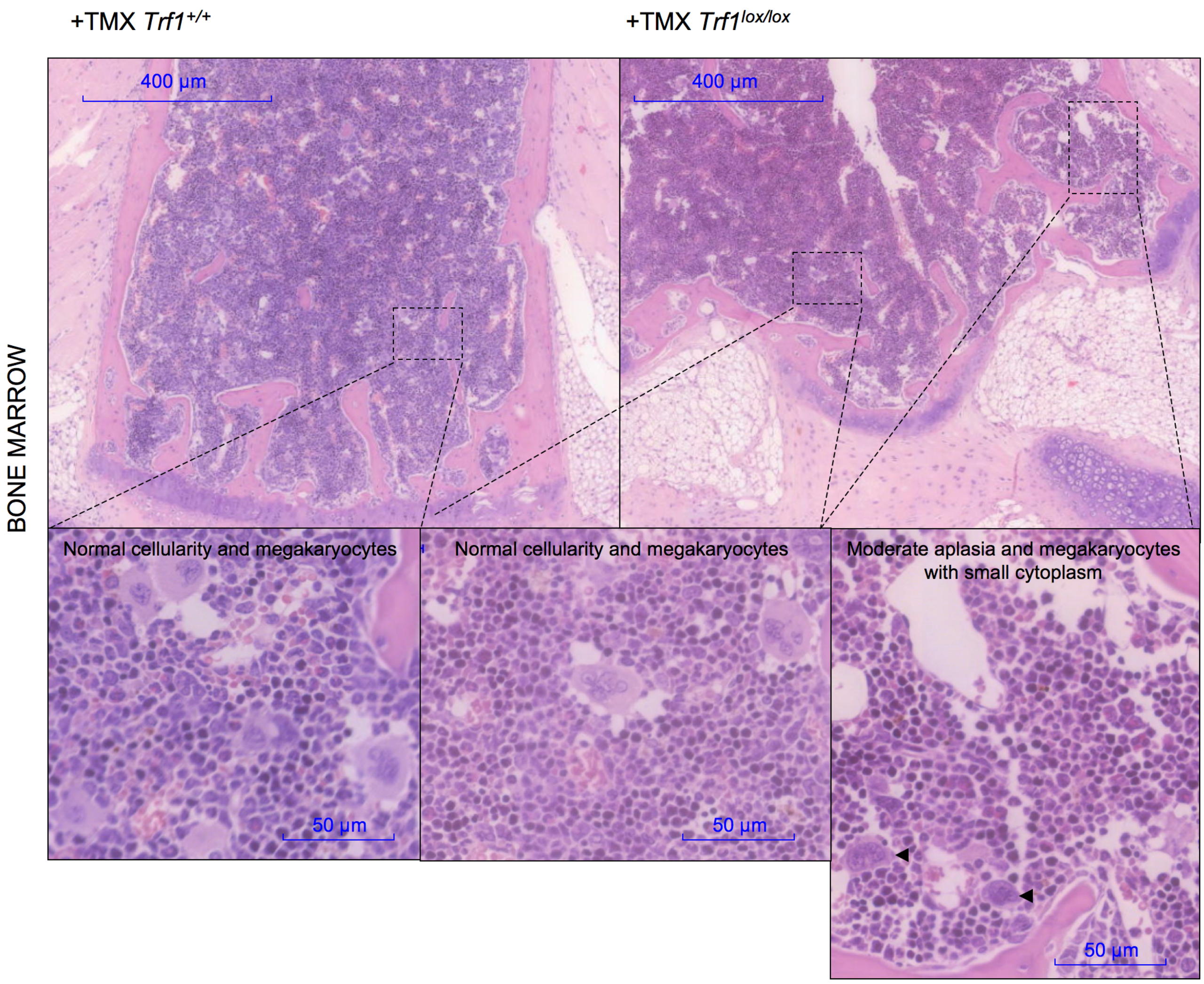

+TMX Trf1+/+
+TMX Trf1lox/lox
BONE MARROW
400 µm
400 µm
Normal cellularity and megakaryocytes
Normal cellularity and megakaryocytes
Moderate aplasia and megakaryocytes with small cytoplasm
50 µm
50 µm
50 µm

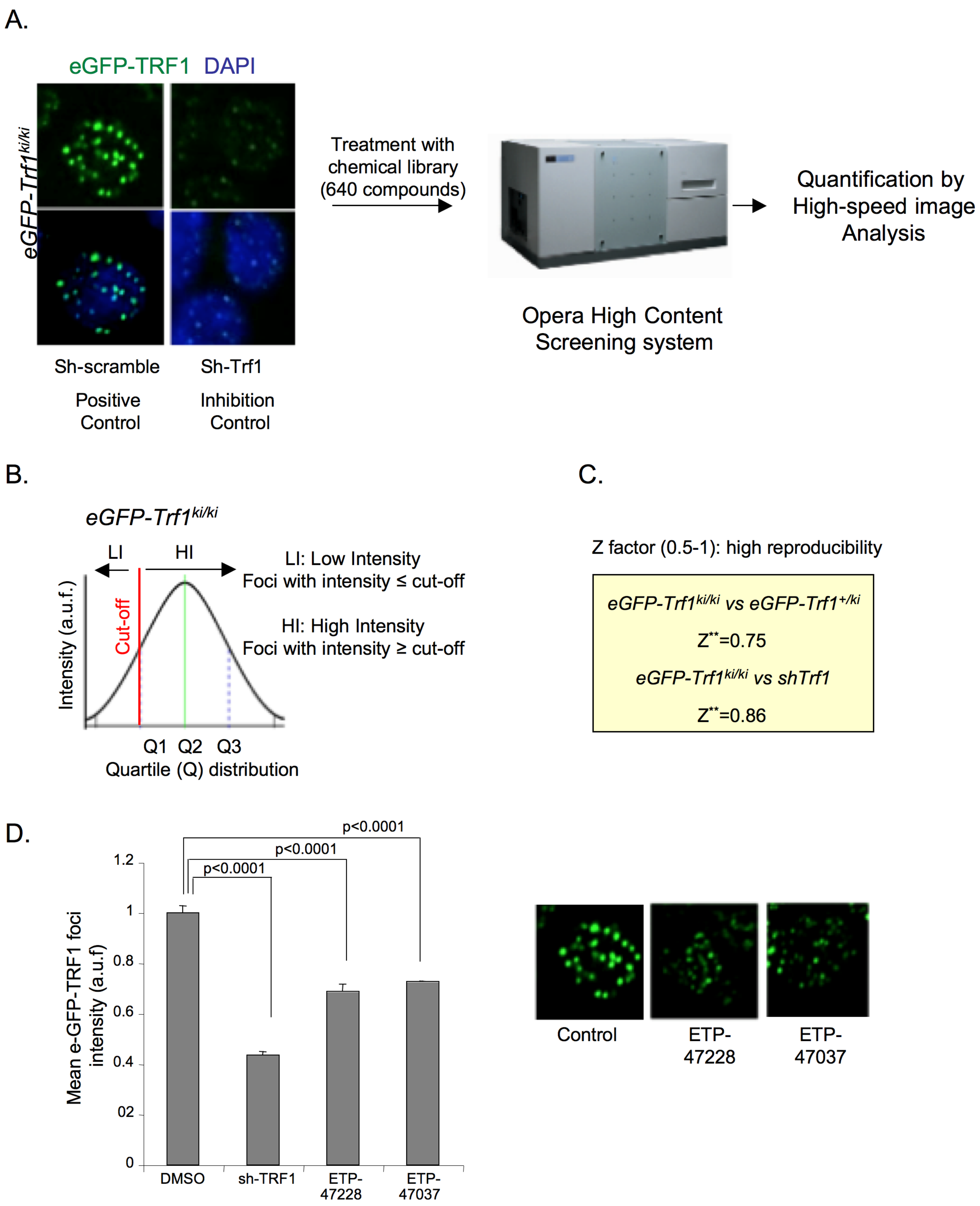
A.
eGFP-TRF1 DAPI
eGFP-Trf1ki/ki
Sh-scramble
Positive Control
Sh-Trf1
Inhibition Control
Treatment with chemical library (640 compounds)
Opera High Content Screening system
Quantification by High-speed image Analysis
B.
eGFP-Trf1ki/ki
LI
HI
Cut-off
Intensity (a.u.f.)
Q1 Q2 Q3
Quartile (Q) distribution
LI: Low Intensity
Foci with intensity ≤ cut-off
HI: High Intensity
Foci with intensity ≥ cut-off
C.
Z factor (0.5-1): high reproducibility
eGFP-Trf1ki/ki vs eGFP-Trf1+/ki
Z**=0.75
eGFP-Trf1ki/ki vs shTrf1
Z**=0.86
D.
Mean e-GFP-TRF1 foci intensity (a.u.f)
1.2
1
0.8
0.6
0.4
02
0
p<0.0001
p<0.0001
p<0.0001
DMSO
sh-TRF1
ETP-47228
ETP-47037
Control
ETP-47228
ETP-47037

Supplementary Figure 8

A

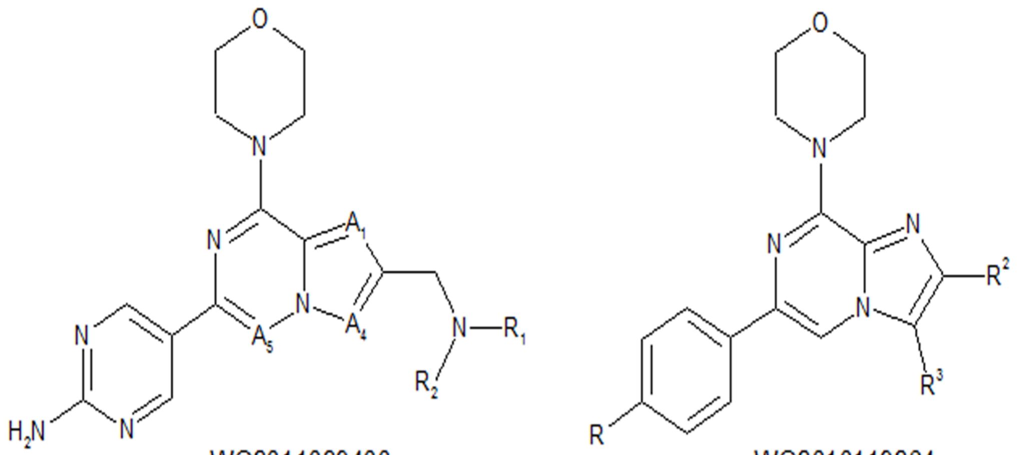

B

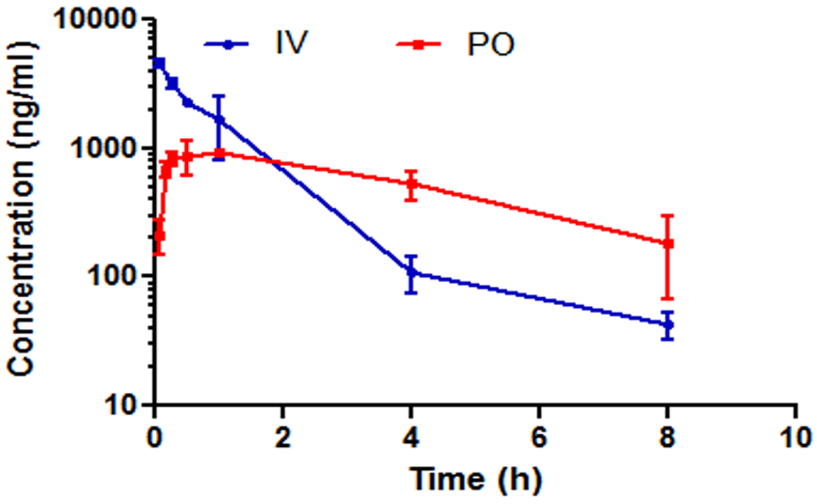

C

Time(hr)	I.V (ng/ml)	Oral (ng/ml)
0.083	4603.5	211.8
0.25	3196.8	823.6
0.5	2300.7	872.4
1	1667.2	938.4
4	109.1	527.4
8	42.7	182.1

D

Parameter	I.V.	PO
F%	-	29.5
Cmax (ng/ml)	-	938.45
Tmax (h)	-	1
AUC inf (h*ng/ml)	5820.64	5151.05
T½ (h)	0.51	5.2
CL(L/h/kg)	0.65	-
Vd (L)	0.015	-
MRT (h)	1.13	-

Supplementary Figure 9

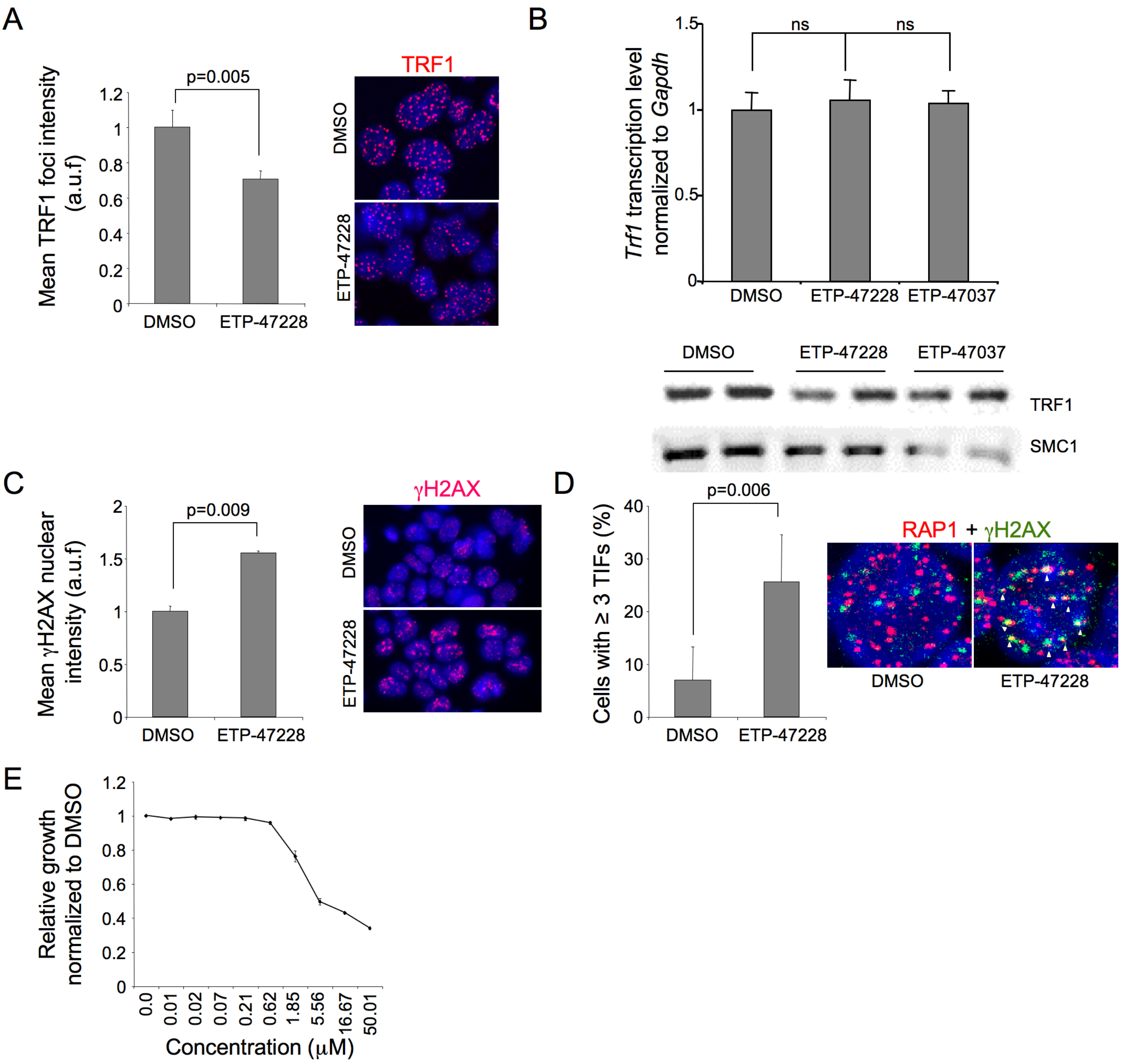

Supplement: Supplementary file 1 [file emmm0007-0930-sd1.pdf]
