# Supplementary material for: Therapeutic inhibition of TRF1 impairs the growth of p53-deficient K-RasG12V-induced lung cancer by induction of telomeric DNA damage
Source: EMBO Mol Med. 2015 May 13;7(7):930–49. doi: 10.15252/emmm.201404497 (PMC4520658; doi:10.15252/emmm.201404497)
Supplement: Supplementary file 2 [file emmm0007-0930-sd2.pdf]

EMBO Molecular Medicine

Therapeutic inhibition of TRF1 impairs the growth of p53-deficient K-Ras^{G12V}-induced lung cancer by induction of telomeric DNA damage

María García-Beccaria, Paula Martínez, Marinela Méndez-Pertuz, Sonia Martínez, Carmen Blanco-Aparicio, Marta Cañamero, Francisca Mulero, Chiara Ambrogio, Juana M. Flores, Diego Megias, Mariano Barbacid, Joaquín Pastor, and Maria A. Blasco

Corresponding author: María *A. Blasco, CNIO*

Review timeline:

Submission date:	25 April 2014
Editorial Decision:	23 May 2014
Appeal	28 May 2014
Editorial decision	30 May 2014
Additional author correspondence:	03 July 2014
Resubmission:	31 July 2014
Editorial Decision:	01 September 2014
Appeal:	05 September 2015
Editorial Decision:	11 September 2015
Additional author correspondence:	17 September 2015
Additional editorial correspondence :	24 September 2015
Appeal:	21 October 2015
Editorial Decision:	30 October 2015
Revision received:	14 January 2015
Editorial Decision:	13 March 2015
Revision received:	20 March 2015
Editorial Decision:	27 March 2015
Accepted:	02 April 2015

Transaction Report:

(Note: With the exception of the correction of typographical or spelling errors that could be a source of ambiguity, letters and reports are not edited. The original formatting of letters and referee reports may not be reflected in this compilation.)

Editor: Roberto Buccione

1st Editorial Decision: 23 May 2014

Thank you for the submission of your manuscript to EMBO Molecular Medicine.

We have now received feedback from the three Reviewers whom we asked to evaluate your manuscript.

As you will see, all Reviewers are globally unsupportive to various degrees and with serious, mostly overlapping concerns that I am afraid, preclude publication of the manuscript in EMBO Molecular Medicine. I will not discuss each point in detail as they are clearly stated and I will just go over the main points.

All three Reviewers, albeit with different takes, are not convinced that the manuscript provides clear translational potential, which of course is of relevance for our title. For instance, Reviewer 1 maintains that genetic ablation of a protein, especially if not enzymic in nature, does not per se imply its value as a target for therapy compared to others. Similarly, Reviewer 2, while providing a detailed list of important concerns that in principle are addressable, again appears doubtful of the therapeutic potential of the work. Reviewer 3, finally, is quite negative and mentions both the limited conceptual novelty of the findings and, essentially with the same argumentations of Reviewer 1, does not see the translational implications of the findings.

In addition to the above general concern, Reviewers 1 and 2 point to additional issues. The first notes that the results obtained in the p53-deficient setting are at odds with those obtained in the p53-proficient one. Reviewer 2 also raises several questions on the very same issue and also mentions other items of concern.

Given these fundamental issues and the overall lack of enthusiasm, and after discussion with my colleagues. I have no choice but to return the manuscript to you so that you may seek an alternative venue for your work.

I am sorry to have to disappoint you, and hope the Reviewer comments are useful for your continued work on the topic.

Appeal 28 May 2014

Many thanks. We have read in detail the criticisms and, we found the commentaries by the reviewers (particularly, reviewers #1 and #2) very detailed, insightful, and helpful. Indeed, we were very glad, that both reviewers #1 and #2 found the work well-performed, of interest, and of importance:

#reviewer1 states: "Experiments are well performed", "Targeting telomeres is a goal pursued for a long time", and "The authors might be right that targeting shelterin is an interesting approach to compromise telomere function".

reviewer 2 states: "This work is clearly a very interesting and important follow up, implicating now and important aspect for TRF1 in tumor growth as well".

However, we agree with you that, in addition to a series of minor comments, the reviewers have three major concerns:

1) We should demonstrate that it possible to target a shelterin protein in a drug screening program, thus the findings have a translational potential.

2) We should generate a new mouse model to show that TRF1 deletion in adult mice is not deleterious, thus supporting the potential use of TRF1 inhibitors in cancer treatment.

3) We should demonstrate that TRF1 deletion also inhibits human cancer growth.

We were glad to read these commentaries, as these

were also our initial concerns and, as a matter of fact, we have already answered many of them:

1) At the Experimental Therapeutics Program of the CNIO (a state-of-the-art drug development program at the center) we have already identified hit compounds which disrupt TRF1 binding to telomeres in cellular assays. These have been identified by screening compound libraries in a high throughput imaging platform by using cells derived from a eGFP-TRF1 reporter mouse, in which we can follow TRF1 intensity at telomeres (Schneider et al., Nature Communications, 2013).

I am attaching a pdf wurg the screening platform and the hits that we are developping now into lead compounds. We think that with this information we can make a strong case to the reviewers that it is indeed possible to target TRF1 and that we have the right tools for doing that.

2) As an important control to further engage in the development of TRF1 inhibitors, we have already generated a new mouse model in which we deleted TRF1 in the adult organism by tamoxifen addition to the drinking water. After 1,4 months of treatment, the mice show normal viability, suggesting that temporary TRF1 deletion in normal adult mice is not deleterious, in contrast to what has been observed when TRF1 deletion is performed since early embryo development. We can include these new important data in the manuscript.

3) We are inhibiting TRF1 in human cancer cell lines by shRNAs against human TRF1 and the initial results are that TRF1 inhibitions strongly blocks their growth. We can complete these initial results with mouse xenograph models and include this in the manuscript.

I will send a formal rebuttal including these new data as well as detailed answer to the reviewers in the following couple weeks. I hope that you consider that our arguments are strong for rebuttal,

2nd Editorial decision 30 MAY 2014

Thank you very much for your message and attachment.

Together with my colleagues, I look forward to receiving and considering in depth the detailed rebuttal when its ready. I'm sure you will understand that I cannot make any decision at this time on whether the rejection of the manuscript should be reversed and further review encouraged.

Additional author correspondence 03 July 2014

Sorry for the delay, we have finished already the full characterization of the whole-body conditional TRF1 deletion in adult mice, which is very important to asses toxicity of TRF1 inhibition. We are finishing the experiments with TRF1 downregulation in human cancer cell lines (xenographs etc), which was also requested by one of the reviewers. We can send the manuscript in two weeks time.

Resubmission 31 July 2014

[AUTHORS] We would like to start this point by point response letter to the reviewer's suggestions by thanking them for the careful examination of our manuscript and for their interest in our work. We strongly believe that all the raised points are useful and have helped us reinforce some notions and clarify some concepts that needed review in our original manuscript. Now our revised

© EMBO

manuscript has improved and we hope that you appreciate the improvements done in the underscoring of the results we present in this work.
We find particularly revealing the following statements made by the two reviewers:

[Reviewer #1] **"Experiments are well performed"**, **"Targeting telomeres is a goal pursued for a long time"**, and **"The authors might be right that targeting shelterin is an interesting approach to compromise telomere function"**.
[Reviewer #2] **"This work is clearly a very interesting and important follow up, implicating now and important aspect for TRF1 in tumor growth as well"**.

Referee #1 for EMM-2014-04198:

Comments on Novelty/Model System:
1. Experiments are well performed (more are needed).
2. Targeting telomeres is a goal pursued for a long time.
3. Unclear whether this ever will lead to applications. Many, many uncertainties.

Remarks:

The authors describe the consequences of inhibiting Trf1 in a conditional mutant Kras mouse model for lung cancer. Telomeres is widely viewed as a potential target for cancer therapy as its attrition results in mitotic catastrophe and cell death and therefore could contribute to cancer cell killing assuming that normal tissue stem cells do not suffer to the same extent. However, it has proven difficult to achieve this. Furthermore, mouse studies have shown that p53 proficiency is required to effectively kill tumor cells with short telomeres. In this manuscript the authors have used a conditional Trf1 mouse to show that depletion of Trf1 might be even more promising than telomere attrition as observed by loss of telomerase activity in Terc knockout mice (after 5 generations in view of the long telomeres of laboratory mice). These previous studies explored the concept that once telomeres are short in the whole organism additional shortening in fast dividing clonal expanding tumor cells results in a therapeutic benefit. In the current manuscript the authors tested whether acute telomere uncapping by Trf1 shelterin depletion would have a similar effect. It appeared that uncapping by depleting the Trf1 from the tumor cells resulted in a substantial inhibition of tumor growth, even when the tumors were p53 deficient. They argue that depleting shelterin might be a novel alternative approach for lung cancer treatment.

While this work is along the line of many other studies that attempt to exploit telomere attrition to inhibit tumor cell growth, the underlying question that the authors fail to address is not so much how to kill tumor cells but rather how to achieve an adequate therapeutic index leading to the preferential kill or slow down of tumor cell proliferation as compared to normal cells. The notion that adult stem cells have higher levels of Trf1 is therefore not an indication that Trf1 is an obvious target as the authors seem to imply.

[AUTHORS] We are very glad that the reviewer considers that "Experiments are well performed", "Targeting telomeres is a goal pursued for a long time", and "The authors might be right that targeting shelterin is an interesting approach to compromise telomere function". In addition, we appreciate the reviewer's thorough revision of our work and consider that his/her comments have been very useful for the improvement of our revised manuscript.

In particular, we fully agree with the reviewer suggestion that we had to address whether Trf1 inhibition in cancer offers an adequate therapeutic index preferentially killing cancer cells compared to normal healthy tissues. In order to address the impact of Trf1 deletion in the adult organism, we have generated a new mouse model, *Trf1$^{lox/lox}$ hUBC-CreERT2* mice, which allows whole-body Trf1 deletion in adult tissues upon administration of a tamoxifen diet. By using this model, we now demonstrate that mice with transient systemic deletion of *Trf1* in most adult tissues during more than 1.5 months show normal mouse viability without detectable signs of sickness or tissue dysfunction. Indeed, upon full histopathological analysis, we only find minor histological alterations, which are reverted upon removal of the tamoxifen diet and Trf1 re-expression (see **new Figure 8, new Supplementary Figs. 2-3**). We think that addition of these new data strongly improves the

manuscript and provides experimental demonstration *in vivo* that there is a therapeutic window for targeting Trf1 in cancer.

As the authors point out the effects of Trf1 depletion can have very different consequences dependent on the cell type and context. This emphasizes the importance of assessing the effects of temporary Trf1 depletion as one would envisage during cancer therapy (assuming that in the future there is a way to do this using small molecules).
Evidently, the earlier work by these authors has shown that Trf1 depletion in HSC is not tolerated and leads to severe depletion of stem cells. The urgent question then is whether temporary depletion in normal tissue (stem) cells will not permanently damage these cells.
Before claiming that this is an interesting approach for lung cancer treatment, one would like to see whether the temporary knockdown of Trf1 in all tissues will still substantially inhibit tumor growth without concomitant irreversible damage to normal tissues. Given their earlier work on Trf1 depletion, this information should be included in this MS to make it acceptable for publication in EMBO Molecular Medicine.

[AUTHORS] As suggested by the reviewer and discussed above, we now show that temporary downregulation of Trf1 in all adult tissues does not affect mouse viability or health. In particular, our *Trf1*$^{lox/lox}$ *hUBC-CreERT2* mouse model shows that a transient deletion of *Trf1* in the whole body is compatible normal survival and normal tissue function. Indeed, upon full histopathological analysis, we only find minor histological alterations, which are reverted upon removal of the tamoxifen diet and Trf1 re-expression (see **new Figure 8, new Supplementary Figs. 2-3**).

Regarding previous works from our group showing the consequences of Trf1 deletion in different tissue types, it is important to point out that in those previous works Trf1 deletion was induced at different developmental stages and conditions. In particular, we previously showed that Trf1 depletion in adult bone marrow used to reconstitute irradiated mice caused bone marrow failure after 2-3 months upon Trf1 depletion (Beier et al, 2012). It is important to point out that, in that study, *Trf1* was depleted from bone marrow used for bone marrow reconstitution of irradiated recipients. In this setting, the transplanted Trf1-deficient cells underwent a high proliferative stress in order to reconstitute bone marrow in the irradiated recipient, leading to stem cell exhaustion in the Trf1-deficient cohorts. In our current work, conditional deletion of *Trf1* in bone marrow during more than 1.5 months only causes mild alterations in blood cell counts and only in a few cases a moderate bone marrow aplasia (see **new Figure 8 and Supplementary Figure 3**). Furthermore, now we show that, removal of tamoxifen from the diet results in Trf1 re-expression and in regeneration of normal blood counts after 10 days (**new Figure 8f-g**).

In the experiment in which they deplete Trf1 in p53 proficient mice, the depletion of Trf1 leads only to tumors that have retained a wt Trf1 protein. They represent "escaper" clones. This indeed emphasizes the dependency on Trf1 in cells that are p53 proficient, although one rather would like to see tumor shrinkage of established tumors upon Trf1 depletion.
Given the strong effect of Tfr1 loss also in a p53-deficient setting it is puzzling why escapers are not found when they test loss of Trf1 in a p53-deficient setting. Also in this case tumor growth is vastly impaired and one therefore expects escapers also to be found in this condition. How do the authors explain this? Are all the tumor cells in the resulting lesions Trf1 deficient?

[AUTHORS] The reviewer has a good point and we have now included in the revised manuscript the analysis of the number of scapers in the p53-null setting. In particular, we also find escapers in the *Trf1*$^{lox/lox}$ tumors in the *p53*-deficient setting, but the percentage of escapers is much lower (only 5%) than the percentage of escapers found in the p53-proficient setting (100% of the tumors were scapers). We have included these new data in **new Figure 3b**. The most likely explanation for this difference is that absence of p53 allows for survival of genomically unstable *Trf1*-deleted tumour cells, a situation that recapitulates human cancer.

Regarding the effects of Trf1 deletion once the tumor is formed, in the revised manuscript, we now include extensive data showing that *Trf1* deletion in an already established mouse K-ras induced lung cancer cell line leads to decreased tumor growth and lower metastatic potential in mouse xenograph models (see **new Figure 7p-q**). Importantly, we have now extended these studies also to human cancer cell lines (see **new Fig.7p-q**). Future work also warrants generation of yet a third mouse model to allow Trf1 deletion in vivo once the tumors are formed. We now discuss these

issues in the revised manuscript (page 19, 3rd paragraph): *"Future works warrants generation of new mouse models to allow Trf1 deletion in vivo once the tumors are formed."*

The authors might be right that targeting shelterin is an interesting approach to compromise telomere function. But the same may hold for the other components of the shelterin complex that fulfill a role in protecting telomeres. Is Trf1 in this regard more promising than other components of the protective complex?

[AUTHORS] We agree with the reviewer in that targeting other shelterin components may constitute a likewise valid and potential strategy to kill cancer cells. We have chosen Trf1 for this proof of concept study because in our lab we have successfully developed a high throughput cellular assay to identify chemicals capable of inhibiting Trf1 binding to telomeres using cells derived from an eGFP-Trf1 knock-in mouse generated by us in which we can follow TRF1 intensity at telomeres (see **Figure 1 for the reviewers**) (see Schneider et al., *Nature Communications*, 2013). Indeed, by using this screening method, we have already indentified some chemicals able to decrease Trf1 binding to telomeres (see **Figure 1 for the reviewers**). These data constitute an independent project that is still ongoing in the laboratory in collaboration with the *Experimental Therapeutics Program* at CNIO (a state-of-the-art drug development program at the center).

Also depleting a protein by genetic means is very different from targeting a protein with small molecules. This is likely more so for proteins that have no enzymatic function. Given the scope of Molecular Medicine they should therefore discuss the practicalities of targeting this class of (more structural) proteins and indicate what additional validation experiments will be needed to justify a drug screening program or other strategy to deplete Trf1 from lung tumors.

[AUTHORS] As stated in the previous point, we have already identified hit compounds, which disrupt TRF1 binding to telomeres in cellular assays in collaboration with the *Experimental Therapeutics Program* at CNIO. These compounds have been identified by screening compound libraries in a high throughput imaging platform by using cells derived from a eGFP-TRF1 reporter mouse, in which we can follow TRF1 intensity at telomeres (Schneider et al., *Nature Communications*, 2013) (see **Figure 1 to reviewers**). We think that identification of these compounds is further proof of concept that it is possible to chemically target the binding of Trf1 to telomeres.

Referee #2 for EMM-2014-04198:

Remarks:

After having deleted TRF1 in a number of systems such as stem cell compartments, bone marrow, or stratified epithelia, the authors now concentrate on lung cancer and present "Effective impairment of K-RasG12V-induced lung carcinomas by abrogation of the TRF1 shelterin component". Garcia-Beccaria and coworkers present a mouse model for K-Ras driven lung tumorigenesis and report that ablation of the telomere repeat-binding factor TRF1 blocks tumorigenicity in a p53 wildtype background and reduces the grade of malignancy and tumor size in p53-deficient mice. The anti-tumorigenic effect was associated with dysfunctional telomeres and apoptosis, reduced proliferation, G2-arrest, and endoreduplication. Additionally, they used a lung carcinoma cell line established from K-Ras+/p53-/- mouse to demonstrate that partial down-regulation of TRF1 also impairs tumor growth and metastases in the allograft tumor model. From the outcome of their study they conclude that telomere uncapping, i.e. inducing dysfunctional telomeres emerges as a potential new therapeutic target for human lung cancer. This work is clearly a very interesting and important follow up, implicating now an important aspect for TRF1 in tumor growth as well.

However there are two conceptual facts that should be addressed:

First, the authors described before that MEFs abrogated for TRF1 showed senescence but not apoptosis. Deleting TRF1 in stratified epithelia of TRF1D/DK5-Cre mice, these mice died perinatally, showed skin hyperpigmentation (melanocyte upregulation) and epithelial dysplasia, associated with the induction of telomere-instigated DNA damage, activation of the p53/p21 and

p16 pathways, and cell cycle arrest in vivo. p53 deficiency rescued mouse survival but led to the development of squamous cell carcinomas (Martinez et al., GENES & DEVELOPMENT 23:2060-2075, 2009).

Conditional TRF1 knockout in the hematopoietic compartment caused bone marrow failure and recapitulates clinical features of dyskeratosis congenita. (Beier, et al., Blood. 120:2990-3000, 2012).

As being particularly enriched in stem cell compartments of highly regenerative tissues, they further showed that ablation of TRF1 caused severe GI atrophy (Schneider et al., Nature Communication, 4: 1946, 2013).

[AUTHORS] We thank the reviewer for considering that "This work is clearly a very interesting and important follow up, implicating now an important aspect for TRF1 in tumor growth as well". In addition, we thank the reviewer for his/her insightful suggestions and commentaries, which we have incorporated in the revised manuscript.

The reviewer is correct in pointing to various Trf1-deficient mouse models generated previously by us where we deleted Trf1 at different developmental stages and using different conditions. As in those settings *Trf1* deletion was deleterious, in the revised manuscript, we set to address here whether Trf1 inhibition in cancer offers an adequate therapeutic index preferentially killing cancer cells compared to normal healthy tissues. In order to address the impact of Trf1 deletion in the adult organism, we have generated a new conditional mouse model, *Trf1*$^{lox/lox}$ *hUBC-CreERT2* mice, which allows whole-body Trf1 deletion in adult tissues upon administration of a tamoxifen diet. This allows us to delete Trf1 from adult tissues in a controlled manner. By using this model, we now demonstrate that mice with transient systemic deletion of *Trf1* in most adult tissues during more than 1.5 months show normal mouse viability without detectable signs of sickness or tissue dysfunction. Indeed, upon full histopathological analysis, we only find minor histological alterations in a few mice, which are reverted upon removal of the tamoxifen diet and Trf1 re-expression (see **new Figure 8, new Supplementary Figs. 2-3**).

Regarding Trf1 deletion in the bone marrow, it is important to point out that in our previous work by Beier et al. (2012), we deleted Trf1 in adult bone marrow used to reconstitute irradiated mice. We observed that Trf1 deletion caused bone marrow failure 2-3 months after reconstitution of irradiated recipients (Beier et al, 2012). However, in that setting, the transplanted Trf1-deficient cells underwent a high proliferative stress in order to reconstitute bone marrow in the irradiated recipient, leading to stem cell exhaustion in the Trf1-deficient cohorts. In contrast, in the setting of the newly generated *Trf1*$^{lox/lox}$ *hUBC-CreERT2* mouse model in the revised manuscript, we now show that conditional deletion of *Trf1* in bone marrow during more than 1.5 months only causes mild alterations in blood cell counts and only in a few cases a moderate bone marrow aplasia (see **new Figure 8 and Supplementary Figure 3**). Furthermore, we also show that removal of tamoxifen from the diet results in Trf1 re-expression and in regeneration of normal blood count after 10 days (**new Figure 8f-g**).

Here they show an important role for escapers in tumor growth in p53-proficient mice and in p53-deficient mice the number of TRF1-deficient tumors was even twice the number of that of TGF1-wild-type in p53 proficient. Furthermore, TRF1-depletion induced a high frequency of genomic instability. These points may well be taken into consideration and should be discussed when proposing that "acute telomere uncapping by TRF1 emerges as a potential new therapeutic target for lung cancer".

[AUTHORS] The reviewer has a good point. Although in a p53-deficient background *Trf1* deletion results in higher number of very small and benign lesions, these lesions do not progress to malignancy or affect mouse viability (**Fig. 3 and 4**). From a therapeutic point of view these data suggest that Trf1 inhibition could be effective to impede tumor growth and progression. Indeed, we show that inhibition of Trf1 in cell lines derived from mouse K-ras induced lung carcinomas effectively blocks tumor growth and metastasis in allograft models (see **new Fig. 7**). In the revised manuscript, we have extended these observations also to human lung cancer xenograph models in which we have downregulated Trf1 expression (see **new Fig. 7**).

Second: Foregrounding human lung cancer, a second inapprehensible fact is that the authors validated their mouse data with a murine allograft model, i.e. by using a mouse lung cancer cell line from the same mouse model. As proof of principal and to address the relevance of their findings for human lung cancer one would have expected to see this work done with human lung cancer cells in a xenograft model instead.

[AUTHORS] We appreciate reviewer's comment and we have now included in the manuscript a xenograft model with human lung cancer derived cell lines in which Trf1 expression has been knocked down by shRNAs (see **new Fig.7p-q**). The results with human cell lines recapitulate the results obtained with mouse cancer cell lines, namely that depletion of Trf1 levels effectively impairs tumor growth.

Specific comments:

1. As extensively demonstrated recently, TRF1 depletion results in dysfunctional telomeres, failure of proliferation due to rapid induction of senescence, increased telomere damage and dysfunctional telomeres, sister chromatid fusions, chromosomal fragility (Martinez et al., 2009). The difference here is that the MEFs additionally express mutant K-Ras. It should be clarified whether and how additional K-Ras effects the MEFs or whetherTRF1 depletion is prominent and induces a pheno-/genotype irrespective of other genetic changes.

[AUTHORS] Trf1 plays an essential role in telomeric DNA replication (Martinez et al., 2009; Sfeir et al., 2009). K-ras oncogene induces replicative stress by forcing cell cycle progression and cell proliferation. Indeed, *Trf1*$^{\Delta/\Delta}$ *K-ras*$^{+/G12V}$ compound mutant cells show a significant increase in senescence as compared to single *K-ras*$^{+/G12V}$ mutants (**Fig. 1c**). We now discuss this in the revised manuscript text (page 9, 1st paragraph); "*This increase in senescence is probably due to the additive effect of K-ras oncogene-induced senescence and the Trf1 deficiency-induced senescence (Martinez et al, 2009; Serrano et al, 1997).*"

2. Fig 2B may be altered in is presentation as even in the normal tissue the long mouse telomeres (red staining for TRF1) are hardly detectable.

[AUTHORS] As suggested by the reviewer, the figure has been modified. Higher quality images have now been included (**new Figure 2b**).

3. Fig. 3 shows the strong selection pressure for tumor growth in vivo. In the p53-wildtype background TRF1-positive cells escape (likely causing the initial lag phase?) and form similar, though at a lower number, tumors as in the TRF1-wildtype mice. What is the histopathology of those tumors, are they identical?

[AUTHORS] Yes, they are histologically identical. This has been mentioned in the text (page 10, 2nd paragraph); "Post-mortem lung analysis revealed that *Trf1* wild-type and *Trf1*-deleted tumors were histologically identical."

In the p53-/- background, the author find smaller, slowly growing tumors. However, absolutely it is already double the number than in p53-wildtype mice. Furthermore, as there is a clear increase in tumor number starting with week 16 it remains important whether the authors considered to extend the observation period in order to exclude that this growth pattern and tumor phenotype is not only attributed to an extended lag-/ selection phase? The authors make a strong statement that all TRF1-deficient p53-deficient tumors are benign adenoma lesions rather than full-blown carcinomas and discuss this as a dramatic impairment of progression of these benign lesions. This would implicate that NSCLC carcinomas generally develop in a step-wise fashion instead of de novo development. Could this statement be substantiated further by long-term observation (see above), genetic analyses, or rescue of TRF1 in the tumors? This is of crucial importance in the light of the author's proposal that "telomere uncapping emerges as a potential new therapeutic target for lung cancer".

[AUTHORS] We agree that it would be interesting to prolong the study to further follow the progression of Trf1-deleted lesions in the p53-deficient background. But unfortunately, given that p53-deficient mice have a maximum life-span of 6 months due to lymphomas/sarcomas development, it is impossible to prolong the experimental time of analysis.

We want to point to this reviewer that in the current manuscript we have extensively studied the effects of Trf1 deletion once the tumor is formed. In particular, we include extensive data showing that *Trf1* deletion in an already established mouse K-ras induced lung cancer cell line leads to decreased tumor growth and lower metastatic potential in mouse xenograph models (see **new Figure 7p-q**). Importantly, in the revised manuscript, we have now extended these studies also to human cancer cell lines (see **new Fig.7p-q**). Future work also warrants generation of yet a third mouse model to allow Trf1 deletion in vivo once the tumors are formed. We now discuss these issues in the revised manuscript (page 20, 2nd paragraph): *"In line with these observations, generation of a new mouse model to allow Trf1 deletion in vivo once the tumors are formed should be of great interest."*

4. Except for figure 4 and 7L bars are missing.

[AUTHORS] We are afraid there must have been a PDF editing error as the original figures have the bars.

5. Fig. 5: Is the distribution of É¡H2AX -positive cells in the TRF1-definent tumor homogeneous throughout the tumor or as it may appear from figure 5A more focal? Again this is asked in the light of potential selection of certain tumor areas? Fig.5B is confusing. Is RAP1 similarly affected upon TRF1-depletion? Telomere-dependent colocalisation should thus be shown by telomere FISH. It also remains unclear from the specific picture whether this is one potential apoptotic nucleus or whether this shows several nuclei that somehow fuse? Please clarify.

[AUTHORS] Rap1 localization at the telomere is well known not to be affected by *Trf1* deletion (Martinez et al., 2009; and Sfeir et al., 2009). We agree with the reviewer in that image shown the nucleus seems to be fragmented resembling an apoptotic nucleus. However, this is not the case and to avoid confusion we now show a higher quality picture (**new Figure 5b**).

6. Fig 5C: Apoptosis appears to be generally very low and this is well in line with what the authors showed before that senescence is more relevant. Even if statistically significant one may be careful to over-interpret the importance of rise from 0.4% to 0.6% of apoptotic cells.

[AUTHORS] We agree with the reviewer that the observed increase in apoptosis cannot be the only underlying cause for the observed inhibition of tumor growth. Senescence and mitotic catastrophe are also likely to contribute to the observed impairment of tumor growth. We have now tuned down this statement in the revised manuscript text (page 20, 1st paragraph); *"we show here that Trf1 deficiency results in a high burden of telomeric DNA damage, genetic instability, proliferation defects, apoptosis and mitotic catastrophe"*

7. Fig. 6B may have been a mistake by pdf conversion as the upper picture was black except for the red arrowheads.pH3 signals also appear to be present in some stromal cells. Is the stroma also affected by TRF1-depletion in the tumor cells - altered crosstalk?

[AUTHORS] We apologize for this editing error. However, we do not observe differences in stromal cells when comparing $Trf1^{+/+}$ and $Trf1^{\Delta/\Delta}$ tumors. We have included new representative images to illustrate this in **new Figure 6b**.

Figure 6D and E: the authors find 80% to 90% abnormal mitoses in TRF1-deficient tumors. Doesn't this explain the low growth rate as only few of those cells may be able to continue?

[AUTHORS] We fully agree with the reviewer and failed mitosis would eventually lead to cell death by mitotic catastrophe. This has been discussed in the text (page 13, 2nd paragraph); "*$Trf1^{\Delta/\Delta}$ K-$Ras^{+/G12V}$ $p53^{-/-}$ carcinomas presented cells showing bizarre multilobulated nuclei and multipolar mitosis, which were not present in $Trf1^{+/+}$ K-$Ras^{+/G12V}$ $p53^{-/-}$ carcinomas (**Fig. 6e**). This type of aberrant nuclei have been previously related to mitotic catastrophes (Vakifahmetoglu et al, 2008). Together, these observations indicate that lung carcinoma cells require Trf1 for proper completion of mitosis, and that Trf1 deletion in these cells leads to severe mitotic defects, similar to those produced by known mitotic poisons (Dumontet & Jordan, 2010; Jordan & Wilson, 2004; Manchado et al, 2012)"*

Second, this strongly argues for an extensive and obviously ongoing genomic instability. Does this fact not even support the requirement for a long-term follow up in the mice?

[AUTHORS] Unfortunately, it is impossible to prolong the experimental time of analysis, since the p53-deficient mice start to develop lymphomas and sarcomas resulting in early death at 4-6 months of age.

8. Finally, and as stated above, validation of the mouse findings should be performed with human cells rather than a lung cancer cell line from the same genetic mouse strain. Concerning the study with the mouse cell line, the control remains unclear. Does it contain control shRNA and thereby was subjected to the same selection process?

[AUTHORS] As suggested by the reviewer, in the revised version we have included xenograft experiment with a human lung cancer cell line in which *Trf1* expression has been knocked down by shRNAs (see **new Fig. 7p-q**).

The control corresponding to mouse cancer cell lines are the same mouse cell lines infected with sh-scrambled vectors and thereby subjected to similar selection process. We indicate this now in the revised manuscript text (page 15, 3rd paragraph); "*We then injected subcutaneously in immunodeficient mice 150,000 cells either infected sh-scrambled or Trf1-shRNA and followed tumor development*".

Referee #3 for EMM-2014-04198:

Comments on Novelty/Model System:
The findings presented herein are incremental and have been shown by others in many experimental models. The deletion of an essential gene leading to cell death in a tumor is simply not an advance nor is it the basis for nominating an approach for therapy.

Remarks:

In this manuscript, Blasco and colleagues describe the consequences of deleting the telomere binding protein TRF1 in a KRAS driven lung cancer model. Using both animals in which p53 is intact or deleted in concert with KRAS mutation, they show that co-deletion of TRF1 reduces (p53 null) or blocks (p53 intact) lung tumors. Tumors from animals in which TRF1 was deleted showed increased telomere localized DNA damage and decreased proliferation. At the same time, no differences in telomere length were found.

Although the authors argue that these findings provide a rationale for inducing telomere uncapping as an anti-neoplastic approach, the findings are not surprising, and it remains unclear whether this truly supports telomere uncapping as a therapeutic approach. Specifically, germline deletion of TRF1 was described in 2003 to lead to embryonic lethality (Karlseder et al, MCB, 2003, PMCID: PMC193696), and many subsequent reports have confirmed that TRF1 expression is necessary for cell autonomous cell survival. Thus, the finding that deletion of TRF1 in a specific tissue or in this case tumor leads to decreased survival or tumorigenicity is not surprising.

Moreover, because cell death is the dominant phenotype induced by deletion of TRF1, the authors cannot conclude that this effect is specific for the tumor type or genetic context. The deletion of any cell essential gene in a tumor is predicted to induce cell death. Since uncapping of telomeres will also affect normal tissues, it remains unclear how one would build on these observations as a therapeutic approach as argued by the authors.

[AUTHORS] The reviewer is correct in pointing to various Trf1-deficient mouse models previously generated by various groups including our group where Trf1 was deleted at different developmental stages and using different conditions. As in those settings *Trf1* deletion was deleterious, we agree with the reviewer that it is very important to address here whether Trf1 inhibition in cancer offers an adequate therapeutic index preferentially killing cancer cells compared to normal healthy tissues. In order to address the impact of Trf1 deletion in the adult organism, we have generated a new

conditional mouse model, *Trf1*$^{lox/lox}$ *hUBC-CreERT2* mice, which allows conditional whole-body Trf1 deletion in adult tissues upon administration of a tamoxifen diet. This allows us to delete Trf1 from adult tissues in a controlled manner. By using this model, we now demonstrate that mice with transient systemic deletion of *Trf1* in most adult tissues during more than 1.5 months show normal mouse viability without detectable signs of sickness or tissue dysfunction. Indeed, upon full histopathological analysis, we only find minor histological alterations in a few mice, which are reverted upon removal of the tamoxifen diet and Trf1 re-expression (see **new Figure 8, new Supplementary Figs. 2-3**). We think that addition of this new data strongly improves the manuscript and supports that there is a therapeutic window for targeting Trf1 in cancer.

In addition to the two mouse models generated de novo for this study (the specific deletion in the lung carcinogenesis model and the conditional deletion in the whole organism), we also show that inhibition of Trf1 in cell lines derived from mouse K-ras induced lung carcinomas effectively blocks tumor growth and metastasis in allograft models (see **new Fig. 7**). Importantly, in the revised manuscript, we have extended these observations also to human lung cancer xenograph models in which we have downregulated Trf1 expression by using shRNAs against Trf1 (see **new Fig. 7**).

Finally, although we used the *K-ras* lung cancer model to test our hypothesis of targeting a shelterin component as a cancer-treatment strategy, we fully agree with the reviewer that our conclusions might be extended for other types of tumors. This fact only highlights the importance of our findings. Indeed we have now included a sentence in "Discussion" mentioning the potential of this strategy in the treatment of several types of tumors (Page 21, 1st paragraph); *"Based on that this strategy relies on a universal mechanism, namely telomere dysfunction, we speculate that it could be applied in many other cancer types.*

As a final note, we would like to mention to this reviewer that we have already identified hit compounds, which disrupt TRF1 binding to telomeres in cellular assays in collaboration with the *Experimental Therapeutics Program* at CNIO. These compounds have been identified by screening compound libraries in a high throughput imaging platform by using cells derived from a eGFP-TRF1 reporter mouse previously generated by us, in which we can follow TRF1 intensity at telomeres (Schneider et al., *Nature Communications*, 2013) (see **Figure 1 to reviewers**). We think that identification of these compounds is further proof of concept that it is possible to chemically target the binding of Trf1 to telomeres.

Although the experiments described are reasonably clear, most of the work confirms what others have described in other cell lines or experimental models. Thus, this work is incremental over prior work in the field.

[AUTHORS] We disagree with this comment since to our knowledge this piece of work constitutes the first demonstration "in vivo" that targeting a shelterin component impedes tumor growth and development to malignancy in a well established K-ras induced lung carcinogenesis model, which is considered to faithfully recapitulate human lung cancer development.

3rd Editorial Decision 01 September 2014

Thank you for the re-submission of your manuscript to EMBO Molecular Medicine. We have now heard back from the three Reviewers whom we asked to evaluate your manuscript.

We apologise that it has taken more time than we would have liked to return a decision, but unfortunately we received the evaluations with some delay due to the vacation period. Furthermore, I wished to discuss your manuscript with my colleagues and our Head of Publications, Bernd Pulverer. You will see that specific concerns prevent us from considering publication.

Reviewer 1, while acknowledging the significant improvement of the current version, finds that the limitations of your original manuscript have not been fully addressed so that the main concerns stand. Specifically, s/he notes that the issue of the reversibility of Trf1 depletion remains unresolved based on the current data and also points to a serious caveat in the transient depletion approach employed. Reviewer 1 notes that a Dox-inducible or a double-switch system (as used previously by

others) would have been required to show that transient Trf1 depletion does not compromise the ability of tissue stem cells to maintain tissue homeostasis. A case in point is Reviewer 1's comment that the data on the effects of systemic Trf1 depletion on healthy tissues (Fig. 8) would have been of great value, including supporting the reversibility claim, if experiments had been performed with a small molecule treatment or proven reversible genetic inhibition (as mentioned above). Reviewer 1's initial criticisms were thoughtful, well-taken and constructive; we share his/her opinion that the main issue was simply not resolved thus significantly detracting from the potential novelty and translational value of your work.

Reviewer 3 (formerly Reviewer 2) is not satisfied that the human data provided are adequate to sustain a proof of principle in human (necessary given the very different telomere dynamics vs. mouse). Although this Reviewer suggests that the human data should be simply taken out at this point, we do agree however that some degree of validation in human cells is necessary to establish the translational potential of your findings (in addition to the issues mentioned above by Reviewer 1).

Reviewer 2 appears rather dismissive and does not fully explain his/her stance, whether shareable or not. Therefore, we have not taken into consideration his/her comments for our final decision.

I hope you will understand that considering the remaining fundamental concerns and also the continued lack of enthusiasm by Reviewers 1 and 3, we have no choice but to return the manuscript to you so that you may submit it elsewhere.

We are sorry to have to disappoint you at this stage of analysis and I hope that the Reviewer comments will be helpful in your continued work in this area.

***** Reviewer's comments *****

Referee #1 (Comments on Novelty/Model System):

The claim of reversibility cannot be answered with the model as now employed (with the experiments added in the revision). It would require an Dox inducible (sh RNA) or-even better- a double switch system (Cre/Lox + Flp/frt) as has been used by others in a different system to show that temporal depletion of Trf1 does not compromise the capacity of (tissue stem) cells to fulfill their replenishing task in maintaining tissue homeostasis.

Referee #1 (Remarks):

Overall the manuscript has been much improved. Unfortunately, there is still a major issue with the MS, that arises as a consequence of the additional experiments performed. The question whether temporal Trf1 depletion will be detrimental for normal tissue homeostasis remains unanswered even though the authors claim the opposite.

Indeed the short-term effects of tamoxifen-induced genetic deletion of Trf1 in all tissues are limited as one might expect for cells that do not divide. However, in the longer-term analysis of normal tissues the authors show that Trf1 levels - after initial depletion upon tamoxifen treatment- quickly recover (some additional information would be useful here: how the levels over time after cessation of the tamoxifen treatment compare with levels of animals not carrying a Trf1 floxed alleles). This implies that the cells that have actually retained full Trf1 function during tamoxifen treatment (failed to switch) are the ones that start to reconstitute the normal tissues. Therefore, it remains unclear whether tissue stem cells would be capable to re-gain their normal function after temporal Trf1 depletion since in the experiment shown the ones that actually are reconstituting the tissue never have suffered from Trf1 depletion. The authors seem not to have realized that there is an important conceptional difference between pharmacologic inhibition and incomplete genetic ablation in that the cells that repopulate the normal tissues never have suffered Trf1 depletion since the gene was not deleted in those cells (This is also indicated by the higher percentage of Trf1 positive cells in the crypts of the intestine (fig 8C)). In that regard these cells are reminiscent of the tumor escapers seen in the p53 proficient tumors.
Therefore, the question of the effect of temporal Trf1 inhibition has simply not been answered in

this in vivo setting. The notion that in p53-/- tumors only 5% of the escapers have retained a functional Trf1 allele suggests that the therapeutic index of this strategy could be actually quite low since the tumors apparently have ways to tolerate Trf1 depletion that are not accessible to normal p53-proficient cells.

The results the authors show in fig 8G would be great -and would support their conclusion of reversibility- if they were achieved upon small molecule treatment or proven reversible genetic inhibition (e.g. using lox/frt pairs). However, as presented the data simply do not support the rather strong claim of the authors that "All together, these data underline the reversibility of Trf1 depletion and support that a transient ubiquitous Trf1 downregulation is compatible with mouse viability".

Referee #2 (Comments on Novelty/Model System):

The deletion of TRF1 in this model is not informative for therapeutic considerations.

Referee #2 (Remarks):

In this lightly revised manuscript, Blasco and colleagues describe the consequences of deleting the telomere binding protein TRF1 in a widely studied KRAS-driven lung cancer models that do and do not harbor deletion of p53. Consistent with prior work they show that deletion of TRF1 induces cell death (in this case tumor death) in a manner that does not require overt telomere shortening. Based on these observations, the authors argue that this represents preclinical data to nominate TRF1 as therapeutic target in cancer.

The major concern from the first round of review is whether this represents a significant advance over what is known about deletion of TRF1 and whether this truly provides support for targeting TRF1 in cancer. The consequences of deleting TRF1 have been published in several settings, and it is known that this induces cell death. While it is true that specific deletion of TRF1 in KRAS-driven cancers has not been specifically shown, this observation is both incremental and expected.

The larger issue is whether these findings support the notion that targeting TRF1 in cancer is a reasonable approach. Deletion of ANY essential gene in a tumor will induce cell death and therefore this finding alone does not provide sufficient support for the conclusion that TRF1 is a good target. The authors have added data from a different mouse model in which deletion of TRF1 is accomplished by the inducible expression of the Cre recombinase. The experimental details of how TRF1 is deleted differ in important ways from the original experiments, and therefore it is difficult to see how this provides evidence that TRF1 deletion is a good target. Since the extent to which TRF1 is deleted nor the effect of such transient deletion on lung tumors are shown, it is not apparent how these new findings provide evidence that the observed results are merely the effect of deleting an essential gene in a tumor.

Thus although it is clear that substantial work went into creating and characterizing these models, the work presented in this manuscript fails to go beyond what is already known and does not provide sufficient evidence to support the notion that TRF1 is a therapeutic target in cancer.

Referee #3 (Comments on Novelty/Model System):

The authors have well responded to the questions addressed by the reviewers. Howver, my point on providing information on the human system is poorly addressed, though from my point of view overexagerated by the authors in their statements (see comment to the authors). As this is an important piece of work and the mouse studies very well perfomed, one may consider to publish this manuscript, however, by deleting the human part (see below). As done now, this is clearly not sufficient to support their statement.

Referee #3 (Remarks):

While the mouse work was excellently complemented and extended by the conditional whole-body TRF1 deletion in adult mice and was obviousl already prepared for resubmission, the work on the human cells appears to be initiated de novo and unfortunately remains spartan. The need for a proof of principle to test the hypothesis also for human cells is now prominently expressed in the Abstract and Statement of Significance. Unfortunately, the data behind this statement are very preliminary thereby missing the purpose of this question - namely the difference in regulation of telomerase and telomere length in human versus mouse.
- Not cell lines but a single cell line was used, the A549 cell line, without referring to the relevance of these particular cells.
- A549 cells infected with shTRF1 showed significant but not total reduction of TRF1 expression. How did this interfere with growth in culture? The cells were injected s.c. and a a graph is presented monitoring tumor growth. The only information provided is that after a lag phase of 17 days 2 tumors developed within the 8 injection sites. Unfortunately, no histology, no molecular characterization and mechanistic reason for tumor suppression are provided, nor is a description for the late escape of the tumor cells. As this is meant to prove the comparability of the human system and with that its potential for application in humans, these data are insufficient and do not legitimate the statement "Downregulation of TRF1 in cell lines derived from already established p53-deicient K-RAS lung carcinomas also impaired tumor growth and metastasis in allograft models, as well as in xenograph models of human lung cancer cell lines".
As the mouse work is well justified and very well done the authors may reconsider to include the at present unsatisfactory work on human cells and instead point to the fact that future studies with human cells have to show the relevance of these finding also for the human system - as this is crucial for proposing TRF1 as a therapeutic target for human lung cancer.

2nd Appeal 05 September 2015

Please, find attached our point-by-point rebuttal to the specific commentaries of reviewer #1.

Regarding the suggestion of reviewer #3 to remove the human cell line data from the final manuscript, we fully agree with the reviewer. As the reviewer #3 states, the new mouse data is much more extensive and solid.

As I mentioned before, I would like to have the chance to discuss the reviewer¥s commentaries with you.

I think that general perception of the reviewers is that this is a very important and novel manuscript (even reviewer #1 seems to agree with that), which will generate great interest in the translational community as it identifies shelterins as novel anti-cancer targets.

REBUTTAL TO REVIEWER #1

Referee #1 (Remarks):

Overall the manuscript has been much improved. Unfortunately, there is still a major issue with the MS, that arises as a consequence of the additional experiments performed. The question whether temporal Trf1 depletion will be detrimental for normal tissue homeostasis remains unanswered even though the authors claim the opposite.

We appreciate that reviewer 1 clearly states that **"the manuscript has been much improved"**. We do not agree however with reviewer's conclusion that the study of the impact on tissue homeostasis of temporal TRF1 depletion remains unaddressed. In fact, former reviewer #3, specifically praises

the new mouse work included in the revised manuscript.

In the revised manuscript we clearly show that:

1.- TRF1 expression levels have been successfully downregulated in a wide panel of adult mouse tissues by the generation of a new TRF1-conditional mouse model in which we induce TRF1 deletion by tamoxifen administration in the diet. IMPORTANTLY: we demonstrate TRF1 downregulation both by RTPCR and by immunofluorescence 7 weeks after TRF1 deletion.

2.- We clearly demonstrate no detrimental effects in tissue integrity and organismal viability seven weeks after TRF1 deletion. This was specifically requested by the reviewer.

Indeed the short-term effects of tamoxifen-induced genetic deletion of Trf1 in all tissues are limited as one might expect for cells that do not divide.

We do not agree with the reviewer's commentary since we clearly show TRF1 deletion upon tamoxifen treatment (seven weeks) not only in non-proliferating tissues (heart, lung, liver, kidney, brain) but also in highly proliferative ones such as intestine, skin, blood and bone marrow (Figure 8B).

However, in the longer-term analysis of normal tissues the authors show that Trf1 levels - after initial depletion upon tamoxifen treatment- quickly recover (some additional information would be useful here: how the levels over time after cessation of the tamoxifen treatment compare with levels of animals not carrying a Trf1 floxed alleles).

We could include the data regarding TRF1 expression levels in wild-type animals. We could certainly measure how TRF1 levels increases over time after cessation of the treatment. However, we think that the mayor point with this experiment is the lack of detrimental effects by TRF1 depletion in mouse viability and survival.

This implies that the cells that have actually retained full Trf1 function during tamoxifen treatment (failed to switch) are the ones that start to reconstitute the normal tissues. Therefore, it remains unclear whether tissue stem cells would be capable to re-gain their normal function after temporal Trf1 depletion since in the experiment shown the ones that actually are reconstituting the tissue never have suffered from Trf1 depletion.

We are fully aware that those cells that have excised TRF1 alleles by genetic means are not able to reconstitute the tissues. As the reviewer points and as we state in the manuscript, those stem cells that escaped TRF1 deletion are the ones regenerating tissues, especially those with high turn-over such blood, intestine, skin and bone marrow (Figure 8G).

The authors seem not to have realized that there is an important conceptional difference between pharmacologic inhibition and incomplete genetic ablation in that the cells that repopulate the normal tissues never have suffered Trf1 depletion since the gene was not deleted in those cells (This is also indicated by the higher percentage of Trf1 positive cells in the crypts of the intestine (fig 8C)). In that regard these cells are reminiscent of the tumor escapers seen in the p53 proficient tumors.

We obviously realize the differences between genetic ablation and pharmacological inhibition. As we reported to the reviewers, we are currently assaying some lead compounds against TRF1 identified by us in a chemical screen. However, this is a separated piece of work that is beyond the scope of this paper. In this work, we demonstrate for the first time that shelterin inhibition impairs cancer by deleting TRF1 in tumors using a well stablished K-RAS lung cancer model. Thus, targeting a shelterin component constitutes an interesting and novel strategy to impede tumor development. As a proof of concept, we show that transient depletion of TRF1 in the whole organism does not cause loss of mouse viability supporting that transient pharmacological TRF1 inhibition would not either lead to detrimental effects. Further studies are needed to identify and develop a drug capable to inhibit TRF1 function. Completion of such work requires long time and certainly will delay publication of our interesting findings.

Therefore, the question of the effect of temporal Trf1 inhibition has simply not been answered in this in vivo setting. The notion that in p53-/- tumors only 5% of the escapers have retained a functional Trf1 allele suggests that the therapeutic index of this strategy could be actually quite low since the tumors apparently have ways to tolerate Trf1 depletion that are not accessible to normal p53-proficient cells.

The reviewer has fully misunderstood this result. It is clear from our data that p53-negative cells are more tolerable to TRF1-loss than p53-proficient ones. However, we clearly show that tumor development, growth and progression to malignity are strongly compromised in p53-deficient cells lacking TRF1 as compared to TRF1-proficient cells.

The results the authors show in fig 8G would be great -and would support their conclusion of reversibility- if they were achieved upon small molecule treatment or proven reversible genetic inhibition (e.g. using lox/frt pairs). However, as presented the data simply do not support the rather strong claim of the authors that "All together, these data underline the reversibility of Trf1 depletion and support that a transient ubiquitous Trf1 downregulation is compatible with mouse viability".

We think that our data obtained by genetically ablating TRF1 strongly supports the applicability of a therapeutical strategy based on TRF1-pharmacological inhibition. Indeed, and as the reviewer points out, genetic ablation is irreversible in those cells that excised the given gene, while it is not the case for chemical inhibition. With our results, we show that transient TRF1 depletion in adult organism regardless the way it is achieved, does not compromise mouse

4th Editorial Decision 11 September 2015

Thank you for your letter and rebuttal to Reviewer 1's evaluation, and again apologies for the delay, due to concurrent travel.

Before I deal with the specific issue at hand, please allow me to clarify a few points you touched upon in your previous communications:

1) Your revised manuscript was sent out to the same three reviewers that had evaluated your first version. Since it was technically a de novo submission, the order of the reviewers was assigned randomly, thus generating some confusion. I apologise for this but also assure you that you were shown all the comments received. In essence, there was simply a numbering switch between Reviewers 2 and 3.
2) I specifically mentioned in my decision letter that we chose not to consider the comments from Reviewer 2 (formerly Reviewer 3).

That said, we do understand your request to reconsider our initial decision and thank you for providing further commentary and information. I have now re-discussed your manuscript, the Reviewer evaluations and your letter without prejudice, including with my colleagues. I am sorry to write that we have decided to uphold our original decision.

We are of the opinion that the issues raised by Reviewer 1 (who is very expert and respected in the community and has always provided consistent, constructive and fair counsel and evaluations) remain valid and unresolved.

I should also reiterate that clearly, Reviewer 3 (formerly Reviewer 2) is not satisfied that the human data provided are of sufficient quality and generally adequate to sustain a proof of principle in human. Although admittedly s/he appears willing to overlook this, we do not agree and feel that instead to show the relevance of the findings in human would help establish TRF1 as a therapeutic target in human cancer.

We appreciate that you are not ready yet to include data on small molecule inhibitors of TRF1. I'm sure you understand, however, that this type of proof of concept would completely reverse the situation and lend strong validity and interest to your findings.

Finally, although we understand that there is always room for debate, we must ultimately make an editorial decision, which in this case is that unfortunately we cannot offer to publish your manuscript.

Additional author correspondence 17 September 2015

I do not know whether you had a chance to read my email below. We would be interested in discussing with you the possibility of adding additional data on the small molecules that target TRF1. In particular, for us it will be important to know which data would you request for further considering the manuscript at EMBO Molecular Medicine.

The experiments that we have already performed and could be added immediately to the manuscript are the following:

1) Design and validation of the screening (see attached Figure for the reviewers that we also provided before to you and the reviewers)
2) Demonstration that the compounds do not affect TRF1 transcription and protein stability.
3) Cellular assays demonstrating inhibition of TRF1 binding to telomeres by immunofluorescence both in IPS cells and lung carcinoma cell lines upon treatment with the compounds.
4) Cellular assays demonstrating induction of DNA damage in iPS cells and lung carcinoma cell lines upon treatment with the compounds.

Of course, we could consider your suggestions on this issue.

I look forward to hearing from you

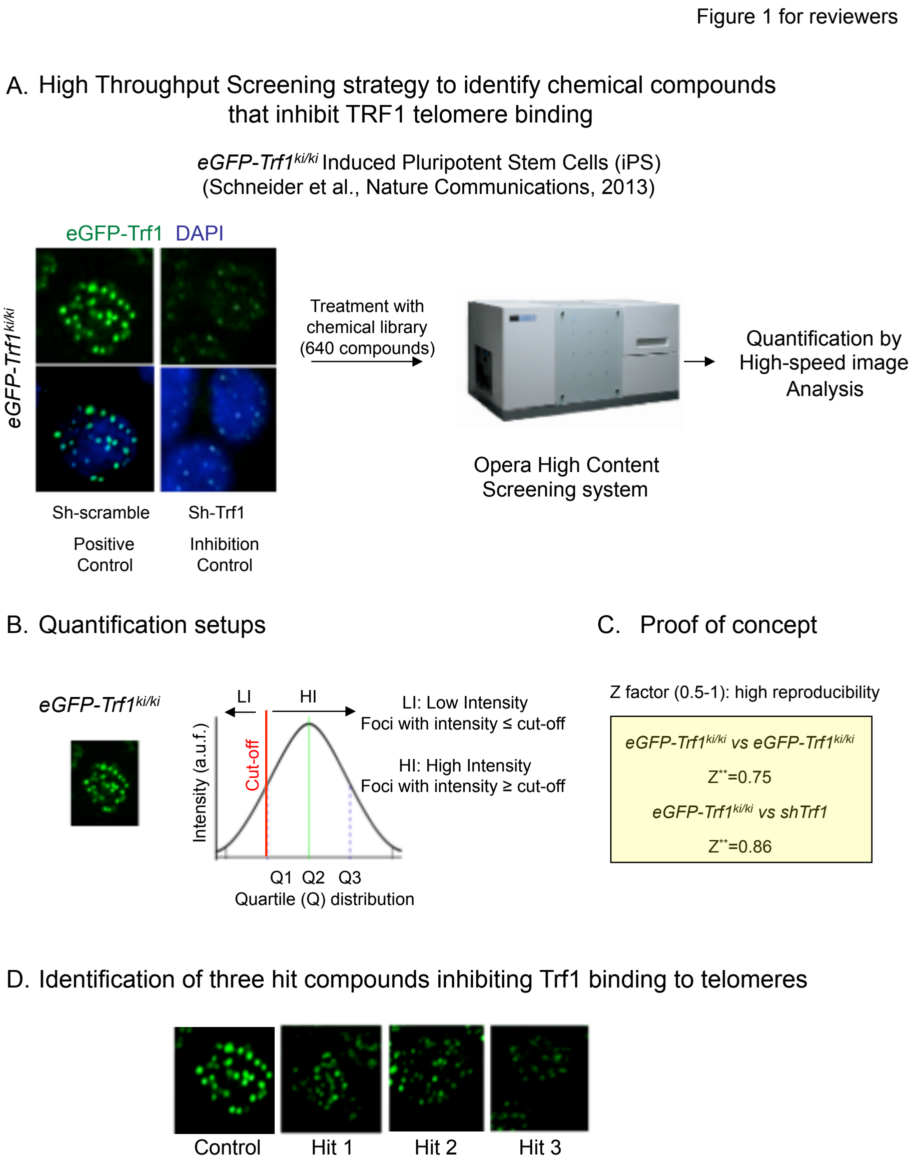

Additional editorial correspondence 24 September 2015

Thank you again for your note and apologies for the delay in getting back to you. We have been busy with many new submissions that, I hope you will understand, must take precedence over appeals and requests to reconsider editorial decisions.

We very much appreciate that you shared your data in progress on the potential TRF1 inhibitors, which appear quite promising.

As I mentioned in my last letter, provision of a proof of concept showing the effects of systemic Trf1 depletion on healthy tissues with a small molecule treatment or proven reversible genetic

inhibition, would lend strong translational validity to your findings including the import claim of reversibility; this is in fact, what appears to be really missing at this stage.

In essence, you would need to show this in vivo with your compound(s). Of course I understand that you may not have such data at present and I am sorry that I cannot be more positive at this stage. Nevertheless, if in the near future you should obtain this evidence, I would be more than happy to rediscuss your work.

3rd Appeal 21 October 2015

I am contacting you again regarding our manuscript entitled "TRF1 abrogation effectively impairs K-RasG12V-induced lung carcinogenesis" which was recently considered by you at EMBO Mol Med.
As we discussed by email, in parallel to the validation of TRF1 as a potential new anti-cancer target by using genetically modified mouse models, and given the very promising results obtained by TRF1 genetic deletion in blocking lung carcinogenesis in the context of the K-RasG12V p53-null lung cancer model, we have been running a drug development program at CNIO to screen for drugs that disrupt TRF1 binding to telomeres. We have now finished a set of experiments, which we think address some of the issues previously raised by you and the reviewers. In particular, we could include in a revised manuscript the following data:

1) In collaboration with the Experimental Therapeutics Program at CNIO, we have already identified chemical compounds, which disrupt TRF1 binding to telomeres in cellular assays. These compounds have been identified by screening compound libraries in a high throughput imaging platform by using cells derived from a eGFP-TRF1 reporter mouse generated by us, which allow easy follow up and quantification of TRF1 intensity at telomeres (Schneider et al., Nature Communications, 2013). New Supplementary Figure 4 shows the design of the screening and the identification of several hit compounds that decrease TRF1 binding to telomeres. These findings are proof of concept that it is possible to chemically target the binding of Trf1 to telomeres.

2) From the different hit compounds obtained, we have selected compounds ETP-47228 and ETP-47037 for further characterization. Treatment of lung adenocarcinoma cell lines (see new Figure 9) and iPS cells (new Supplementary Figure 5) with these compounds, leads to decreased TRF1 amounts at telomeres, induction of damage, induction of telomere damage foci or TIFs, and decreased proliferation (see new Figure 9). These findings show that treatment with these compounds leads to telomere "uncapping" similar to that demonstrated by TRF1 genetic depletion in the context of the lung cancer mouse model, thus supporting that these compounds are disrupting TRF1 binding to telomeres.

3) To assess the effects of TRF1 inhibitors on lung carcinomas in vivo, we studied whether they could inhibit the growth of already established K-RasG12V lung carcinomas also lacking p53. To this end, have selected compound ETP-47037 owing to the fact that it has good in vivo properties. Thus, we previously determined that ETP-47037 is an orally bioavailable compound and has good pharmacokinetics. Strikingly, 10 days of treatment with this compound effectively impairs the progression of already formed lung carcinomas compared to the group treated with vehicle (see new Figure 10). Importantly, during the time of treatment the mice showed a normal viability and did not show any signs of sickness. Of note, compound ETP-47037 was also able to significantly impair the growth of xenographs of lung adenocarcinoma cell lines treated with the compound (see new Figure 9E) These new findings indicate that TRF1 inhibition can be achieved in vivo using chemical compounds and that there is a therapeutic window for targeting Trf1 in cancer.

Altogether, our findings support the conclusion that induction of acute telomere uncapping emerges as a potential new therapeutic target for lung cancer.

We hope that you consider that a revised manuscript including all the new data indicated above (see the attached new figures) could of interest for EMBO Mol Med. I will be able to send the revised manuscript within the next few days.

Please let us know as soon as possible and thank you for your editorship.

5th Editorial Decision 30 October 2015

We have now re-discussed your manuscript and your new data as per your October 21st, 2014 letter. We agree that this appears to be a significant improvement along the lines suggested and I can therefore commit to sending it out for a new evaluation. I will not be sending the revised maunscipt to Reviewer 2 (formerly Reviewer 3).

I look forward to seeing a revised form of your manuscript as soon as possible.

1st Revision - authors' response 14 January 2015

I am contacting you again regarding our fully revised manuscript now entitled "Therapeutic inhibition of TRF1 impairs the growth of p53-deficient K-RasG12V-induced lung cancer by induction of telomeric DNA damage" which was previously considered by you at EMBO Mol Med.

First, sorry for the delayed submission but to fully answer reviewer #1 we decided to perform a detailed analysis and follow up mice with TRF1 deletion in the whole organism for up to 4 months to rule out long term toxicity (see new Figure 6). This has considerably delayed submission of the revised manuscript.

As we discussed by email, in parallel to the validation of TRF1 as a potential new anti-cancer target by using genetically modified mouse models, and given the very promising results obtained by TRF1 genetic deletion in blocking lung carcinogenesis in the context of the K-RasG12V p53-null lung cancer model, we have been running a drug development program at CNIO to screen for drugs that disrupt TRF1 binding to telomeres. We have now finished a new set of experiments, which we think address some of the issues previously raised by you and the reviewers. In particular, we have included in a revised manuscript the following data:

1) In collaboration with the *Experimental Therapeutics Program* at CNIO, we have identified chemical compounds, which disrupt TRF1 binding to telomeres in cellular assays. These compounds have been identified by screening compound libraries in a high throughput imaging platform by using cells derived from a eGFP-TRF1 reporter mouse generated by us, which allow easy follow up and quantification of TRF1 intensity at telomeres (Schneider et al., *Nature Communications*, 2013). **New Supplementary Figure 7** shows the design of the screening and the identification of several hit compounds that decrease TRF1 binding to telomeres (see also **new Figure 7 and new Supplementary Figure 9**). These findings are proof of concept that it is possible to chemically target the binding of Trf1 to telomeres.

2) From the different hit compounds obtained, we have selected compounds ETP-47228 and ETP-47037 for further characterization. Treatment of lung adenocarcinoma cell lines (**see new Figure 7**) and iPS cells (**new Supplementary Figure 9**) with these compounds, leads to decreased TRF1 amounts at telomeres, induction of damage, induction of telomere damage foci or TIFs, and decreased proliferation (**see new Figure 7**). These findings show that treatment with these compounds leads to telomere "uncapping" similar to that demonstrated by TRF1 genetic depletion in the context of the lung cancer mouse model, thus supporting that these compounds are disrupting TRF1 binding to telomeres.

3) To assess the effects of TRF1 inhibitors on lung carcinomas *in vivo*, we studied whether they

could inhibit the growth of already established *K-Ras*^{G12V} lung carcinomas also lacking p53. To this end, have selected compound ETP-47037 owing to the fact that it has good in vivo properties (**see new Supplementary Figure 8**). Thus, we previously determined that ETP-47037 is an orally bioavailable compound and has good pharmacokinetics. Strikingly, 10 days of treatment with this compound effectively impairs the progression of already formed lung carcinomas compared to the group treated with vehicle (**see new Figure 8**). Lung carcinomas treated with the compound show decreased TRF1, decreased proliferation, and increased G2 arrest, recapitulating the same phenotypes as TRF1 genetic deletion and thus demonstrating effective targeting of TRF1 in vivo (**new Figure 8**). Importantly, during the time of treatment the mice showed a normal viability and did not show any signs of sickness or severe tissue abnormalities (**new Figure 8**). Of note, compound ETP-47037 was also able to significantly impair the growth of xenographs of lung adenocarcinoma cell lines treated with the compound (**see new Figure 7e**) These new findings indicate that TRF1 inhibition can be achieved in vivo using chemical compounds and that there is a therapeutic window for targeting Trf1 in cancer.

Altogether, our findings support the conclusion that induction of acute telomere uncapping emerges as a potential new therapeutic target for lung cancer.

We hope that you consider that the revised manuscript is now of interest for *EMBO Mol Med*.

We look forward to hearing from you as soon as possible to avoid delays in the publication of the paper and thank you for your editorship.

Detailed Answer to reviewer #1

Referee #1 (Remarks):

Overall the manuscript has been much improved. Unfortunately, there is still a major issue with the MS, that arises as a consequence of the additional experiments performed. The question whether temporal Trf1 depletion will be detrimental for normal tissue homeostasis remains unanswered even though the authors claim the opposite.

ANSWER: We thank the reviewer for indicating that **"the manuscript has been much improved**" and would like also to thank her/him for the detailed revision of our manuscript. Regarding the reviewer concern that temporal TRF1 depletion will be detrimetral for normal tissue homeostasis, we agree with the reviewer and for that reason have now extended the TRF1 genetic deletion experiments to up to 7 months to rule out severe long term toxicity (see **new Figure 6**).

In particular:

1. We now show in the revised manuscript that mice in which TRF1 has been systemically deleted by TMX addition to de diet during 7 weeks recover normal blood counts 3 weeks after removal of TMX and these normal blood counts are maintained up to 4 months after TMX removal (see **new Figure 6f**).

2.- We have now deleted TRF1 by TMX addition to the diet for more than 6 months and the mice show sustained TRF1 abrogation and no overt signs of pathologies in the tissues studied.

3. – We have now included in the revised manuscript in vivo data on the treatment of lung carcinomas with drugs that disrupt TRF1 binding to telomeres. In particular, **new Supplementary Figure 7** shows the design of the screening and the identification of several hit compounds that decrease TRF1 binding to telomeres (see also **new Figure 7 and new Supplementary Figure 9**). From the different hit compounds obtained, we have selected compounds ETP-47228 and ETP-47037 for further characterization. Treatment of lung adenocarcinoma cell lines (**see new Figure 7**) and iPS cells (**new Supplementary Figure 9**) with these compounds, leads to decreased TRF1 amounts at telomeres, induction of damage, induction of telomere damage foci or TIFs, and decreased proliferation (**see new Figure 7**). To assess the effects of TRF1 inhibitors on lung carcinomas *in vivo*, we studied whether they could inhibit the growth of already established *K-*

Ras^{G12V} lung carcinomas also lacking p53, **without generating severe toxicities**. To this end, we have selected compound ETP-47037 owing to the fact that it has good in vivo properties (**see new Supplementary Figure 8**). Thus, we previously determined that ETP-47037 is an orally bioavailable compound and has good pharmacokinetics. Strikingly, 10 days of treatment with this compound effectively impairs the progression of already formed lung carcinomas compared to the group treated with vehicle (**see new Figure 8**). Lung carcinomas treated with the compound show decreased TRF1, decreased proliferation, and increased G2 arrest, recapitulating the same phenotypes as TRF1 genetic deletion and thus demonstrating effective targeting of TRF1 in vivo (**new Figure 8**). **Importantly, during the time of treatment the mice showed a normal viability and did not show any signs of sickness or severe tissue abnormalities** (**new Figure 8**). Of note, compound ETP-47037 was also able to significantly impair the growth of xenographs of lung adenocarcinoma cell lines treated with the compound (**see new Figure 7e**) These new findings indicate that TRF1 inhibition can be achieved in vivo using chemical compounds and that there is a therapeutic window for targeting TRF1 in cancer.

Indeed the short-term effects of tamoxifen-induced genetic deletion of Trf1 in all tissues are limited as one might expect for cells that do not divide.

ANSWER: As mentioned above, the revised manuscript now includes long-term follow up of TRF1 genetic deletion upon tamoxifen treatment (see **new Figure 6**). It is also important to point out that TRF1 genetic deletion is effective not only in non-proliferating tissues (heart, lung, liver, kidney, brain) but also in highly proliferative ones such as intestine, skin, blood and bone marrow for up to six months (see **new Figure 6**).

However, in the longer-term analysis of normal tissues the authors show that Trf1 levels - after initial depletion upon tamoxifen treatment- quickly recover (some additional information would be useful here: how the levels over time after cessation of the tamoxifen treatment compare with levels of animals not carrying a Trf1 floxed alleles).

ANSWER: We now include TRF1 expression levels in wild-type animals (see **new Figure 6g**). We have now followed mice treated with TMX for 7 weeks up to 4 months after TMX removal observing sustained recovery of the blood counts (see **new Figure 6f**).

This implies that the cells that have actually retained full Trf1 function during tamoxifen treatment (failed to switch) are the ones that start to reconstitute the normal tissues. Therefore, it remains unclear whether tissue stem cells would be capable to re-gain their normal function after temporal Trf1 depletion since in the experiment shown the ones that actually are reconstituting the tissue never have suffered from Trf1 depletion.

ANSWER: We are fully aware that those cells that have excised TRF1 alleles by genetic means are not able to reconstitute the tissues. As the reviewer points out and as we state in the manuscript, those stem cells that escaped TRF1 deletion are the ones regenerating tissues, especially those with high turn-over such blood, intestine, skin and bone marrow (see **new Figure 6**).

The authors seem not to have realized that there is an important conceptional difference between pharmacologic inhibition and incomplete genetic ablation in that the cells that repopulate the normal tissues never have suffered Trf1 depletion since the gene was not deleted in those cells (This is also indicated by the higher percentage of Trf1 positive cells in the crypts of the intestine (fig 8C)). In that regard these cells are reminiscent of the tumor escapers seen in the p53 proficient tumors.

ANSWER: As mentioned above, we have now included extensive new experimentation showing chemical inhibition of TRF1 in vivo. In particular, in collaboration with the *Experimental Therapeutics Program* at CNIO, we have identified chemical compounds, which disrupt TRF1 binding to telomeres in cellular assays. These compounds have been identified by screening compound libraries in a high throughput imaging platform by using cells derived from a eGFP-TRF1 reporter mouse generated by us, which allow easy follow up and quantification of TRF1 intensity at telomeres (Schneider et al., *Nature Communications*, 2013). **New Supplementary Figure 7** shows the design of the screening and the identification of several hit compounds that decrease TRF1 binding to telomeres (see also **new Figure 7 and new Supplementary Figure 9**).

These findings are proof of concept that it is possible to chemically target the binding of Trf1 to telomeres. From the different hit compounds obtained, we have selected compounds ETP-47228 and ETP-47037 for further characterization. Treatment of lung adenocarcinoma cell lines (**see new Figure 7**) and iPS cells (**new Supplementary Figure 9**) with these compounds, leads to decreased TRF1 amounts at telomeres, induction of damage, induction of telomere damage foci or TIFs, and decreased proliferation (**see new Figure 7**). These findings show that treatment with these compounds leads to telomere "uncapping" similar to that demonstrated by TRF1 genetic depletion in the context of the lung cancer mouse model, thus supporting that these compounds are disrupting TRF1 binding to telomeres. To assess the effects of TRF1 inhibitors on lung carcinomas *in vivo*, we studied whether they could inhibit the growth of already established *K-Ras*^{G12V} lung carcinomas also lacking p53, **without generating severe toxicities**. To this end, have selected compound ETP-47037 owing to the fact that it has good in vivo properties (**see new Supplementary Figure 8**). Thus, we previously determined that ETP-47037 is an orally bioavailable compound and has good pharmacokinetics. Strikingly, 10 days of treatment with this compound effectively impairs the progression of already formed lung carcinomas compared to the group treated with vehicle (**see new Figure 8**). Lung carcinomas treated with the compound show decreased TRF1, decreased proliferation, and increased G2 arrest, recapitulating the same phenotypes as TRF1 genetic deletion and thus demonstrating effective targeting of TRF1 in vivo (**new Figure 8**). **Importantly, during the time of treatment the mice showed a normal viability and did not show any signs of sickness or severe tissue abnormalities** (**new Figure 8**). Of note, compound ETP-47037 was also able to significantly impair the growth of xenographs of lung adenocarcinoma cell lines treated with the compound (**see new Figure 7e**) These new findings indicate that TRF1 inhibition can be achieved in vivo using chemical compounds and that there is a therapeutic window for targeting Trf1 in cancer.

Therefore, the question of the effect of temporal Trf1 inhibition has simply not been answered in this in vivo setting. The notion that in p53-/- tumors only 5% of the escapers have retained a functional Trf1 allele suggests that the therapeutic index of this strategy could be actually quite low since the tumors apparently have ways to tolerate Trf1 depletion that are not accessible to normal p53-proficient cells.

ANSWER: we think that the reviewer may have misunderstood this result. Our data shows that p53-negative cells are more tolerable to TRF1-loss than p53-proficient ones. However, we clearly show that tumor development, growth and progression to malignity are strongly compromised in p53-deficient cells lacking TRF1 as compared to TRF1-proficient cells both by TRF1 genetic deletion and by TRF1 chemical inhibition.

The results the authors show in fig 8G would be great -and would support their conclusion of reversibility- if they were achieved upon small molecule treatment or proven reversible genetic inhibition (e.g. using lox/frt pairs). However, as presented the data simply do not support the rather strong claim of the authors that "All together, these data underline the reversibility of Trf1 depletion and support that a transient ubiquitous Trf1 downregulation is compatible with mouse viability".

ANSWER: As extensively mentioned above, we have now included extensive new experimentation showing that TRF1 chemical inhibition is effective in targeting TRF1 in vivo without significant effects on tissue toxicity.

Detailed Answer to reviewer #3

Referee #3 (Remarks):

While the mouse work was excellently complemented and extended by the conditional whole-body TRF1 deletion in adult mice and was obviously already prepared for resubmission, the work on the human cells appears to be initiated de novo and unfortunately remains spartan. The need for a proof of principle to test the hypothesis also for human cells is now prominently expressed in the Abstract and Statement of Significance. Unfortunately, the data behind this statement are very preliminary thereby missing the purpose of this question -namely the difference in regulation of telomerase and telomere length in human versus mouse.

© EMBO

ANSWER: We thank the reviewer for considering that the mouse work has been **"excellently complemented and extended by the conditional whole-body TRF1 deletion in adult mice**" and for her/his thorough revision and insightful commentaries. We also understand the reviewer concerns regarding the cell line data. In the revised manuscript, we have now extended the cell line work by addition of extensive new experimentation with chemical compounds that are able to target TRF1. We hope that the new experimentation with chemical compounds both using the genetically modified mouse lung cancer model and cell lines (both cancer cell lines and iPS cells) will now justify retaining the cell line data in the manuscript.

In particular:

1) In collaboration with the *Experimental Therapeutics Program* at CNIO, we have identified chemical compounds, which disrupt TRF1 binding to telomeres in cellular assays. These compounds have been identified by screening compound libraries in a high throughput imaging platform by using cells derived from a eGFP-TRF1 reporter mouse generated by us, which allow easy follow up and quantification of TRF1 intensity at telomeres (Schneider et al., *Nature Communications*, 2013). **New Supplementary Figure 7** shows the design of the screening and the identification of several hit compounds that decrease TRF1 binding to telomeres (see also **new Figure 7 and new Supplementary Figure 9**). These findings are proof of concept that it is possible to chemically target the binding of Trf1 to telomeres.

2) From the different hit compounds obtained, we have selected compounds ETP-47228 and ETP-47037 for further characterization. Treatment of lung adenocarcinoma cell lines (**see new Figure 7**) and iPS cells (**new Supplementary Figure 9**) with these compounds, leads to decreased TRF1 amounts at telomeres, induction of damage, induction of telomere damage foci or TIFs, and decreased proliferation (**see new Figure 7**). These findings show that treatment with these compounds leads to telomere "uncapping" similar to that demonstrated by TRF1 genetic depletion in the context of the lung cancer mouse model, thus supporting that these compounds are disrupting TRF1 binding to telomeres.

3) To assess the effects of TRF1 inhibitors on lung carcinomas *in vivo*, we studied whether they could inhibit the growth of already established *K-Ras*^{G12V} lung carcinomas also lacking p53. To this end, have selected compound ETP-47037 owing to the fact that it has good in vivo properties (**see new Supplementary Figure 8**). Thus, we previously determined that ETP-47037 is an orally bioavailable compound and has good pharmacokinetics. Strikingly, 10 days of treatment with this compound effectively impairs the progression of already formed lung carcinomas compared to the group treated with vehicle (**see new Figure 8**). Lung carcinomas treated with the compound show decreased TRF1, decreased proliferation, and increased G2 arrest, recapitulating the same phenotypes as TRF1 genetic deletion and thus demonstrating effective targeting of TRF1 in vivo (**new Figure 8**). Importantly, during the time of treatment the mice showed a normal viability and did not show any signs of sickness or severe tissue abnormalities (**new Figure 8**). Of note, compound ETP-47037 was also able to significantly impair the growth of xenographs of lung adenocarcinoma cell lines treated with the compound (**see new Figure 7e**) These new findings indicate that TRF1 inhibition can be achieved in vivo using chemical compounds and that there is a therapeutic window for targeting Trf1 in cancer.

Not cell lines but a single cell line was used, the A549 cell line, without referring to the relevance of these particular cells. A549 cells infected with shTRF1 showed significant but not total reduction of TRF1 expression. How did this interfere with growth in culture?

ANSWER: The A549 cell line was derived from a human lung carcinoma, in particular from alveolar basal epithelia cells. TRF1 downregulation slowed down the growth of these cells although we did not observe increased cell death.

The cells were injected s.c. and a graph is presented monitoring tumor growth. The only information provided is that after a lag phase of 17 days 2 tumors developed within the 8 injection sites. Unfortunately, no histology, no molecular characterization and mechanistic reason for tumor suppression are provided, nor is a description for the late escape of the tumor cells. As this is meant

to prove the comparability of the human system and with that its potential for application in humans, these data are insufficient and do not legitimate the statement "Downregulation of TRF1 in cell lines derived from already established p53-deicient K-RAS lung carcinomas also impaired tumor growth and metastasis in allograft models, as well as in xenograph models of human lung cancer cell lines".

ANSWER: As mentioned above, we have now included new data with TRF1 chemical inhibition using cell lines. We agree with the reviewer, however, that is best to remove the above statement from the Abstract and we have done so in the revised manuscript.

As the mouse work is well justified and very well done the authors may reconsider to include the at present unsatisfactory work on human cells and instead point to the fact that future studies with human cells have to show the relevance of these finding also for the human system - as this is crucial for proposing TRF1 as a therapeutic target for human lung cancer.

ANSWER: We think that inclusion of additional work with both mouse and human cancer cell lines treated with chemical compounds that target TRF1 may justify not removing the TRF1 knock-down data, but we would be happy to do so if considered appropriate by the Editor.

6th Editorial Decision 13 March 2015

Thank you for the submission of your manuscript to EMBO Molecular Medicine. We have now heard back from two Reviewers whom we asked to evaluate your manuscript.

We are sorry that it has taken an unusually long time to get back to you on your manuscript. In this case we experienced unusual difficulties in securing evaluations from the two reviewers and obtaining them in a timely manner and furthermore, one Reviewer eventually did not deliver.

Regardless I feel I have enough feedback to be able to proceed on your manuscript and as you will see, the Reviewer, while raising inevitable concerns deriving from the substantial new experimentation, is globally positive. I will not dwell into much detail, as the Reviewers' comments are clear. I would like, however, to highlight a few main points.

The Reviewer asks you to clarify a number of points on the cell lines, to not dismiss as minor the side effects of TRF1 depletion, and in general to tone-down your conclusions. S/he also wishes to see a better and more careful illustration of experimental details. The reviewer also lists many other pertinent and well-taken points for improvement in experimental quality, explanation and presentation that I would strongly suggest you to take action on to improve your manuscript

We would thus be pleased to consider a revised submission, with the understanding that the Reviewer's concerns must be addressed with additional experimental data where appropriate and that acceptance of the manuscript might entail another (rapid) round of review.

Please note that it is EMBO Molecular Medicine policy to allow a single round of revision only and that, therefore, acceptance or rejection of the manuscript will depend on the completeness of your responses included in the next, final version of the manuscript.

As you know, EMBO Molecular Medicine has a "scooping protection" policy, whereby similar findings that are published by others during review or revision are not a criterion for rejection. However, I do ask you to get in touch with us after three months (which I sincerely hope will not be necessary) if you have not completed your revision, to update us on the status. Please also contact us as soon as possible if similar work is published elsewhere.

I look forward to seeing a revised form of your manuscript as soon as possible.

***** Reviewer's comments *****

Referee #3 (Comments on Novelty/Model System):

As stated in the comments, some more specific informations - that are present somewhere - would help to follow the work if being present in the main body of the text.

Referee #3 (Remarks):

The authors have performed a completely new set of experiments in order to demonstrate that TRF1 is drug targetable and that this is similarly effective for lung cancer therapy as genetic TRF1 depletion. This is clearly an important and interesting piece of work, however, also initiates a new discussion.

1. for clarification only: As taken from the rebuttle letter:

Not cell lines but a single cell line was used, the A549 cell line, without referring to the relevance of these particular cells. A549 cells infected with shTRF1 showed significant but not total reduction of TRF1 expression. How did this interfere with growth in culture?
ANSWER: The A549 cell line was derived from a human lung carcinoma, in particular from alveolar basal epithelia cells. TRF1 downregulation slowed down the growth of these cells although we did not observe increased cell death.

In days of high number of misidentified human cell lines, this information as well as the status of the cells needs to be included in the manuscript. - However, the point made here was not well taken. It is highly important to prove that human tumor cells respond accordingly and the work performed with the A549 cells is well done. As human tumors and accordingly cell lines derived therefrom are genetically highly heterogeneous, using one cell line can only be a proof of principle and should be clearly stated as such. Statements such as "additional work with both mouse and human cancer cell lines" (see below and also in the manuscript) pretend an as yet unjustified generalization.

2. A second point also arising out of the generalization/globalization is the emphasis that mice do not show loss of viability by whole body TRF1 deletion or compound treatment. As would be expected, if correlated with acute health problems, the authors would not have pursued TRF1 depletion. However, the authors describe defined side effects in the rapidly proliferating tissues such as reduced cellularity of the intestine and epidermis of the skin, or even hyperplasia, hair follicle and sebaceous gland abnormalities. This is severe and argues for cell death of stem cells, thus should not be dismissed as only minor.

With their new data - compound treatment in vivo - the authors point out again that during the time of treatment (8 days) "the mice showed a normal viability and did not show any signs of thickness. Nevertheless they describe a panel of side effects (intestine, skin, bone marrow) less severe than upon genetic TRF1 depletion, however, clearly visible within only 8 days of treatment.

That said it is difficult to share the enthusiasm of the authors. As shown in the new Fig. 8, the CT scan (Fig. 8b right) demonstrates that a 10 day treatment with ETP-47037, at least in this lung, allowed for the increase in nodule size as well as the de novo appearance of a tumor nodule. If this is a representative CT image, it is difficult to understand how this correlates with the corresponding graph (Fig. 8b left). In addition to the side effects demonstrated upon treatment one may be careful in stating that "these findings indicate that TRF1 inhibition can be achieved in vivo using chemical compounds and that there is a therapeutic window for targeting TRF1 in cancer". In general, one may wish for a less intrusive but more deliberative presentation and interpretation.

Some minor technical aspects

- The authors would very much help for easier reading by specify the cells, compound concentrations etc. used for the particular experiments when describing the results.

- The authors use different antibodies to demonstrate the telomeres. It may be wished to see TRF1 and telomere co-staining (in situ) in order to demonstrate true depletion of TRF1 from the telomeres and to exclude staining artefacts.

- Suppl Fig. 9c is surprising as no H2AX foci are seen in control but nearly every cell is positive upon treatment. Nevertheless intensity increases from 1(?) to 1.5 only. Accordingly, TIFs are present in about 8% of control and 24% of treated cells. Shouldn't this already be reflected in Fig. 9c? Is fig. 9d (outer right) indeed a representative figure/cell?

- Why was 10µM selected for the TRF1 and H2AX staining while 10 µM was obviously not tested for toxicity - but 2 µM were already toxic.

- Fig 7. The figure quality of 7b DMSO control needs to be improved. It also would be helpful to present the same magnifications in Fig. 7c. One may worry that the RAP1 + H2AX staining for ETP-47037 does represent an S phase cell - as indicated by the high amount of TRF1 foci and typical high amount of H2AX.

- Why are ETP-47228 data shown for 24h and ETP-47037 for 48h.

- Unfortunately a dose response curve was only performed for proliferation - with a cell line not further specified. As this compound may be further pursued as potential "clinical" candidate compound, evaluation of the analyzed concentrations remains unclear.

- What means pretreatment (for how long) of the tumor cells for generating the allograft model? And please indicate whether or not the cells were further treated in vivo.

- Having experienced smaller tumors (higher tumor diameter fold change) upon EPT-47228 treatment, this would have been a favorable compound to test in vivo. If however, the in vivo pharmacokinetics and the bio-availability is poor, why was this compound chosen for the other studies.

- Fig. 8: What do the authors mean with "EPT-47037 effectively impaired tumor progression". The CT scan may be unfavorable but as presented does not support this statement. Would a longer treatment show a more clear indication for tumor reduction and/or stagnation?

- (Fig. 8c): the TRF1 content is already extremely low in the controls and reflects more the pattern for H2AX foci. Could this be improved?

- Finally, the "home made" TRF1 polyclonal rabbit antibody may be checked. In skin it exhibits a high background, which strongly reflects cytoskeleton. As rabbits frequently carry cytokeratin autoantibodies this may be investigated and seen in the pre-immune serum.

2nd Revision - authors' response 20 March 2015

EMBO MOLECULAR MEDICINE EMM-2014-04497-V4

Detailed Answer to Referee #3

[AUTHORS] We would like to start this point by point response letter to the reviewer's suggestions by thanking she/her for the careful examination of our manuscript and for their interest in our work. We strongly believe that all the raised points are useful and will help to improve the final manuscript.

We would like to particularly thank the reviewer for the following statement:

[Reviewer #3] "The authors have performed a completely new set of experiments in order to demonstrate that TRF1 is drug targetable and that this is similarly effective for lung cancer therapy as genetic TRF1 depletion. This is clearly an important and interesting piece of work".

Referee #3 (Comments on Novelty/Model System):

As stated in the comments, some more specific informations - that are present somewhere - would help to follow the work if being present in the main body of the text.

Referee #3 (Remarks):

The authors have performed a completely new set of experiments in order to demonstrate that TRF1 is drug targetable and that this is similarly effective for lung cancer therapy as genetic TRF1 depletion. This is clearly an important and interesting piece of work, however, also initiates a new discussion.

[Reviewer] 1. for clarification only: As taken from the rebuttal letter: Not cell lines but a single cell line was used, the A549 cell line, without referring to the relevance of these particular cells. A549 cells infected with shTRF1 showed significant but not total reduction of TRF1 expression. How did this interfere with growth in culture?
ANSWER: The A549 cell line was derived from a human lung carcinoma, in particular from alveolar basal epithelia cells. TRF1 downregulation slowed down the growth of these cells although we did not observe increased cell death.
In days of high number of misidentified human cell lines, this information as well as the status of the cells needs to be included in the manuscript. - However, the point made here was not well taken. It is highly important to prove that human tumor cells respond accordingly and the work performed with the A549 cells is well done. As human tumors and accordingly cell lines derived therefrom are genetically highly heterogeneous, using one cell line can only be a proof of principle and should be clearly stated as such. Statements such as "additional work with both mouse and human cancer cell lines" (see below and also in the manuscript) pretend an as yet unjustified generalization.

[AUTHORS] We agree with the reviewer and have modified some of the conclusions that we made in the manuscript: **"Thus, Trf1 downregulation blocks the growth of cell lines derived from already formed lung tumors both in mice and humans"**

has been now replaced by **"Thus, Trf1 downregulation blocks the growth of cell lines derived from already formed lung mouse tumors, and has proven efficacy in one human cancer cell line"** (page 13, 1st paragraph).

[Reviewer] 2. A second point also arising out of the generalization/globalization is the emphasis that mice do not show loss of viability by whole body TRF1 deletion or compound treatment. As would be expected, if correlated with acute health problems, the authors would not have pursued TRF1 depletion. However, the authors describe defined side effects in the rapidly proliferating tissues such as reduced cellularity of the intestine and epidermis of the skin, or even hyperplasia, hair follicle and sebaceous gland abnormalities. This is severe and argues for cell death of stem cells, thus should not be dismissed as only minor.
With their new data - compound treatment in vivo - the authors point out again that during the time of treatment (8 days) "the mice showed a normal viability and did not show any signs of sickness". Nevertheless they describe a panel of side effects (intestine, skin, bone marrow) less severe than upon genetic TRF1 depletion, however, clearly visible within only 8 days of treatment.

[AUTHORS] We understand the reviewer's concern. Indeed, we have now increased the sample size of animals under tamoxifen diet analyzed for TRF1 mRNA expression levels from four to eight. We have now included the new data in **new Figure 6b**. In particular, deletion of *TRF1* upon tamoxifen diet reaches downregulation levels ranging from 50% to 90% depending on the tissue (**new Figure 6b**).

In addition, we agree with the reviewer that there are detectable side effects of Trf1 depletion/inhibition in rapidly proliferating tissues from some of the Trf1 downregulated mice, such as moderately decreased cellularity in the bone marrow and blood. However, the fact that mice show normal survival, suggest that these defects are not extremely severe. Nevertheless, we agree with the reviewer that these effects are likely to be due to effects on the stem cells. We have therefore rephrased this part of the results in the revised manuscript following the reviewers commentaries: **"Histopathological analysis of the tissues revealed that the *Trf1$^{\Delta/\Delta}$ hUBC-CreERT2* mice showed alterations in rapidly proliferating tissues consistent with the stem cell compartment being affected"** (page 14, 1st paragraph).

[Reviewer] That said it is difficult to share the enthusiasm of the authors. As shown in the new Fig. 8, the CT scan (Fig. 8b right) demonstrates that a 10 day treatment with ETP-47037, at least in this lung, allowed for the increase in nodule size as well as the de novo appearance of a tumor nodule. If this is a representative CT image, it is difficult to understand how this correlates with the corresponding graph (Fig. 8b left).

[AUTHORS] The reviewer is right that the representative images corresponding to ETP-47037 treated mice in the previous version of Fig. 8B can be improved. Indeed, we did not observe any de novo tumor formation during the ten days treatment with the TRF1 inhibitor. The nodule that seems to appear 10 days after treatment was already present showing a similar size in the CT-scan before treatment but we could not measure its exact borders due to presence of high inflammation surrounding the tumor. Given that we did not quantify this tumor at time 0, we initially did not include it in the representative image. However, we

appreciate the reviewers point and fully agree with him/her that we should also show the presence of this nodule to avoid misinterpretations. In the revised version of the figure, we now include a new representative image in which the tumor is visualized surrounded by tissue inflammation. This is also described in the revised text (page 18, 2nd paragraph). In addition, for the reviewer consideration, we include below (**Figure 1 for the reviewer**) a transversal CT-scan image in which the inflammation surrounding the tumor is readily observed. In summary, in the **revised Fig. 8**, now is clearly seen that short-term treatment with the inhibitor significantly slows down tumor growth in all nodules. Thus, the size of the tumors did not increase in any of the treated mice. In contrast, all the tumors grew significantly in the lungs of the vehicle treated mice.

[Reviewer] In addition to the side effects demonstrated upon treatment one may be careful in stating that "these findings indicate that TRF1 inhibition can be achieved in vivo using chemical compounds and that there is a therapeutic window for targeting TRF1 in cancer". In general, one may wish for a less intrusive but more deliberative presentation and interpretation.

[AUTHORS] We agree with the reviewer, and for that reason we have now have toned-down this statement in the revised manuscript: **"These findings indicate that TRF1 inhibition can be achieved *in vivo* using chemical compounds and that there is a therapeutic window for targeting TRF1 in cancer that merits further work "**(page 19, first paragraph)

Some minor technical aspects

[Reviewer] The authors would very much help for easier reading by specify the cells, compound concentrations etc. used for the particular experiments when describing the results.

[AUTHORS] We agree and we have introduced this information in the reviewed manuscript.

[Reviewer] The authors use different antibodies to demonstrate the telomeres. It may be wished to see TRF1 and telomere co-staining (in situ) in order to demonstrate true depletion of TRF1 from the telomeres and to exclude staining artefacts.

[AUTHORS] In order to show to the reviewer telomeric localization of the TRF1 signal, we have performed a double immunofluorescence staining with anti-TRF1 and anti-RAP1, another shelterin component, in intestine and skin sections of wild type animals (**Figure 2 for the reviewer**). Co-localization of TRF1 and RAP1 signals demonstrates telomeric localization.

[Reviewer] Suppl Fig. 9c is surprising as no γH2AX foci are seen in control but nearly every cell is positive upon treatment. Nevertheless intensity increases from 1.0 to 1.5 only. Accordingly, TIFs are present in about 8% of control and 24% of treated cells. Shouldn't this already be reflected in Fig. 9c? Is fig. 9d (outer right) indeed a representative figure/cell?

[AUTHORS] We understand the reviewers concern. The image in 9c was not of sufficient quality. In **revised Figure 9c**, we have now included a new representative image in which γH2AX foci in control cells are clearly visualized.

[Reviewer] Why does the increase in H2AX upon treatment with the inhibitor seem bigger in 9d compared to 9c?

[AUTHORS] Supplementary fig. 9c represents the total γH2AX fluorescence per nucleus genome wide while 9d represents the percentage of nuclei that have more than 3 γH2AX foci at telomeres. These results indicate that treatment with ETP-47228 treatment induces specifically more damage at telomeric DNA.

[Reviewer] Why was 10 μM selected for the TRF1 and γH2AX staining while 10 μM was obviously not tested for toxicity - but 2 μM were already toxic.

[AUTHORS] In addition to the dose response performed in the mouse lung tumor cell line (Fig. 7d), we also performed a dose response (DR) in iPS cells to analyze both proliferation and inhibitory effects on TRF1 levels (**Figure 3 for the reviewer**, Supplementary Figure 9e). The dose response assays started at 37.5μM and seven serials dilutions 1:2 were tested. At 10μM, both compounds showed an inhibitory effect, that for ETP-47037 was close to the EC50, while for ETP-47228 was around EC80. With regards to TRF1 inhibitory effects, at 2μM almost no effect was observed for either compound while at 10 μM both compound showed above 20% reduction in TRF1 levels **(Figure 3 for the reviewer).** Therefore, we select 10 μM for our assays to further characterize the inhibition of TRF1 foci levels in derived mouse lung tumor cell line.

[Reviewer] Fig 7. The figure quality of 7b DMSO control needs to be improved. It also would be helpful to present the same magnifications in Fig. 7c. One may worry that the RAP1 + γH2AX staining for ETP-47037 does represent an S phase cell - as indicated by the high amount of TRF1 foci and typical high amount of γH2AX.

[AUTHORS] We have included a new representative image for control DMSO treated cells in **revised Fig. 7b** and for the ETP-47037 treated cells in **revised Fig. 7c**. We do not include a lower magnification representative image for Fig 7c since for TIFs analysis we perform the confocal image capture with a higher magnification objective in order to have a higher resolution images and be able to clearly visualize the telomeric induced foci.

[Reviewer] Why are ETP-47228 data shown for 24h and ETP-47037 for 48h.

[AUTHORS] ETP-47228 reaches its maximum inhibitory activity at 24h, while ETP-47037 at 48h.

[Reviewer] Unfortunately a dose response curve was only performed for proliferation - with a cell line not further specified. As this compound may be further pursued as potential "clinical" candidate compound, evaluation of the analyzed concentrations remains unclear.

[AUTHORS] The cell line used for these studies is a mouse cell line derived from a mouse lung tumor of a *Kras*$^{+/G12V}$ *p53* $^{-/-}$ model. This is indicated in figure legend and in the text (page 17, 2nd paragraph). The proliferation assay started at 50μM, that is 4 times the concentration used in the screening (12.5μM), and eight serials dilutions 1:3 were tested. In addition to the effect on proliferation rates on IPS cells and in lung mouse tumor derived cell line, we also performed a doses response in iPS cells with regards to the levels of Trf1 inhibition (**Figure 3 for the reviewer**, Fig. 7d; Supplementary Fig. 9e). As stated above, based on the results obtained, we selected a concentration of 10μM that shows to have an inhibitory effect on TRF1.

[Reviewer] What means pretreatment (for how long) of the tumor cells for generating the allograft model? And please indicate whether or not the cells were further treated in vivo.

[AUTHORS] In the allograft model, the cells were treated in vitro for 48 hours with the inhibitors/DMSO and then injected in the nude mice, as indicated in Supplemental Materials and Methods (page 2, Allograft and Xenograft section). No further treatment with the inhibitors/DMSO was performed after the injections.

[Reviewer] Having experienced smaller tumors (higher tumor diameter fold change) upon EPT-47228 treatment, this would have been a favorable compound to test in vivo. If however, the in vivo pharmacokinetics and the bio-availability is poor, why was this compound chosen for the other studies.

[AUTHORS] Unfortunately, the ETP-47228 compound does not have good pharmacokinetics properties for in “in vivo” studies in mouse and for that reason we have focused our in vivo studies on the ETP-47037 compound. Nevertheless, we think it is important to show the in vitro effects of this compound in our manuscript as ETP-47228 was a clear hit in our “in vitro” screen.

[Reviewer] Fig. 8: What do the authors mean with "EPT-47037 effectively impaired tumor progression". The CT scan may be unfavorable but as presented does not support this statement. Would a longer treatment show a more clear indication for tumor reduction and/or stagnation?

[AUTHORS] In the **revised Fig. 8**, it is clearly seen that this short-term treatment with the inhibitor is enough to significantly slow down tumor growth. Thus, the size of the tumors did not increased in any of the treated animals. In contrast, in the vehicle treated lungs the all the tumors grew significantly. Of note, the results shown in Fig. 8 were obtained in mice with very large and fast growing p53-null lung tumors and even in this terminal cancer condition, the inhibitor was effective in blocking tumor growth. Future studies warrant testing of this inhibitor as well as derivatives of this inhibitor in blocking different tumor types in vivo.

[Reviewer] (Fig. 8c): the TRF1 content is already extremely low in the controls and reflects more the pattern for γH2AX foci. Could this be improved?

[AUTHORS] We do not understand the reasons underlying reviewer's claim that the TRF1 levels are extremely low in the control. The TRF1 levels in control samples correspond to wild type levels. The confocal settings for capturing and quantification of TRF1 fluorescence were set so that TRF1 was readily detected in control samples.

[Reviewer] Finally, the "home made" TRF1 polyclonal rabbit antibody may be checked. In skin it exhibits a high background, which strongly reflects cytoskeleton. As rabbits frequently carry cytokeratin autoantibodies this may be investigated and seen in the pre-immune serum.

[AUTHORS] We have widely used this antibody in our laboratory (Munoz et al., MCB, 2009; Martinez et al., G&D, 2009; Beier et al., Blood, 2012). In some tissues, it is a fact that this antibody shows citoplasmatic background. However, this is not interfering with the clean and clear TRF1 spot signal present in the nuclei, that can be quantified independently of the background. In addition, we show to the reviewer a double immunofluorescence staining with anti-TRF1 and anti-RAP1, another shelterin component, in intestine and skin sections of wild type animals (**Figure 2 for the reviewer**). Co-localization of TRF1 and RAP1 signals proves telomeric localization.

Figure 1 for reviewer

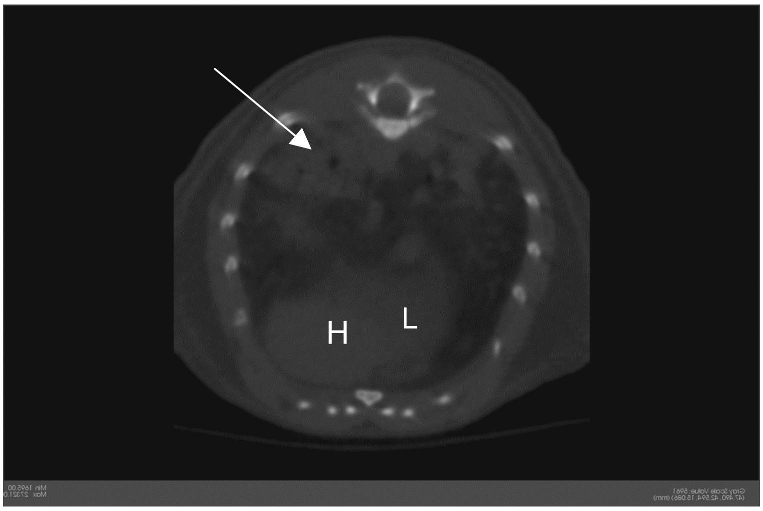

Transversal section of lung CT-scan corresponding to *Trf1* $^{+/+}$ *K-ras* $^{+/G12V}$ *p53* $^{-/-}$ treated mouse shown in Figure 8b (below). The arrow indicates a tumor surrounded by high inflammation that impeeds the accurate meassurement of its size. H (heart) and L (Liver).

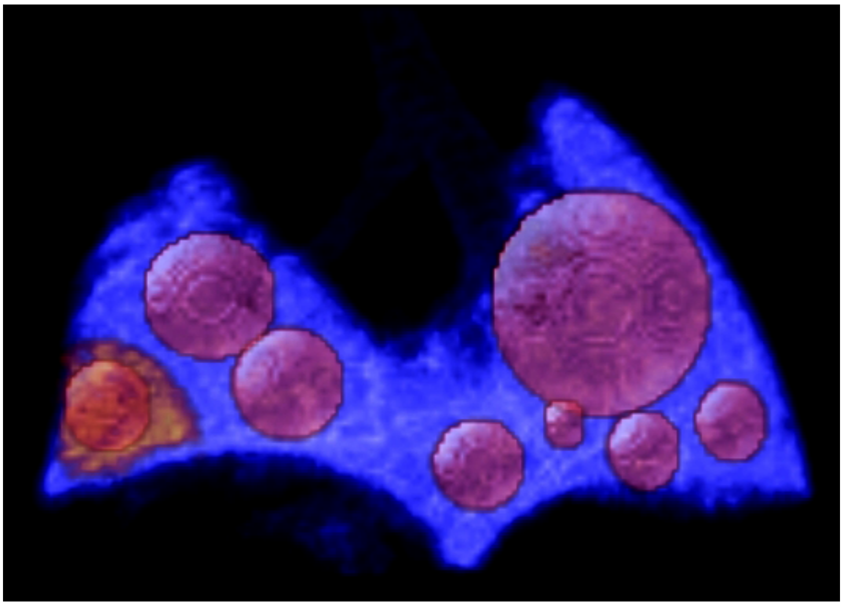

Figure 2 for reviewer

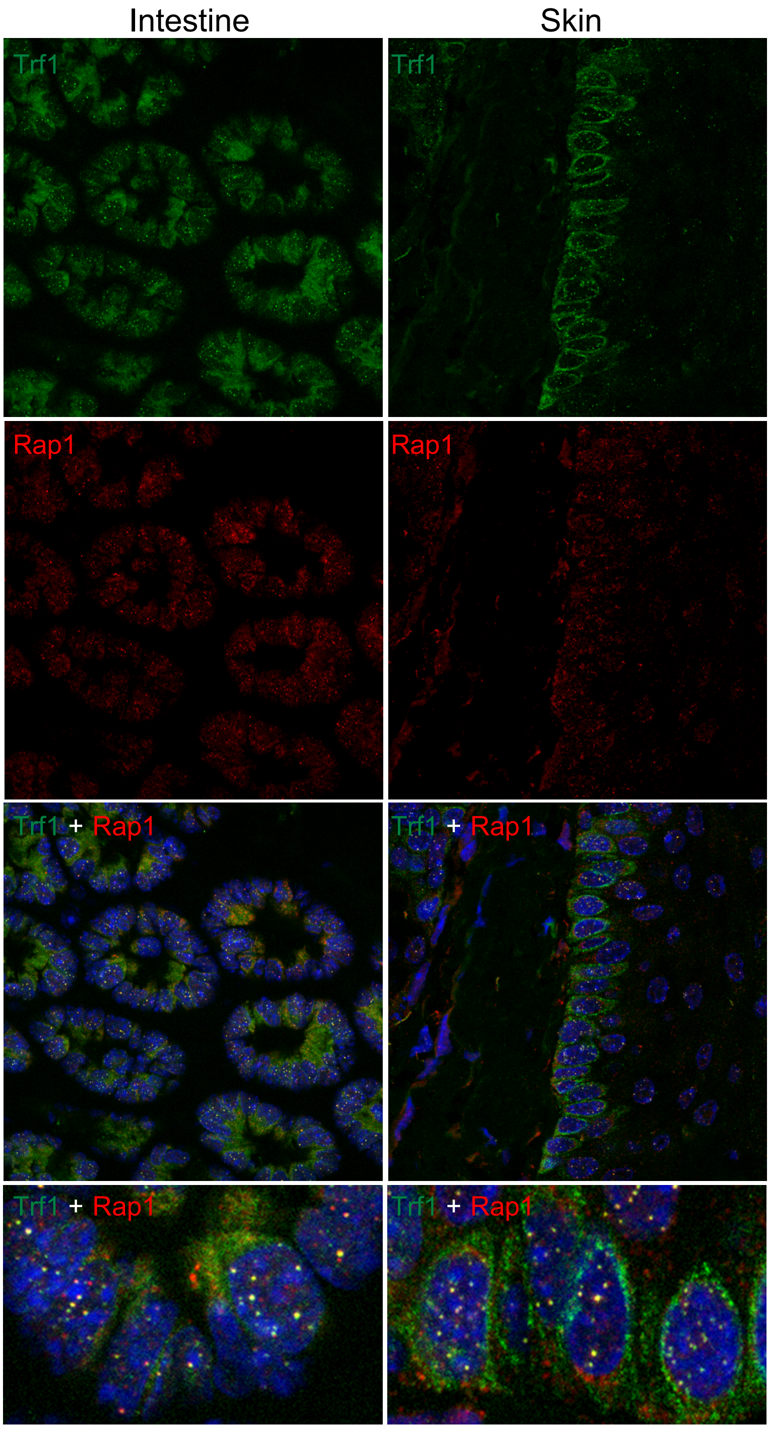

Representative images of Trf1 and Rap1 double immunofluorescence in intestine and skin sections of wild type animals. In the lowest panels a higher magnification images are shown.

Figure 3 for reviewer

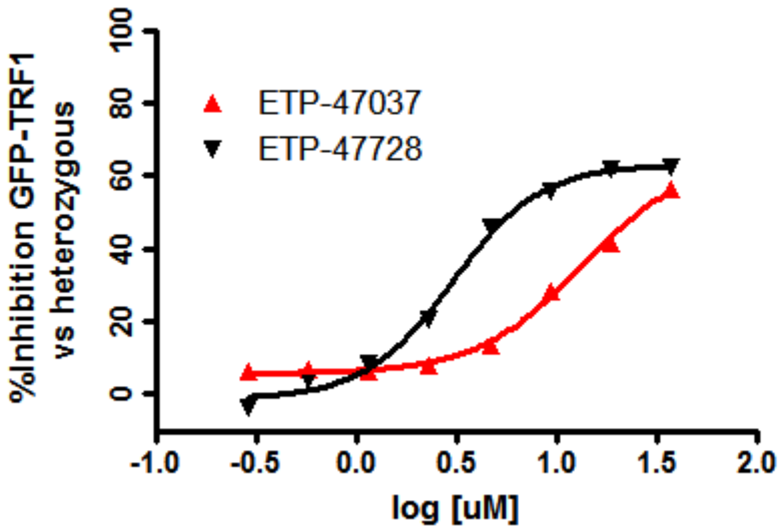

Inhibition of Trf1 at different concentrations of ETP-47037 and ETP-47228

7th Editorial Decision 27March 2015

Thank you for the submission of your revised manuscript to EMBO Molecular Medicine. I am pleased to inform you that we will be able to accept your manuscript pending the following final Editorial amendments:

1) As per our Author Guidelines, the description of all reported data that includes statistical testing must state the name of the statistical test used to generate error bars and P values, the number (n) of independent experiments underlying each data point (not replicate measures of one sample), and the actual P value for each test (not merely 'significant' or 'P < 0.05'). Should you feel that this causes overcrowding of figures, or simply prefer to do so, you may simply provide a list of all P values in table format as a supplementary table. Some Authors choose to do this and it is perfectly acceptable as long as you refer to the table where appropriate in the main text and/or legends.

2) I note that Fig. 8 is distributed on two different pages. As each main figure must fit in one page, I must ask you therefore to either consolidate Fig. 8 into one page or split into two separate figures (thus generating a new Fig. 9). I'm afraid that the latter solution appears to be your best bet as the number of panels is rather high. Again, please make sure that manuscript text is consequently and accurately amended to reflect the new figure. Also, please remove the red lettering from your manuscript text as this is no longer needed.

3) Please remove the "Statement of Significance" section from the manuscript. All EMBO Molecular Medicine research articles are instead accompanied by "The Paper Explained", a structured summary of the article to emphasize the major findings of the paper and their medical implications for the non-specialist reader. Please provide a summary accessible to non-specialists and specialists alike, highlighting the medical issue you are addressing (heading: PROBLEM), the results obtained (heading: RESULTS), and their clinical impact (heading: IMPACT). This may be edited to ensure that readers understand the significance and context of the research. Please refer to any of our published research articles for an example (embomolmed.org).

4) Please make sure that for this final version you a) upload a .doc formatted version of the manuscript text (including Figure legends), b) separate figure files* and c) a separate single PDF file of the Supplementary information

5) We are now encouraging the publication of source data, particularly for electrophoretic gels and blots, with the aim of making primary data more accessible and transparent to the reader. Would you be willing to provide a PDF file per figure that contains the original, uncropped and unprocessed scans of all or at least the key gels used in the manuscript? The PDF files should be labeled with the appropriate figure/panel number, and should have molecular weight markers; further annotation may be useful but is not essential. The PDF files will be published online with the article as supplementary "Source Data" files. If you have any questions regarding this just contact me.

6) Every published paper includes a 'Synopsis' to further enhance discoverability. Synopses are displayed on the journal webpage and are freely accessible to all readers. They include a short standfirst as well as 2-5 one sentence bullet points that summarise the paper. Please provide the synopsis including the short list of bullet points that summarise the key NEW findings. The bullet points should be designed to be complementary to the abstract - i.e. not repeat the same text. We encourage inclusion of key acronyms and quantitative information. Please use the passive voice. Please attach this information in a separate file or send directly by email, we will incorporate it accordingly. Again, please refer to any of our published research articles for an example (embomolmed.org). You are also welcome to suggest a striking image or visual abstract to illustrate your article. If you do please provide a jpeg file 550 px-wide x 400-px high.

Please submit your revised manuscript within two weeks. I look forward to seeing the final version of your manuscript as soon as possible.
